# Targeting TRPV6/CXCR4 complexes prevents castration-resistant prostate cancer metastasis to the bone

**DOI:** 10.1038/s41392-025-02376-8

**Published:** 2025-09-05

**Authors:** Clément Cordier, Aurélien Haustrate, Adriana Mihalache, Erika Duval, Emilie Desruelles, Corentin Spriet, Baptiste Casel, Lotfi Slimani, Benjamin Soret, Laurent Allart, George Shapovalov, Pierre Gosset, Natalia Prevarskaya, V’yacheslav Lehen’kyi

**Affiliations:** 1 Laboratory of Cell Physiology, INSERM U1003, Laboratory of Excellence Ion Channels Science and Therapeutics, Equipe Labellisée par la Ligue Nationale Contre le Cancer. Department of Biology, Faculty of Science and Technologies, University of Lille, Villeneuve d’Ascq, France; 2 Pathology Department, Saint Vincent de Paul Hospital, Hospital Group of Catholic Institute of Lille (GHICL), Lille, France; 3 University of Lille, CNRS, Inserm, CHU Lille, Institut Pasteur of Lille, US 41 - UAR 2014 - PLBS, Lille, France; 4 University of Paris Cité and Sorbonne Paris Nord, Inserm UMR1333, Oral Health, Montrouge, France

**Keywords:** Metastasis, Urological cancer

## Abstract

Bone metastasis most commonly occurs in castration-resistant prostate cancer (CRPC). The TRPV6 calcium channel is absent in healthy prostate tissue, but its expression increases considerably during cancer progression. We hypothesized that cancer cells induce TRPV6 expression de novo to directly benefit from tightly regulated calcium intake *via* TRPV6 while providing cancer cells with a selective advantage for metastasis in the calcium-abundant niche, such as bone. Using a cohort of prostate cancer tissue biopsies from patients with a clinical history of at least 10 years after biopsy, we report that TRPV6 expression directly correlates with CRPC tumor aggressiveness and increased risk of metastasis development. The TRPV6 channel is involved in the acquisition of both mesenchymal and invasive phenotypes through increased phosphorylation of CaMK2 followed by the translocation of the transcription factor NF-κB to the nucleus and the expression of EMT markers, MMPs, and transcription factors such as Twist, Snail, and Slug. Moreover, TRPV6 expression was accompanied by increased formation of CXCR4/TRPV6 complexes. In vivo, mice bearing *trpv6*^*+/+*^ tumors presented increased metastasis, notably bone metastasis, whereas *trpv6*^*−/−*^ mice developed no metastasis. Targeting TRPV6 with a monoclonal antibody resulted in a significant reduction in the metastatic burden and an increase in overall survival. When AMD3100, a selective inhibitor of the CXCR4 receptor, was combined with AMD3100, a synergistic effect on the suppression of metastasis development was achieved. Thus, the suppression of CRPC metastasis to bone can be achieved *via* simultaneous targeting of TRPV6/CXCR4, demonstrating that combined therapy is a proof-of-concept approach in vivo.

## Introduction

The ability of malignant cells to leave the primary tumor site and spread to other, more or less distant parts of the body, forming another tumor type broadly known as metastasis, makes cancer one of the world’s leading causes of death.^1^ Metastasis is the result of a variety of complex and integrated cellular behaviors.^2^ Tumor cells initially invade tissues surrounding the primary tumor, resulting in so-called local metastasis. These malignant cells then enter the bloodstream, either directly or via the lymphatic system, before either adhering to the vessel wall or stopping at the first capillary bed encountered.^3^ Tumor cells then extravasate the bloodstream to land in a secondary organ with a more or less prepared microenvironment, according to Piaget’s “seed-and-soil theory”.^4,5^

One of the particularly common sites for metastasis is bone, which affects many patients with advanced cancer.^6^ Bone metastasis often results in skeletal morbidity, commonly referred to as skeletal-related events (SREs), which are the main complications of tumor-related bone disease.^7^ Indeed, bone metastasis can be of two types on the basis of the predominance of lysis or sclerosis in the bone and is therefore characterized as osteolytic or osteoblastic, respectively, depending on the radiographic appearance of the lesions.^3,8^

Metastatic bone disease is the most common type of cancer,^9^ including multiple types of melanoma (40%), prostate (85%), and breast (70%), as well as lung (40%) and kidney (40%)^10,11^ cancer. However, given the high prevalence of breast, lung and prostate cancers, these carcinomas represent more than 80% of patients with metastatic bone diseases.

Prostate cancer (PCa) is one of the most common cancers in men worldwide, behind lung cancer in incidence and fifth in mortality. According to statistics from the World Cancer Observatory (http://gco.iarc.fr/), PCa affected 1,414,259 new cases worldwide in 2020, particularly in industrialized countries, with 3/4 cases in men over 65 yo.^12^ Initially, tumors are androgen dependent but become insensitive to androgens in 15% of cases, which qualifies them as castration-resistant prostate cancers (CRPCs).^13^ CRPCs are highly invasive and easily spread to other tissues, resulting in mortality in CRPC patients and partly explaining the decline in the 5-year survival rate of PCa patients.^9^ This increase in mortality rates among patients with CRPC is explained by the fact that 72% of them eventually develop androgen-resistant bone metastases, while therapeutic options remain limited and inefficient. In the majority of cases, bone metastases in patients with CRPC lead to death, with the median survival for patients with CRPC with bone metastases being 21 months,^14^ making CRPC one of the principal causes of mortality in patients with prostate cancer.

Bone is a rich source of calcium, which can be released as a result of bone destruction, whereas extracellular calcium has been shown to promote tumor growth in bone through targeting extracellular receptors and/or specific calcium channels to the cancer cell surface.^15^ Calcium is also known to regulate 1/3 of the genome^16^ in addition to interacting with various proteins, such as CaM, CaMK, MAPK, and Akt, which can phosphorylate several known cell survival signaling pathways, such as NF-κB and NFAT.^17–19^

Epithelial tissue, which is a common origin of various cancers, is known to express a variety of nonvoltage-regulated families of ion channels, called the transient receptor potential (TRP) superfamily.^20,21^ Among these channels, the TRPV6 channel is the most selective for Ca^2+^, with *P*_Ca_/*P*_Na_ values >100, and plays a crucial role in calcium homeostasis in various cell types,^22,23^ making this channel closely involved in intracellular Ca^2+^-related pathways as well as in bone mineralization.^24,25^ TRPV6 overexpression in human malignancies has been established in thyroid, colon, breast, ovarian, and prostate carcinomas and is associated with tumor aggressiveness.^26^ It has been previously hypothesized via a transcriptomics approach that the TRPV6 channel seems to regulate Ca^2+^ signaling pathways as well as the expression of Ca^2+^-permeable channels and other crucial pathways for the development of an aggressive and invasive phenotype in castration-resistant prostate cancer (CRPC).^27^ However, no detailed studies characterizing the involvement of TRPV6 in the migratory and invasive potential of CRPCs, not its involvement in bone tropism, have yet been published.

We hypothesized that prostate cancer cells induce TRPV6 expression de novo to directly benefit from tightly regulated calcium intake *via* TRPV6, which is required for the calcium-dependent aggressive phenotype, such as migration and invasion. In addition, the expression of the TRPV6 channel provides cancer cells with a selective advantage because of its metastatic potential in the calcium-abundant niche, such as bone. Thus, three *trpv6*^−/−^ cell line models, HAP-1^*trpv6−/−*^, PC-3M^*trpv6−/−*^ and PC-3M-luc-C6^*trpv6−/−*^, were generated and studied both in vitro and in vivo to explore the involvement of TRPV6 in the migratory and invasive potential of prostate cancer cells as well as the calcium-regulated signaling pathways involved therein. A correlation study of TRPV6 expression associated with metastatic formation in 37 human patients was performed, and its role in in vivo metastasis, including bone, was studied. Finally, we demonstrated the use of a previously published monoclonal antibody against the TRPV6 channel^28^ for the prevention of metastasis formation in vivo.

## Results

### TRPV6 expression directly correlates with metastasis-associated prostate cancer

**Fig. 1:**
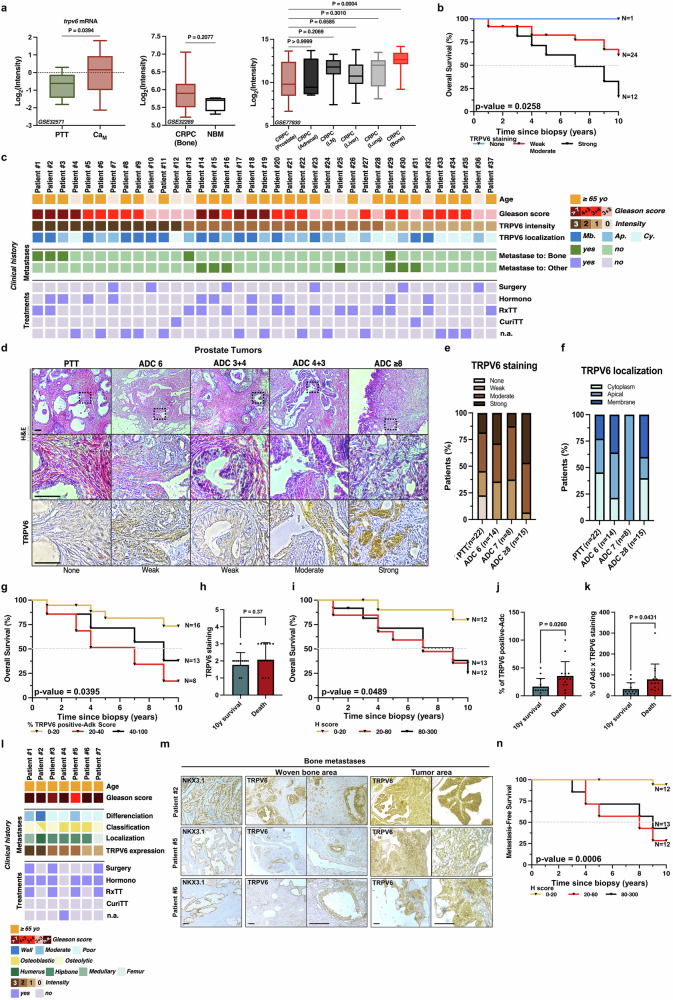
Human TRPV6 expression in prostate metastatic cancer. **a** Expression intensity of the *trpv6* gene from published gene expression datasets (GEO: GSE32571; GSE32269; GSE77930). PTT: peritumoral tissue; Ca_M_: PCa metastasis; CRPC (bone): bone metastasis from castration-resistant prostate cancer (CRPC); NBM: normal bone marrow. **b** OS as a function of TRPV6 expression: no TRPV6 staining (*n* = 1); weak and moderate TRPV6 staining (*n* = 24); and strong TRPV6 staining (*n* = 12) in human patients according to the log rank (Mantel‒Cox) test. **c** Clinical history of 37 human patients with prostate cancer biopsies: n.a. not available; Mb. membrane; Ap. apical; Cy. cytosolic staining. **d** Representative images of both H&E and TRPV6 staining in prostate tumor biopsies: peritumoral tissue (PTT) (*n* = 22), adenocarcinomas (ADCs) with Gleason scores of 3 + 3 (*n* = 14), Gleason scores of 3 + 4 (*n* = 4), Gleason scores of 4 + 3 (*n* = 4), and Gleason scores ≥ 8 (*n* = 15). Scale bar, 100 μm. **e** Quantification of TRPV6 staining intensities in prostate tumor biopsies from (**d**). **f** Localization of TRPV6 staining in prostate tumor biopsies from (**d**). **g** Overall survival rate of 0–20% of TRPV6-positive adenocarcinomas (*n* = 16), 20–40% of TRPV6-positive adenocarcinomas (*n* = 8), and 40–100% of TRPV6-positive adenocarcinomas (*n* = 13) in human patients according to the log-rank (Mantel–Cox) test. **h** 10-year survival rate of patients following biopsy according to TRPV6 staining. **i** OS of human patients according to the *H* score, according to the log rank (Mantel‒Cox) test: 0–20 (*n* = 12), 20–80 (*n* = 13), and 80–300 (*n* = 12). **j** Ten-year death and survival rates compared with the percentage of TRPV6-positive adenocarcinomas in the biopsies. **k** The 10-year death and survival rates compared with the *H*-scores of the biopsies were determined via an arbitrary score. **l** Clinical history of 7 patients with bone metastasis originating from prostate cancer. n.a. not available. **m** Representative images of both NKX3.1 and TRPV6 staining in bone metastases of prostate tumor origin via differentiation stage-matched intensity comparisons. Scale bars, 100 μm. **n** Metastasis-free survival of human patients according to arbitrary scores of 0–20 (*n* = 12), 20–80 (*n* = 13), and 80–300 (*n* = 12) according to the log rank (Mantel‒Cox) test. Mean ± SEM (**a**, **h**, **j**, **k**). Two-tailed t test (**a**, **h**, **j**, **k**). Two-way ANOVA (**a**). Log rank (Mantel‒Cox) test (**b**, **g**, **i**, **n**)

The expression of *trpv6* was originally studied via the prostate cancer database (PCaDB), which comprises various sample types, including peritumoral tissue (PTT), PCa metastasis (Ca_M_), castration-resistant prostate cancer (CRPC), and bone metastasis from CRPC as well as nonbone metastasis peritumoral tissue (NBM) (GEO: GSE32571; GSE32269; GSE77930) (Fig. 1). *Trpv6* expression was shown to be significantly upregulated (*p* < 0.0394) in primary PCa metastases and was significantly increased (*p* < 0.0004) in CRPC metastasized into bone tissue compared with CRPC of the prostate (Fig. 1a). Even in CRPC bone, the *trpv6* level was higher, although not significantly (*p* = 0.21), than that in normal bone marrow.

A retrospective cohort of 37 prostate cancer tissue biopsies, with a clinical history of patients for at least 10 years after biopsy, was constructed to establish the potential role of TRPV6 in patient outcomes (Fig. 1b). At the time of diagnosis, patients had an average age of 68.1 years (ranging from 50 to 85 years). The overall survival of these patients, as a function of the level of TRPV6 expression, revealed a direct correlation of *p* = 0.0258 according to the log-rank (Mantel‒Cox) test (Fig. 1b).

Furthermore, 37 human prostate tissue samples were analyzed via IHC to study TRPV6 expression and its intensity and correlation with bone metastasis occurrence as well as the therapeutic approach used for each of the 37 patients (Fig. 1c). The upregulation of TRPV6 during cancer progression is clearly associated with a tight correlation with decreased patient survival (Fig. 1b–d). Despite its expression in clinically localized prostate cancer in both low Gleason score tumors (≤6) and high Gleason score tumors (≥9 or 10), its expression was greater in groups with higher Gleason scores (Fig. 1e).

With respect to its subcellular localization, TRPV6 has both apical and membrane expression, which increases with tumor progression (Fig. 1f). Intriguingly, analysis of the PTT revealed overall TRPV6 channel expression in 77.27% of the patients, with cytosolic expression in 45.45% of the patients and membrane expression in only 22.72% of the patients (Fig. 1e and f). These data demonstrate that TRPV6 expression and localization might have clinical relevance, suggesting that TRPV6 may contribute to the malignancy of prostate carcinomas.

The proportion of adenocarcinomas expressing TRPV6 was directly linked (*p* = 0.0395) to a significant reduction in the survival of patients, with a decrease of 82.86% in 10 years for the 20–40% group of TRPV6-positive adenocarcinomas (*p* = 0.0110) and a decrease of 61.91% for the 40–100% group (*p* = 0.0369) of TRPV6-positive adenocarcinomas (Fig. 1g). Since the intensity of TRPV6 staining alone was not enough to provide a significant difference (*p* = 0.37) in patient outcomes over 10 years (Fig. 1h), a combined score, i.e., the percentage of TRPV6-expressing adenocarcinomas multiplied by TRPV6 staining, was used as a histological score (H score), which is a more reliable histoprognostic marker of patient survival. This score ranged from 0 to 300, where 300 was used to define strong expression with an intensity of 3 in 100% of the adenocarcinomas (*p* = 0.049) (Fig. 1i). As such, patient survival decreased significantly starting from an *H* score of 20–80 and above, reaching a survival of 25.46% at 10 years in both groups (Fig. 1i). Notably, the 10-year death/survival rate was significantly correlated (*p* = 0.026) with the percentage of TRPV6-positive adenocarcinomas in the biopsies according to either the intensity-only or combined *H*-score (*p* = 0.04) (Fig. 1j, k).

Furthermore, TRPV6 expression was evaluated in 7 biopsies of bone metastases from subjects aged over 65 years, with osteolytic, osteoblastic or mixed-type metastases showing moderate to strong intensity (Fig. 1l and m). As such, metastasis occurrence is directly associated (*p* = 0.0006) with the percentage of TRPV6-positive adenocarcinomas as low as 20 years (H score), beyond which TRPV6 is a significant indicator (*p* = 0.0006) of metastasis development over 10 years, ranging from 5% risk to 80% risk (Fig. 1n).

These data clearly suggest the direct role of the TRPV6 channel in the development of metastasis, highlighting the importance of evaluating both the intensity of staining and the percentage of TRPV6 expression in adenocarcinomas for an accurate prognosis of metastatic development. Moreover, the data suggest that the PTT regions expressing the TRPV6 channel de novo are not reliable controls for the prediction of an aggressive phenotype and an increased risk of relapse.

### TRPV6 activity promotes the migration and invasion of prostate cancer cells

To demonstrate the migratory and invasive potential of the TRPV6 calcium channel in CRPC cells, we used a panel of six cell lines, both *trpv6*^*+/+*^ and *trpv6*^*−/−*^, such as four highly aggressive CRPC cell lines, PC-3M, VCaP, and LNCaP-C4-2B, derived from metastases,^29–31^ as well as a bone metastasis model, PC-3M-luc-C6, with strong bone tropism^,^^32^ enabling us to monitor tumor development *via* bioluminescence in vivo imaging systems (Supplementary Table 1). One castration-sensitive cell line (CSPC), LNCaP, was used.^33^ The other cell line was HAP-1 derived from chronic myelogenous leukemia, which represents a nonprostatic phenotype. The *trpv6*^*−/−*^ cell clones from these cell lines were developed via the CRISPR-CAS9 approach. All of them were stably transfected with mCherry for further in vivo imaging (-pmCherry). They were then stably transfected with either the empty vector or with TRPV6_wt_ (-pTRPV6_wt_) or TRPV6 pore mutant TRPV6^D582A^, which is known for its nonconductance of ion flux,^34^ thus providing an additional control for the involvement of TRPV6 as an ion channel. All these cell lines were confirmed in vitro for expression of the TRPV6 channel at both the mRNA and protein levels (Supplementary Figs. 1a–d, 4a and b, 5a, i and q) and validated functionally via a store-operated calcium entry (SOCE) test, where TRPV6 was previously shown to play a crucial role as an SOCE enhancer^35^ (Supplementary Figs. 1e–l, 4c–f, 5b, j and r). Moreover, the expression of other SOCE players, such as Orai1, STIM1 and TRPC1, was controlled in both PC-3M and PC-3M-luc-C6 stable clones (Supplementary Fig. 1m and n). Finally, the ability of TRPV6 to function as a channel (both TRPV6_wt_ and TRPV6^D582A^) to rescue stable *trpv6*^*−/−*^ clones was analyzed via electrophysiological experiments with a whole-cell patch‒clamp configuration (Supplementary Fig. 1o).

**Fig. 2:**
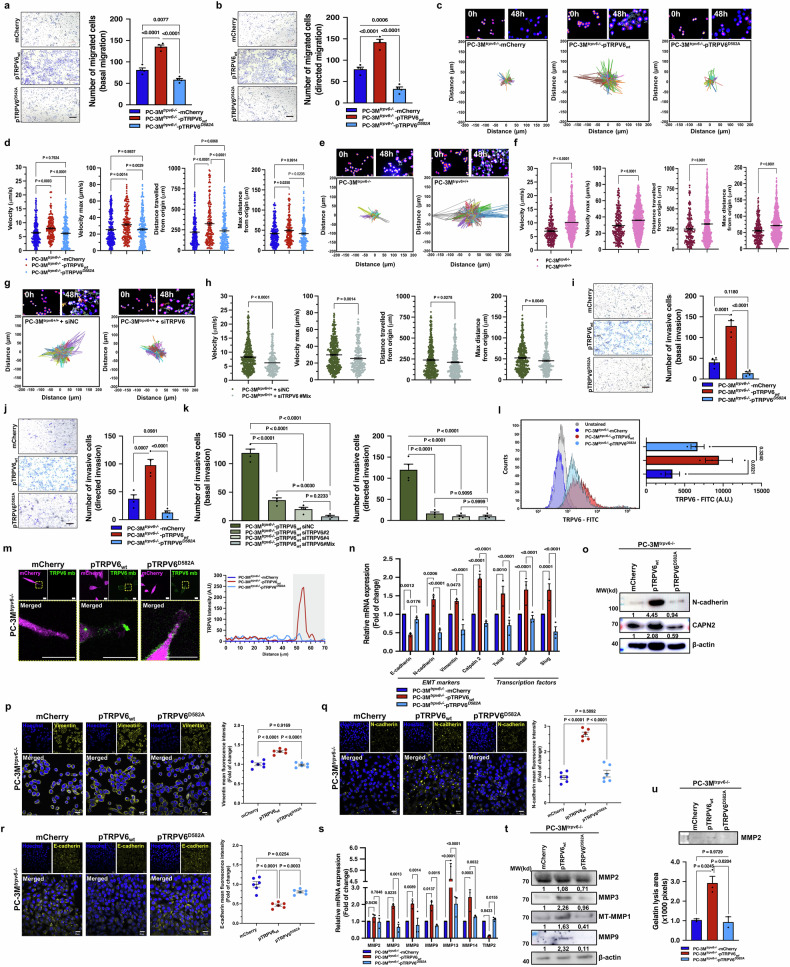
TRPV6 is involved in cancer cell migration and invasion in vitro. **a** Basal migration of PC-3M^*trpv6−/−*^-mCherry, PC-3M^*trpv6−/−*^-pTRPV6_wt_, and PC-3M^*trpv6−/−*^-pTRPV6^D582A^ stable cell clones. Representative images and quantification of migrated cells (*n* = 4). Scale bar, 200 μm. **b** Directed migration of PC-3M^*trpv6−/−*^-mCherry, PC-3M^*trpv6−/−*^-pTRPV6_wt_, and PC-3M^*trpv6−/−*^-pTRPV6^D582A^ stable cell clones shown as representative images and quantification of migrated cells (*n* = 4). Scale bar, 200 μm. **c** Tracking of PC-3M^*trpv6−/−*^-mCherry (*n* = 29), PC-3M^*trpv6−/−*^-pTRPV6_wt_ (*n* = 46), and PC-3M^*trpv6−/−*^-pTRPV6^D582A^ (*n* = 30) stable cell clones for 48 h. **d** Quantification of both the cell velocity and distance of stable cell clones from (**c**). **e** PC-3M^*trpv6−/−*^ (*n* = 63) and PC-3M^*trpv6+/+*^ (*n* = 115) cells were tracked for 48 h. **f** Quantification of both the cell velocity and distance of prostate cancer cells from (**e**). **g** Tracking of PC-3M^*trpv6+/+*^ cells treated with 40 nM of either the negative control siRNA (*n* = 86) or a mixture of siRNAs against TRPV6 (*n* = 46) for 48 h. **h** Quantification of both the cell velocity and distance of prostate cancer cells from (**g**). **i** Basal invasion of PC-3M^*trpv6−/−*^-mCherry, PC-3M^*trpv6−/−*^-pTRPV6_wt_, and PC-3M^*trpv6−/−*^-pTRPV6^D582A^ stable cell clones shown as representative images and quantification of migrated cells (*n* = 4). Scale bar, 200 μm. **j** Directed invasion of PC-3M^*trpv6−/−*^-mCherry, PC-3M^*trpv6−/−*^-pTRPV6_wt_, and PC-3M^*trpv6−/−*^-pTRPV6^D582A^ stable cell clones shown as representative images and quantification of migrated cells (*n* = 4). Scale bar, 200 μm. **k** Basal and direct invasion of PC-3M^*trpv6−/−*^-pTRPV6_wt_ cells treated with 40 nM control (siNC) or specific siTRPV6. Quantification of invasive cells (*n* = 4). **l** Flow cytometry analysis of cell surface TRPV6 expression in PC-3M^*trpv6−/−*^-mCherry (*n* = 29), PC-3M^*trpv6−/−*^-pTRPV6_wt_ (*n* = 46), and PC-3M^*trpv6−/−*^-pTRPV6^D582A^ stable cell clones. **m** TRPV6 channel targeting the plasma membrane in the regions of cell protrusions in PC-3M^*trpv6−/−*^-mCherry, PC-3M^*trpv6−/−*^-pTRPV6_wt_, and PC-3M^*trpv6−/−*^-pTRPV6^D582A^ stable cell clones. Scale bars, 100 μm. **n** EMT marker and transcription factor expression via qPCR in PC-3M^*trpv6−/−*^-mCherry, PC-3M^*trpv6−/−*^-pTRPV6_wt_, and PC-3M^*trpv6−/−*^-pTRPV6^D582A^ stable cell clones (*n* = 3). **o** Western blot analysis of N-cadherin and calpain-2 proteins expression in PC-3M^*trpv6−/−*^ and PC-3M-luc-C6^*trpv6+/+*^ stable cell clones compared with TRPV6 expression. **p** Vimentin staining in PC-3M^*trpv6−/−*^-mCherry, PC-3M^*trpv6−/−*^-pTRPV6_wt_, and PC-3M^*trpv6−/−*^-pTRPV6^D582A^ stable cell clones with the quantification of the mean intensity reported via Hoechst staining (*n* = 6). Scale bar, 20 μm. **q** N-cadherin staining in PC-3M^*trpv6−/−*^-mCherry, PC-3M^*trpv6−/−*^-pTRPV6_wt_, and PC-3M^*trpv6−/−*^-pTRPV6^D582A^ stable cell clones with the quantification of the mean intensity reported via Hoechst staining (*n* = 6). Scale bar, 20 μm. **r** E-cadherin staining in PC-3M^*trpv6−/−*^-mCherry, PC-3M^*trpv6−/−*^-pTRPV6_wt_, and PC-3M^*trpv6−/−*^-pTRPV6^D582A^ stable cell clones with the quantification of the mean intensity reported via Hoechst staining (*n* = 6). Scale bar, 20 μm. **s** MMPs and TIMP2 expression via qPCR in PC-3M^*trpv6−/−*^-mCherry, PC-3M^*trpv6−/−*^-pTRPV6_wt_, and PC-3M^*trpv6−/−*^-pTRPV6^D582A^ stable cell clones (*n* = 3). **t** Protein expression of the MMP2, MMP3, MT-MMP1, and MMP9 proteins in PC-3M^*trpv6-/-*^ and PC-3M-luc-C6^*trpv6+/+*^ stable cell clones compared with β-actin expression and TRPV6 status. **u** 1% gelatin zymography analysis and quantification of MMP2 in the conditioned media of PC-3M^*trpv6−/−*^-mCherry, PC-3M^*trpv6−/−*^-pTRPV6_wt_, and PC-3M^*trpv6−/−*^-pTRPV6^D582A^ stable cell clones. Mean ± SEM (**a**, **b**, **d**, **f**, **h–l**, **n**, **p–s**, **u**). Two-tailed *t*-test (**f**, **h**). Two-way ANOVA (**a**, **b**, **d**, **i–l**, **n**, **p–s**, **u**). See also Supplementary Figs. 1 and 2

Once established and validated in vitro, our stable clones were subjected to further studies on the involvement of the TRPV6 calcium channel in the acquisition of an aggressive phenotype. Both basal and directed migration were affected, indicating that the presence of functional TRPV6 channels in the cell phenotype significantly (*p* < 0.0001) enhanced both basal and directed CRPC cell migration, as determined via a transwell® assay (Fig. 2a and b). The same effects were observed for the PC-3M-luc-C6 (Supplementary Fig. 2a and b) and HAP-1 (Supplementary Fig. 4g) stable cell clones VCaP (Supplementary Fig. 5c), LNCaP-C4-2B (Supplementary Fig. 5k), and LNCaP (Supplementary Fig. 5s). The data were confirmed via a wound-healing assay for the same stable clones, such as PC-3M^*trpv6−/−*^-mCherry, PC-3M^*trpv6−/−*^-pTRPV6_wt_, and PC-3M^*trpv6−/−*^-pTRPV6^D582A^ (Supplementary Fig. 2c and d); PC-3M-luc-C6^*trpv6+/+*^-mCherry and PC-3M-luc-C6^*trpv6+/+*^-pTRPV6_wt_ (Supplementary Fig. 2e and f); and HAP-1^*trpv6−/−*^-mCherry, HAP-1^*trpv6−/−*^-pTRPV6_wt_, and HAP-1^*trpv6−/−*^-pTRPV6^D582A^ (Supplementary Fig. 4h) stable cell clones.

Increased motility is a hallmark of cancer cell aggressiveness. While CRPC cell motility was being studied, data similar to the effects of the TRPV6 channel on both average and maximal cell velocity, as well as the roaming distance, while PC-3M^*trpv6−/−*^-mCherry, PC-3M^*trpv6−/−*^-pTRPV6_wt_, and PC-3M^*trpv6−/−*^-pTRPV6^D582A^ stable cell clones were tracked for 48 h (Fig. 2c and d). Identical effects were obtained for the PC-3M-luc-C6 (Supplementary Fig. 2g) and HAP-1 (Supplementary Fig. 4i and j) cell lines and their peer clones. The effects of TRPV6 rescuing into the PC-3M^*trpv6−/−*^, PC-3M-luc-C6^*trpv6−/−*^, and HAP-1^*trpv6−/−*^ cell lines were confirmed via the use of different stable clones, and a control experiment in which PC-3M^*trpv6−/−*^ versus wild-type PC-3M^*trpv6+/+*^ cells yielded similar effects (Fig. 2e and f). Intriguingly, transient transfection of the TRPV6 channel into the PNTA1 cell line (immortalized prostatic cell line (Supplementary Fig. 4k)) had similar effects on both direct migration and invasion (Supplementary Fig. 4l and m). Finally, siRNA-mediated knockdown of *trpv6* (preliminarily validated in PC-3M^*trpv6−/−*^-pTRPV6_wt_ (Supplementary Fig. 2h) and PC-3M-luc-C6^*trpv6+/+*^-pTRPV6_wt_ (Supplementary Fig. 2i)) was used to determine the effects of TRPV6 on CRPC cell motility/directed migration. Tracking of PC-3M^*trpv6+/+*^ cells treated with 40 nM mixed siRNAs against *trpv6* for 48 h is shown in Fig. 2g, h; similar effects were demonstrated in PC-3M^*trpv6−/−*^-pTRPV6_wt_ (Supplementary Fig. 2j) and PC-3M-luc-C6^*trpv6+/+*^-pTRPV6_wt_ (Supplementary Fig. 2k) stable cell clones, confirming that TRPV6 is a key player in CRPC cell motility/directed migration.

The role of TRPV6 in CRPC cell invasion was studied via Transwell® inserts (Falcon™) precoated with a Matrigel™ basement membrane matrix. Both basal and directed (2–10% FBS gradient) invasion assays revealed strong involvement of the TRPV6 channel in matrix invasion, as shown in PC-3M^*trpv6−/−*^-mCherry, PC-3M^*trpv6−/−*^-pTRPV6_wt_, and PC-3M^*trpv6−/−*^-pTRPV6^D582A^ (Fig. 2i and j); PC-3M-luc-C6^*trpv6+/+*^-mCherry and PC-3M-luc-C6^*trpv6+/+*^-pTRPV6_wt_ (Supplementary Fig. 2l and m); HAP-1^*trpv6−/−*^-mCherry, HAP-1^*trpv6−/−*^-pTRPV6_wt_, and HAP-1^*trpv6−/−*^-pTRPV6^D582A^ (Supplementary Fig. 4n) stable cell clones; and VCaP^*trpv6+/+*^ cells treated with siTRPV6 (Supplementary Fig. 5d), LNCaP-C4-2B^*trpv6+/+*^-mCherry and LNCaP-C4-2B^*trpv6+/+*^-pTRPV6_wt_ (Supplementary Fig. 5l), and LNCaP^*trpv6+/+*^ cells treated with siTRPV6 (Supplementary Fig. 5t).

In addition, both basal and direct invasion of PC-3M^*trpv6−/−*^-pTRPV6_wt_ (Fig. 2k) and PC-3M-luc-C6^*trpv6+/+*^-pTRPV6_wt_ (Supplementary Fig. 2y) stable cell clones were affected by the selective 40 nM siRNA against *trpv6* for 48 h. However, the data clearly revealed that TRPV6 functions as a functional channel (no phenotype was rescued with the TRPV6 pore mutant TRPV6^D582A^). Its membrane expression was evaluated via a FACS assay in PC-3M^*trpv6−/−*^-derived clones (Fig. 2l) and PC-3M-luc-C6^*trpv6−/−*^-derived (Supplementary Fig. 2o and p) stable clones. Only functional TRPV6 channels were found on the plasma membrane and, more precisely, in the front regions of the protrusions of both PC-3M^*trpv6−/−*^-derived (Fig. 2m) and PC-3M-luc-C6^*trpv6−/−*^-derived (Supplementary Fig. 2q) clones.

Since both the migratory and invasive potential of CRPC cells result from the acquisition of a mesenchymal phenotype through epithelial‒mesenchymal transition (EMT), key EMT markers and transcription factors were studied in PC-3M^*trpv6−/−*^-derived and PC-3M-luc-C6^*trpv6−/−*^-derived stable cell clones at both the mRNA and protein levels. Our data strongly indicated that CRPC cells expressing functional TRPV6 channels have increased expression of N-cadherin, vimentin, and calpain 2 proteins together with transcription factors, such as Twist, Snail, and Slug, in PC-3M^*trpv6−/−*^-derived (Fig. 2n) and PC-3 M-luc-C6^*trpv6−/−*^-derived (Supplementary Fig. 2r) stable clones. In contrast, the expression of E-cadherin was significantly decreased in PC-3M^*trpv6−/−*^-derived clones (*p* = 0.0012). The protein levels of N-cadherin (Fig. 2o and q) and vimentin (Fig. 2p) were significantly greater (*p* < 0.0001 for both) than those of E-cadherin (Fig. 2r), whose expression was significantly lower (*p* < 0.0001) in PC-3M^*trpv6−/−*^-derived stable clones. The same protein pattern was observed in PC-3M-luc-C6^*trpv6−/−*^-derived stable cell clones (Supplementary Fig. 2s–v), suggesting the direct role of the TRPV6 channel in EMT.

As various matrix metalloproteinases (MMPs) play crucial roles in CRPC cell invasion, a panel of MMPs was studied in CRPC cell models. The significantly increased expression of MMP3, 8, 9, 13, and 14 together with the decreased expression of the inhibitor TIMP2 were detected in PC-3M^*trpv6−/−*^-mCherry, PC-3M^*trpv6−/−*^-pTRPV6_wt_, and PC-3M^*trpv6−/−*^-pTRPV6^D582A^ stable cell clones at both the mRNA (Fig. 2s) and protein levels (Fig. 2t) and were similar to those in the PC-3 M-luc-C6^*trpv6+/+*^-mCherry and PC-3M-luc-C6^*trpv6+/+*^-pTRPV6_wt_ stable clones (Supplementary Fig. 2w for mRNA and Supplementary Fig. 2x for protein levels). The same effects were observed for the VCaP^*trpv6+/+*^ (Supplementary Fig. 5e), LNCaP-C4-2B^*trpv6+/+*^ (Supplementary Fig. 5m), and LNCaP^*trpv6+/+*^ cell lines (Supplementary Fig. 5u). Since the expression of MMPs is not always a reliable factor, the enzymatic activity of MMP2 was analyzed in the conditioned media of PC-3M^*trpv6−/−*^-mCherry, PC-3M^*trpv6−/−*^-pTRPV6_wt_, and PC-3M^*trpv6−/−*^-pTRPV6^D582A^ stable cell clones. The data revealed a significant increase (*p* < 0.025) in MMP2 activity upon TRPV6 expression in the PC-3M^*trpv6−/−*^-pTRPV6_wt_ stable clone (Fig. 2u) as well as in the PC-3M-luc-C6^*trpv6+/+*^-pTRPV6_wt_ stable clone (*p* < 0.0001) (Supplementary Fig. 2y). Thus, our data provide strong evidence of the direct and crucial role of the TRPV6 channel in the migration and invasion, both basal and direct, of CRPC cells via the induction of EMT and increases in MMP expression and activity.

### TRPV6 mediates the increased phosphorylation of CaMK2 and NF-κB/RelA translocation and signaling

**Fig. 3:**
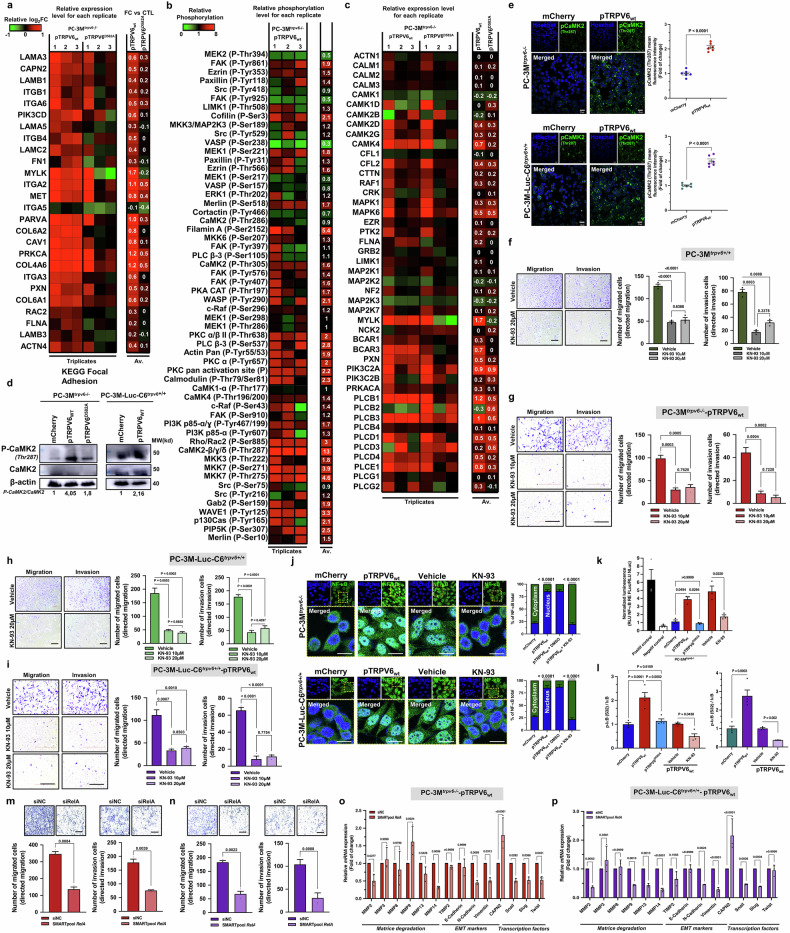
p-CaMK2 and NF-κB/RelA are involved in TRPV6-mediated signaling. **a** Heatmap of gene expression involved in basal migration via the focal adhesion pathway of the KEGG database with log_2_FC values in PC-3M^*trpv6−/−*^-pTRPV6_wt_ and PC-3M^*trpv6−/−*^-pTRPV6^D582A^ versus PC-3M^*trpv6−/−*^-mCherry stable cell clones. **b** Heatmap of key proteins involved in cytoskeleton phosphorylation with relative phosphorylation values in PC-3M^*trpv6−/−*^-pTRPV6_wt_ versus PC-3M^*trpv6−/−*^-mCherry stable cell clones. **c** Heatmap of gene expression shown in **b** with log_2_FC values in PC-3M^*trpv6−/−*^-pTRPV6_wt_ and PC-3M^*trpv6−/−*^-pTRPV6^D582A^ versus PC-3M^*trpv6−/−*^-mCherry stable cell clones. **d** Protein expression of p-CaMK2 (Thr287) and CaMK2 proteins in different PC-3M and PC-3M-luc-C6 cell clones with various channel expression levels. **e** p-CaMK2 (Thr287) staining in PC-3M^*trpv6−/−*^ and PC-3M-luc-C6^*trpv6+/+*^ prostate cancer cell lines and quantification of the mean intensity reported with Hoechst (*n* = 6). Scale bar, 20 μm. **f** Directed migration and invasion of PC-3M^*trpv6+/+*^ cells treated with the CaMK2 inhibitor KN-93 (10 or 20 μM) or vehicle. Representative images (left) and quantification of the number of cells that migrated and invaded through the matrix (right) (*n* = 4). Scale bar, 200 μm. **g** Directed migration and invasion of PC-3M^*trpv6−/−*^-pTRPV6_wt_ cells treated with the CaMK2 inhibitor KN-93 (10 or 20 μM) or vehicle. Representative images (left) and quantification of the number of cells that migrated and invaded through the matrix (right) (*n* = 4). Scale bar, 200 μm. **h** Directed migration and invasion of PC-3M-luc-C6^*trpv6+/+*^ cells treated with the CaMK2 inhibitor KN-93 (10 or 20 μM) or vehicle. Representative images (left) and quantification of the number of cells that migrated and invaded through the matrix (right) (*n* = 4). Scale bar, 200 μm. **i** Directed migration and invasion of PC-3M-luc-C6^*trpv6+/+*^ -pTRPV6_wt_ stable clone cells treated with the CaMK2 inhibitor KN-93 (10 or 20 μM) or vehicle. Representative images (left) and quantification of the number of cells that migrated and invaded through the matrix (right) (*n* = 4). Scale bar, 200 μm. **j** NF-κB/RelA staining of PC-3M^*trpv6−/−*^ and PC-3M-luc-C6^*trpv6+/+*^ cells with various TRPV6 expression levels treated with KN-93 (10 μM) or vehicle and quantification of nuclear-positive cells (*n* = 6). Scale bar, 20 µm. **k** Nuclear NF-κB protein activity in different PC-3M^*trpv6−/−*^ stable cell clones determined via a luciferase assay. **l** Detection of p-IκB (S32) and IκB proteins via ELISA in lysates of PC-3M^*trpv6−/−*^ and PC-3M-luc-C6^*trpv6+/+*^ cells with different channel expression levels as well as following treatment with the CaMK2 inhibitor KN-93 (10 μM), where the levels in the controls were normalized to 1. **m** Directed migration and invasion of PC-3M^*trpv6−/−*^ -pTRPV6_wt_ cell clones transfected with 40 nM SMARTpool against *relA* or a negative control (NC). Representative images and quantification of migrated and invasive cells (*n* = 4). Scale bar, 200 μm. **n** Directed migration and invasion of PC-3M-luc-C6^*trpv6+/+*^-pTRPV6_wt_ cell clones transfected with either 40 nM SMARTpool against *relA* or a negative control (NC). Representative images and quantification of migrated and invasive cells (*n* = 4). Scale bar, 200 μm. **o** Expression of genes involved in invasion and EMT in PC-3M^*trpv6-/*-^-pTRPV6_wt_ cells transfected with 40 nM SMARTpool against *relA* or a negative control (NC). **p** Expression of genes involved in invasion and EMT in PC-3M-luc-C6^*trpv6+/+*^-pTRPV6_wt_ cells transfected with either 40 nM SMARTpool against *relA* or a negative control (NC). Mean ± SEM (**e**–**p**). Two-sided *t* test (**e**, **j**–**p**). Two-way ANOVA (**f**–**i**, **k**, **l**). See also Supplementary Fig. 3

Transcriptomic analysis of PC-3M^*trpv6−/−*^-mCherry, PC-3M^*trpv6−/−*^-pTRPV6_wt_, and PC-3M^*trpv6−/−*^-pTRPV6^D582A^ stable cell clones revealed multiple differentially expressed genes (DEGs) between these clones (Supplementary Fig. 3a), highlighting the impact of calcium intake through the TRPV6 channel on the transcriptomic regulation of CRPC cells. First, the expression of genes involved in basal migration was studied via the focal adhesion pathway of the KEGG database (KEGG: 04510) (Fig. 3a). Notably, the most upregulated transcript was myosin light chain kinase (*mylk*), a serine/threonine-specific kinase known to promote invasion and migration. The key proteins involved in cytoskeleton phosphorylation with relative phosphorylation values in PC-3M^*trpv6−/−*^-pTRPV6_wt_ versus PC-3M^*trpv6−/−*^-mCherry stable cell clones were detected via a phosphorylation-specific antibody microarray, which revealed that the expression of functional TRPV6 increases the phosphorylation of FAK, cofilin, MEK1, filamin A, WASP, PKC, PLC, calmodulin, Rho, CaMK2, Gab2, WAVE1 and P130Cas without particularly increasing the transcriptomic expression of these same targets (Fig. 3b and c). The protein showing the greatest increase in the phosphorylation rate was Ca^2+^/calmodulin-dependent protein kinase II (CaMK2 pThr287), which was overexpressed in both the PC-3M^*trpv6−/−*^-pTRPV6_wt_ and PC-3M-luc-C6^*trpv6+/+*^-pTRPV6_wt_ stable cell clones (Fig. 3d and e), with no difference in the PC-3M^*trpv6−/−*^-pTRPV6^D582A^ stable cell clone (Supplementary Fig. 3c). The competitive and selective inhibitor of CaMK2 KN-93 was used to decrease its autophosphorylation rate (Supplementary Fig. 3b). KN-93 significantly inhibited both the migratory and invasive capacity of PC-3M^*trpv6+/+*^, PC-3M^*trpv6−/−*^-pTRPV6_wt_, PC-3 M-luc-C6^*trpv6+/+*^ and PC-3M-luc-C6^*trpv6+/+*^-pTRPV6_wt_ cells in both the Transwell® (all *p* < 0.001) (Fig. 3f–i) and wound-healing (Supplementary Fig. 3d and e) assays, with no effects on either the PC-3M^*trpv6−/−*^-mCherry or PC-3M^*trpv6−/−*^-pTRPV6^D582A^ stable cell clones (Fig. S3f and S3g). Identical data were obtained for both the directed migration and invasion transwell® assays for these clones (Supplementary Fig. 3h and i). The same effects were observed for the VCaP (Supplementary Fig. 5f), LNCaP-C4-2B (Supplementary Fig. 5n), and LNCaP cell lines (Supplementary Fig. 5v). With respect to the nonprostatic cell line HAP-1, 20 µM KN-93 significantly decreased both direct migration (*p* = 0.0014) and invasion (*p* = 0.0005) in HAP-1^*trpv6−/−*^-pTRPV6_wt_ cell clones (Supplementary Fig. 4o and p).

Furthermore, the NF-κB/*RelA* signaling pathway was studied to determine whether it is involved in both the migratory and invasive potential of PC-3M^*trpv6−/−*^ and PC-3M-luc-C6^*trpv6+/+*^-derived stable cell clones. KN-93 (10 μM) was used to decipher whether CaMK2 was involved upstream. The data clearly revealed that the functional form of the TRPV6 channel (in contrast to its pore mutant, TRPV6^D582A^ (Supplementary Fig. 3j)) is required for a significant increase in both the nuclear translocation of NF-κB (Fig. 3j) and its increased transcriptional activity (Fig. 3k). Moreover, a significant increase in the phosphorylation of p-IκB (S32) and IκB proteins was detected via ELISA in lysates of PC-3M^*trpv6−/−*^- and PC-3M-luc-C6^*trpv6+/+*^-derived cell clones, and the subsequent inhibition of CamK2 with 10 μM KN-93 was demonstrated (Fig. 3l), highlighting the essential role of CaMK2 in the nuclear translocation of NF-κB following calcium entry through the TRPV6 channel.

Finally, to study the potential role of NF-κB in the migratory and invasive behavior of CRPC cells expressing functional TRPV6 channels, we knocked down the *RelA* subunit (also known as p65, an REL-associated protein involved in NF-κB heterodimer formation, nuclear translocation and activation). The knockdown of *RelA* (please see *RelA* siRNA validation in Supplementary Fig. 3k, l and 4q, h, z) led to a significant decrease in the migratory and invasive potential (*p* = 0.0084 and *p* = 0.0039, respectively) of PC-3M^*trpv6−/−*^ -pTRPV6_wt_, PC-3M-luc-C6^*trpv6+/+*^-pTRPV6_wt_ (*p* = 0.0022 and *p* = 0.0088, respectively), HAP-1^*trpv6−/−*^-pTRPV6_wt_ (*p* = 0.0048 and *p* = 0.0011, respectively) cell clones, VCaP^*trpv6+/+*^ treated with siTRPV6, LNCaP-C4-2B^*trpv6+/+*^-mCherry and LNCaP-C4-2B^*trpv6+/+*^-pTRPV6_wt_, LNCaP^*trpv6+/+*^ treated with siTRPV6 (Fig. 3m and n, Supplementary Figs. 4r, s, 5h, p, x).

A large panel of genes associated with EMT and matrix degradation was studied under *RelA* knockdown. The data clearly revealed a significant decrease in MMP2 expression in both the PC-3M^*trpv6−/−*^-pTRPV6_wt_ and PC-3M-luc-C6^*trpv6+/+*^-pTRPV6_wt_ stable cell clones (*p* = 0.027 and *p* = 0.0002, respectively) (Fig. 3o and p). The level of MMP14 was also decreased in both cell clones (*p* = 0.0006 and *p* < 0.0001), while in PC-3M-luc-C6^*trpv6+/+*^-pTRPV6_wt_ cell clones, the levels of MMP9 and MMP13 were significantly lower (*p* = 0.0015 and *p* = 0.0013) than those in PC-3M^*trpv6−/−*^-pTRPV6_wt_. Both N-cadherin and vimentin were affected by *RelA* knockdown in both cell lines, whereas CAPN2 was significantly increased in both cell lines (*p* < 0.0001 for both). Although Snail, Slug, and Twist were significantly decreased in PC-3M^*trpv6−/−*^-pTRPV6_wt_ stable clones, the level of TWIST was unchanged in PC-3M-luc-C6^*trpv6+/+*^-pTRPV6_wt_ stable cell clones. Thus, these data suggest that the NF-κB/*RelA* signaling pathway is involved in CRPC aggressiveness downstream of the functional TRPV6 channel.

### The TRPV6 channel upregulates key markers of metastasis formation in vivo

**Fig. 4:**
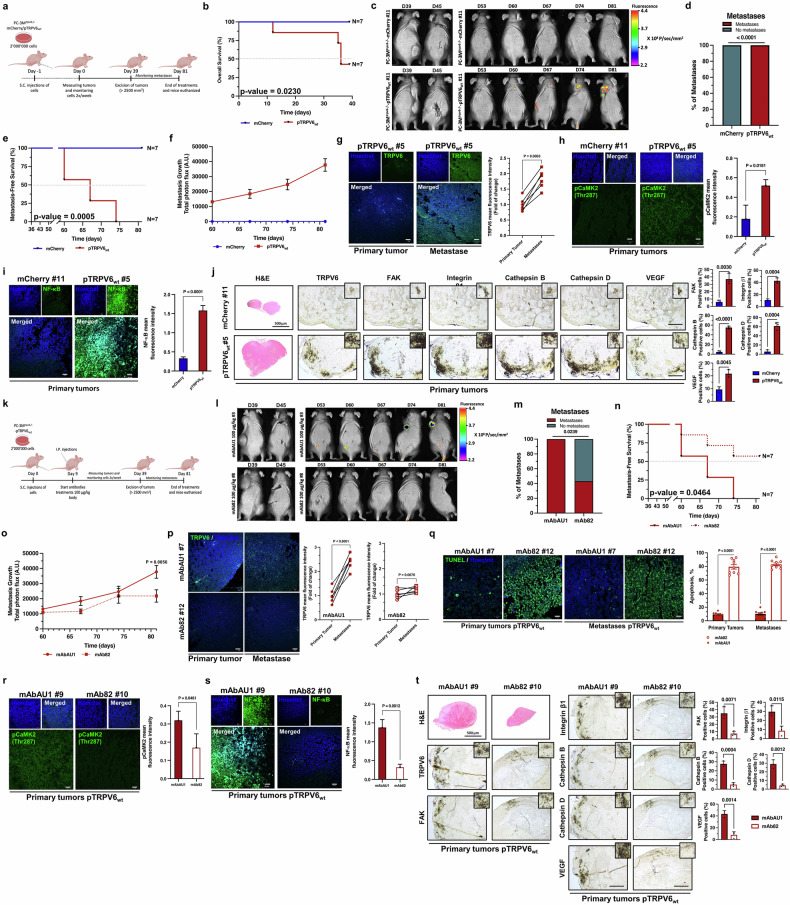
TRPV6 involvement in prostate cancer metastasis formation in vivo. **a** Timeline of the experimental design of the metastasis model in which PC-3M^*trpv6−/−*^-mCherry and PC-3M^*trpv6−/−*^-pTRPV6_*wt*_ stable clones (both expressing mCherry) were subcutaneously grafted into Swiss-nude mice. **b** Overall survival of mice bearing PC-3M^*trpv6−/−*^-mCherry (*n* = 7) and PC-3M^*trpv6−/−*^-pTRPV6_*wt*_ grafted cells (*n* = 7) before excision of the primary tumor via the log rank (Mantel‒Cox) test. **c** Representative images of mice bearing primary tumors before excision (left) and representative images of mCherry fluorescence time latches of emerging metastases under both conditions. **d** Percentages of metastasis incidence in PC-3M^*trpv6−/−*^-mCherry (*n* = 7)- and PC-3M^*trpv6−/−*^-pTRPV6_*wt*_ (*n* = 7)-grafted mice after excision of the primary tumor as described in (**c**)**. e** Metastasis-free survival of the PC-3M^*trpv6−/−*^-mCherry (*n* = 7) and PC-3M^*trpv6−/−*^-pTRPV6_*wt*_ (*n* = 7) groups was evaluated via the log-rank (Mantel‒Cox) test. **f** Metastatic growth following the excision of primary tumors from PC-3M^*trpv6−/−*^-mCherry (*n* = 7) and PC-3M^*trpv6−/−*^-pTRPV6_*wt*_ (*n* = 7) grafted cells. **g** Representative images and quantification of TRPV6 expression in primary tumor xenografts and corresponding nonbone metastases from the same mice bearing PC-3M^*trpv6−/−*^-pTRPV6_*wt*_ grafted cells (*n* = 6). Scale bars, 20 µm. **h** Representative images and quantification of p-CaMK2 (Thr287) in primary tumor xenografts from PC-3M^*trpv6−/−*^-mCherry and PC-3M^*trpv6−/−*^-pTRPV6_*wt*_ grafted mice. Scale bars, 20 µm. **i** Representative images and quantification of NF-κB/RelA in primary tumor xenografts from PC-3M^*trpv6−/−*^-mCherry and PC-3M^*trpv6−/−*^-pTRPV6_*wt*_ grafted mice. Scale bars, 20 µm. **j** Representative images and quantification of TRPV6, FAK, Integrin β1, cathepsin B, cathepsin D and VEGF expression in addition to H&E staining of primary tumor xenografts from mice bearing PC-3M^*trpv6−/−*^-mCherry (*n* = 7) and PC-3M^*trpv6-/-*^-pTRPV6_*wt*_ stable clones. Scale bar, 200 μm. **k** Timeline of the experimental design of the metastasis model in which PC-3M^*trpv6−/−*^-pTRPV6_*wt*_ stable clones expressing mCherry were subcutaneously grafted into Swiss-nude mice following intraperitoneal treatment with either 100 µg/kg mAbAU1 or mAb82. **l** Representative image of a mouse bearing primary tumors before excision (left) and representative mCherry fluorescence imaging time-lapse images of emerging metastases derived from PC-3M^*trpv6−/−*^-pTRPV6_*wt*_ grafted cells following treatment with either 100 µg/kg mAbAU1 or mAb82. **m** Percentage of metastasis incidence in the PC-3M^*trpv6−/−*^ pTRPV6_*wt*_ mouse group after excision of the primary tumor and following treatment with either 100 µg/kg mAbAU1 (*n* = 7) or mAb82 (*n* = 7). **n** Metastasis-free survival of PC-3M^*trpv6−/−*^-pTRPV6_*wt*_-grafted mice treated with either 100 µg/kg mAbAU1 (*n* = 7) or mAb82 (*n* = 7) determined via the log rank (Mantel‒Cox) test. **o** Metastatic growth after the excision of primary tumors derived from PC-3M^*trpv6−/−*^-pTRPV6_wt_ grafted mice treated with either 100 µg/kg mAbAU1 (*n* = 7) or mAb82 (*n* = 7). **p** Representative images and quantification of the TRPV6 channel in both primary tumor xenografts and corresponding metastases from the same mice bearing PC-3M^*trpv6−/−*^-pTRPV6_wt_ grafted cells and treated with either 100 µg/kg mAbAU1 (*n* = 7) or mAb82 (*n* = 7). Scale bars, 20 µm. **q** Representative staining and quantification of TUNEL-fluorescein-positive cells in primary tumor xenografts and their corresponding metastases from mice bearing PC-3M^*trpv6-/-*^-pTRPV6_wt_ cells and treated with either 100 µg/kg mAbAU1 (*n* = 3) or mAb82 (*n* = 3). Scale bars, 20 µm. **r** Representative images and quantification of p-CaMK2 (Thr287) in primary tumor xenografts and their corresponding metastases from mice bearing PC-3M^*trpv6−/−*^-pTRPV6_*wt*_ cells and treated with either 100 µg/kg mAbAU1 (*n* = 3) or mAb82 (*n* = 3). Scale bars, 20 µm. **s** Representative staining and quantification of NF-κB/RelA in primary tumor xenografts and their corresponding metastases from mice bearing PC-3M^*trpv6−/−*^-pTRPV6_*wt*_ cells and treated with either 100 µg/kg mAbAU1 (*n* = 3) or mAb82 (*n* = 3). Scale bars, 20 µm. **t** Representative staining and quantification of TRPV6, FAK, Integrin β1, cathepsin B, cathepsin D and VEGF in addition to H&E staining of primary tumor xenografts from mice bearing PC-3M^*trpv6−/−*^-pTRPV6_*wt*_ tumors treated with 100 μg/kg mAbAU1 or mAb82. Scale bar, 200 μm. Mean ± SEM (**f**–**j**, **o**–**t**). Two-tailed *t* test (**g**–**j**, **p**– **t**). Log rank (Mantel‒Cox) test (**b**, **e**, **n**)

To assess the ability of TRPV6 to promote metastasis formation in vivo, several experiments were carried out. The first protocol consisted of allowing tumor cells to grow and acquire natural invasive and migratory potential *via* EMT via PC-3M^*trpv6−/−*^-mCherry and PC-3M^*trpv6−/−*^-pTRPV6_wt_ stable clones grafted subcutaneously into Swiss-nude mice (Fig. 4a). The overall survival of the mice bearing the above grafted cells before excision of the primary tumor is shown in Fig. 4b and was significantly (*p* = 0.023) lower in the PC-3M^*trpv6−/−*^-pTRPV6_wt_ mice. Once the growing tumors reached the maximum allowed by the Ethical Committee size (=2500 mm^3^), they were safely excised, and the mice were monitored weekly for the presence of the mCherry signal. Representative images of mice bearing primary tumors before excision as well as a time-lapse image of emerging metastasis (mCherry fluorescence) under both conditions are shown in Fig. 4c. Strikingly, no metastasis developed in the mice bearing PC-3M^*trpv6−/−*^-mCherry clones, whereas 100% of the PC-3M^*trpv6−/−*^-pTRPV6_wt_ clones exhibited metastasis (*p* < 0.0001) (Fig. 4d). The metastasis-free survival of the PC-3M^*trpv6−/−*^-mCherry group versus the PC-3M^*trpv6−/−*^-pTRPV6_wt_ group is shown in Fig. 4e and was significantly (*p* = 0.0005) greater in the PC-3M^*trpv6−/−*^-mCherry group. The growth of metastases following the excision of primary tumors in both groups is shown in Fig. 4f. The primary tumors excised from mice bearing PC-3M^*trpv6−/−*^-pTRPV6_wt_ grafted cells were analyzed and compared to the metastases that developed from the same mouse (Fig. 4g). Representative immunofluorescence images of nonbone metastases from the same mouse as well as quantification of TRPV6 channels (Fig. 4g), pCamK2 (Thr287) (Fig. 4h), and NF-κB (Fig. 4i) revealed significant increases (*p* = 0.0003, *p* = 0.02, and *p* < 0.0001, respectively) in the expression/function of these key proteins in vivo. Similarly, IHC staining of TRPV6, FAK, Integrin β1, cathepsin B, cathepsin D and VEGF in primary tumor xenografts confirmed the high expression of these proteins in the PC-3M^*trpv6−/−*^-pTRPV6_wt_-grafted clones compared with the PC-3M^*trpv6−/−*^-mCherry clones (Fig. 4j and Supplementary Fig. 2z).

Recently, published anti-TRPV6 mAb, mAb82, was used to counter the effects of TRPV6 expression on metastasis formation.^28^ Once the PC-3M^*trpv6−/−*^-pTRPV6_wt_-grafted cells started forming palpable tumors, the mice were treated intraperitoneally with 100 µg/kg mAb82 twice per week, and the appearance of metastasis following primary tumor excision was monitored weekly (Fig. 4k). Representative images of mice bearing primary tumors before excision as well as a time-lapse of emerging metastasis (mCherry fluorescence) in mice treated with either 100 µg/kg mAbAU1 or mAb82 are shown in Fig. 4l. The occurrence of metastasis was decreased by almost 60% (*p* = 0.024) in the group treated with mAb82 compared with the isotype control mAbAU1 (Fig. 4m). The metastasis-free survival of PC-3M^*trpv6−/−*^-pTRPV6_wt_-grafted mice treated with either 100 µg/kg mAbAU1 or mAb82 was significantly greater (*p* = 0.046) in the mAb82-treated group (Fig. 4n). Metastasis growth after the excision of primary tumors derived from PC-3M^*trpv6−/−*^-pTRPV6_wt_-grafted mice treated with either 100 µg/kg mAbAU1 or mAb82 is shown in Fig. 4o. A significant increase (*p* < 0.0001) in TRPV6 channel expression was observed in the corresponding metastases from the same mice bearing PC-3M^*trpv6−/−*^-pTRPV6_wt_ grafted cells and treated with 100 µg/kg mAbAU1 or mAb82 (Fig. 4p). This increase was accompanied by enhanced TUNEL-fluorescein staining, indicating a significantly greater (*p* < 0.0001) apoptosis rate in the mice treated with 100 µg/kg mAb82 (Fig. 4q). Intriguingly, the levels of both p-CaMK2 (Thr287) and NF-κB/RelA were significantly lower (*p* < 0.046 and *p* < 0.0012, respectively) in primary tumors derived from mice treated with 100 µg/kg mAb82 than in those derived from mice treated with mAbAU1 (Fig. 4r, s). Finally, treatment of mice bearing PC-3M^*trpv6−/−*^-pTRPV6_wt_ grafted cells with 100 µg/kg mAb82 significantly decreased the expression levels of FAK, Integrin β1, Cathepsin B, Cathepsin D and VEGF (Fig. 4t). Thus, the TRPV6 channel is required for metastasis development in vivo via the upregulation of key markers of tumor aggressiveness, such as FAK, Integrin β1, Cathepsin B, Cathepsin D and VEGF.

### Functional coupling of the TRPV6 channel with the CXCR4 receptor is essential for bone-targeted metastasis

**Fig. 5:**
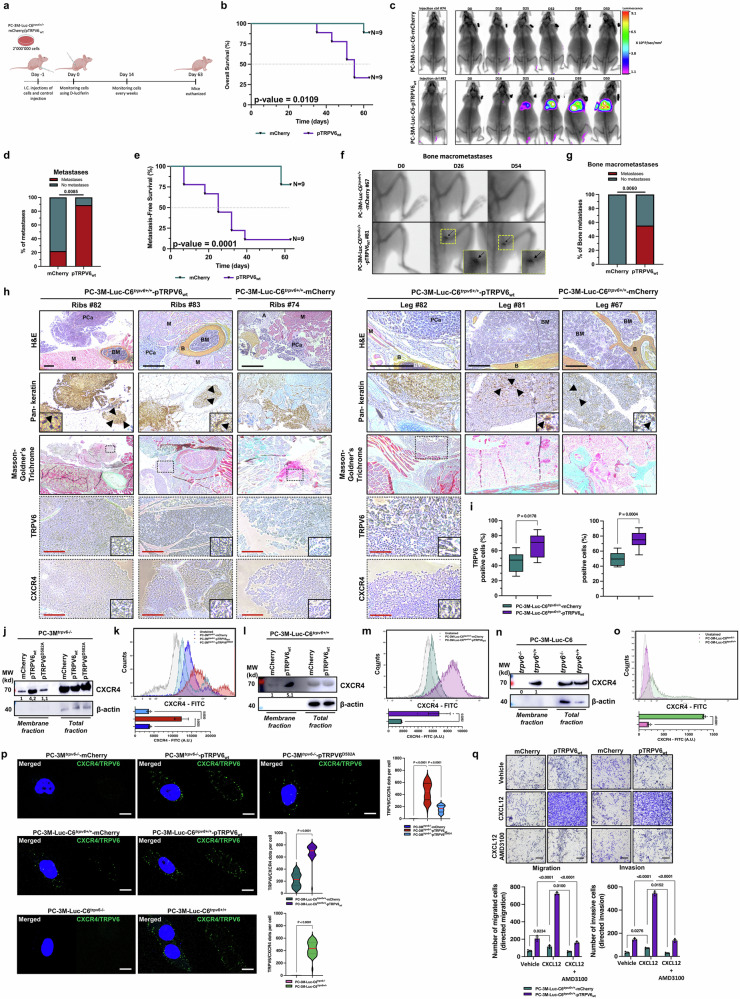
TRPV6 is directly involved in bone metastasis *by coupling* with the CXCR4 receptor. **a** Timeline of the experimental design of the bone metastasis model using Swiss-nude mice bearing either PC-3M-luc-C6^*trpv6+/+*^-mCherry or PC-3M-luc-C6^*trpv6+/+*^-pTRPV6_wt_ cell clones stably expressing firefly luciferase. I.C.: intracardiac. **b** Overall survival of PC-3M-luc-C6^*trpv6+/+*^-mCherry (*n* = 9) versus PC-3M-luc-C6^*trpv6+/+*^-pTRPV6_wt_ (*n* = 9) mice via the log rank (Mantel‒Cox) test. **c** Representative bioluminescence and X-ray images of mice prior to injection (left) and representative bioluminescence images and X-ray time latches of emerging bone metastases derived from PC-3M-luc-C6^*trpv6+/+*^-mCherry (*n* = 9) versus PC-3M-luc-C6^*trpv6+/+*^ -pTRPV6_wt_ (*n* = 9) cell clones grafted via intracardiac injection. **d** Percentage of metastasis incidence in mice bearing PC-3M-luc-C6^*trpv6+/+*^-mCherry (*n* = 9) versus PC-3M-luc-C6^*trpv6+/+*^-pTRPV6_*wt*_ (*n* = 9) grafted cells. **e** Comparison of the metastasis-free survival of PC-3M-luc-C6^*trpv6+/+*^-mCherry (*n* = 9) and PC-3M-luc-C6^*trpv6+/+*^-pTRPV6_*wt*_ (*n* = 9) grafted mice via the log rank (Mantel‒Cox) test. **f** Representative X-ray image of mouse bone macrometastasis (black arrows) following intracardiac injection of PC-3M-luc-C6^*trpv6+/+*^-mCherry or PC-3M-luc-C6^*trpv6+/+*^-pTRPV6_*wt*_ cell clones. **g** Incidence of bone macrometastasis in mice grafted via intracardiac injections of either PC-3M-luc-C6^*trpv6+/+*^-mCherry (*n* = 9) or PC-3M-luc-C6^*trpv6+/+*^-pTRPV6_wt_ (*n* = 9) cell clones, as revealed via X-ray analysis or surgery. **h** Representative staining of TRPV6, CXCR4, and pankeratin as well as histological staining by H&E and Masson–Goldner’s trichrome in bone metastases (black triangles) from both the ribs and legs derived from intracardiac injections of either PC-3M-luc-C6^*trpv6+/+*^-mCherry or PC-3M-luc-C6^*trpv6+/+*^-pTRPV6_wt_ cell clones. The dotted rectangle represents the area used for zooming in on TRPV6 and CXCR4 staining. M: muscle. PCa: prostate cancer. B: bone. BM: bone marrow. A: Adipocytes. Black scale bars, 100 μm; red scale bars, 50 μm. **i** Quantification of both TRPV6- and CXCR4-positive cells involved in bone metastasis was performed following intracardiac injections of either PC-3 M-luc-C6^*trpv6+/+*^-mCherry or PC-3M-luc-C6^*trpv6+/+*^-pTRPV6_wt_ cell clones. **j** Membrane and total protein expression of CXCR4 in PC-3M^*trpv6−/−*^-mCherry, PC-3M^*trpv6−/−*^-pTRPV6_wt_, and PC-3M^*trpv6−/−*^-pTRPV6^D582A^ stable cell clones. **k** Flow cytometric analysis of membrane CXCR4 expression in PC-3M^*trpv6−/−*^-mCherry, PC-3M^*trpv6−/−*^-pTRPV6_wt_, and PC-3M^*trpv6−/−*^-pTRPV6^D582A^ stable cell clones. **l** Membrane and total protein expression of CXCR4 in PC-3M-luc-C6^*trpv6+/+*^-mCherry versus PC-3M-luc-C6^*trpv6+/+*^-pTRPV6_*wt*_ cell clones. **m** Flow cytometric analysis of membrane CXCR4 expression in PC-3M-luc-C6^*trpv6+/+*^-mCherry versus PC-3M-luc-C6^*trpv6+/+*^ -pTRPV6_wt_ cell clones. **n** Membrane and total protein expression of CXCR4 in PC-3M-luc-C6^*trpv6-/-*^ versus PC-3M-luc-C6^*trpv6+/+*^ cells. **o** Flow cytometric analysis of membrane CXCR4 expression in PC-3M-luc-C6^*trpv6-/-*^ versus PC-3M-luc-C6^*trpv6+/+*^ cells. **p** Representative images and quantification (on the right) of TRPV6/CXCR4 coupling via the PLA in PC-3M^*trpv6−/−*^-mCherry, PC-3M^*trpv6−/−*^-pTRPV6_wt_, and PC-3M^*trpv6−/−*^-pTRPV6^D582A^ stable cell clones; PC-3M-luc-C6^*trpv6+/+*^-mCherry versus PC-3M-luc-C6^*trpv6+/+*^-pTRPV6_wt_ cell clones; and PC-3M-luc-C6^*trpv6−/−*^ versus PC-3M-luc-C6^*trpv6+/+*^ cells. Scale bars, 10 μm. **q** Directed migration and invasion of PC-3M-luc-C6^*trpv6+/+*^-mCherry versus PC-3M-luc-C6^*trpv6+/+*^-pTRPV6_wt_ cell clones treated with the specific CXCR4 inhibitor AMD3100 (30 μM) or vehicle to study the impact of CXCL12 as a chemoattractant (100 ng/mL). Representative images (left) and quantification of the number of cells that migrated and invaded through the matrix (right) (*n* = 3). Scale bar, 200 μm. Mean ± SEM (**i**, **k**, **m**, **o–q**). Two-tailed *t* test (**d**, **g**, **i**, **m**, **o, p**). Two-way ANOVA (**k**, **q**). Log rank (Mantel‒Cox) test (**b**, **e**). See also Supplementary Fig. 6

Intracardiac (i.c.) injections of PC-3M-luc-C6^*trpv6+/+*^-derived cell clones were used to generate bone metastases in Swiss nude mice (Fig. 5a). Both PC-3M-luc-C6^*trpv6+/+*^-mCherry and PC-3M-luc-C6^*trpv6+/+*^-pTRPV6_wt_ cell clones stably expressing firefly luciferase were used to study the role of TRPV6 expression in bone metastasis formation in vivo (Supplementary Fig. 6a and b). The overall survival of PC-3M-luc-C6^*trpv6+/+*^-mCherry-grafted mice compared with that of PC-3M-luc-C6^*trpv6+/+*^-pTRPV6_wt_ clones was significantly lower (*p* < 0.011) in mice grafted with PC-3M-luc-C6^*trpv6+/+*^-pTRPV6_wt_ clones (Fig. 5b). As such, bone metastasis was significantly enhanced (*p* = 0.0085) and correlated with increased TRPV6 channel expression (Fig. 5c and d). In addition, the metastasis-free survival of PC-3M-luc-C6^*trpv6+/+*^-pTRPV6_wt_-grafted mice was extremely low (*p* = 0.0001) compared with that of parental PC-3M-luc-C6^*trpv6+/+*^-mCherry-grafted mice (Fig. 5e). Representative X-ray images of mouse bone macrometastases following i.c. injection of PC-3M-luc-C6^*trpv6+/+*^-mCherry or PC-3M-luc-C6^*trpv6+/+*^-pTRPV6_wt_ cell clones are depicted in Fig. 5f. To ensure that the obtained bioluminescence signal was due to bone metastasis and not injection failure into the heart (e.g., physical retention of tumor cells in the lung), additional H&E control was performed, which revealed that no metastasis occurred in either the heart or the lungs of the mice (Supplementary Fig. 6c). The level of TRPV6, the day of i.c. injection and the date of metastasis in vivo were monitored and are shown in Supplementary Fig. 6d. The degree of bone macrometastasis incidence in the mice grafted via i.c. injections was predictably greater (p = 0.006) than that in the PC-3M-luc-C6^*trpv6+/+*^-pTRPV6_wt_-grafted cell clones (Fig. 5g). Further IHC analysis of TRPV6, CXCR4, and pankeratin staining as well as histological staining via H&E and Masson–Goldner’s trichrome staining of bone metastases derived from both the ribs and legs suggested a direct correlation and likely colocalization of the TRPV6 channel with the CXCR4 receptor (Fig. 5h). Indeed, the chemokine receptor CXCR4 is a promising candidate because of its known involvement in the PIP2/PLC signaling pathway, which regulates ER calcium stores and might thereby influence the activation of calcium channels such as TRPV6.^36,37^ The quantification of both TRPV6- and CXCR4-positive cells in bone metastases generated from mice grafted with either PC-3M-luc-C6^*trpv6+/+*^-mCherry or PC-3M-luc-C6^*trpv6+/+*^-pTRPV6_wt_ cell clones confirmed the initial hypothesis (Fig. 5i). Bone metastases generated from PC-3M-luc-C6^*trpv6+/+*^-pTRPV6_wt_ cell clones presented significantly greater numbers of both TRPV6- and CXCR4-positive cells (*p* = 0.018 and *p* = 0.0004, respectively). This greater level of CXCR4 expression in PC-3M-luc-C6^*trpv6+/+*^-pTRPV6_wt_ cell clone-generated bone metastases led to the investigation of CXCR4 expression in the aforementioned PC-3M^*trpv6−/−*^-based clones (Fig. 5j). Although the total protein expression of the CXCR4 receptor (as well as its mRNA expression, Supplementary Fig. 6e) was unchanged, its membrane fraction was likely increased in the corresponding membrane fraction (Fig. 5j). Flow cytometric analysis of membrane CXCR4 expression in PC-3M^*trpv6−/−*^-mCherry, PC-3M^*trpv6−/−*^-pTRPV6_wt_, and PC-3M^*trpv6−/−*^-pTRPV6^D582A^ stable cell clones revealed a significant (*p* = 0.003) increase in the CXCR4 receptor on the plasma membrane upon TRPV6 expression (Fig. 5k). Similarly, the PC-3M-luc-C6^*trpv6+/+*^-pTRPV6_wt_ clones expressed the CXCR4 receptor predominantly (*p* = 0.026) on the cell surface (Figs. 5l and m) and not at the level of total protein or mRNA (Supplementary Fig. 6f), whereas the PC-3M-luc-C6^*trpv6−/−*^ clones contained no CXCR4 receptor (*p* < 0.0001) on the plasma membrane (being equally expressed at the level of both total protein and mRNA (Supplementary Fig. 6g), irrespective of its total expression (Fig. 5n and o).

Full-scale analysis of TRPV6/CXCR4 coupling via the PLA was performed in PC-3M^*trpv6−/−*^-mCherry, PC-3M^*trpv6−/−*^-pTRPV6_wt_, and PC-3M^*trpv6−/−*^-pTRPV6^D582A^ stable cell clones; PC-3M-luc-C6^*trpv6+/+*^-mCherry versus PC-3M-luc-C6^*trpv6+/+*^-pTRPV6 cell clones; and PC-3M-luc-C6^*trpv6−/−*^ versus PC-3M-luc-C6^*trpv6+/+*^ cells (Fig. 5p), revealing significant coupling between TRPV6 and CXCR4 in all the cell clones studied where the TRPV6 channel was expressed or reintroduced (*p* < 0.0001 for all the studied cell type clones). To determine whether CXCR4/TRPV6 coupling was functional, 100 ng/mL CXCL12, a chemoattractant for CXCR4, was used to demonstrate the considerable (*p* < 0.0001 in all cases) enhancement of PC-3M-luc-C6^*trpv6+/+*^-mCherry versus PC-3M-luc-C6^*trpv6+/+*^-pTRPV6_wt_ cell clone migration as well as invasion (Fig. 5q). Intriguingly, the CXCL12 ligand had a very small effect on both directed migration and invasion (55% ± 1.5 (*p* = 0.055) versus 67% ± 11 (*p* = 0.0017), respectively) in PC-3M-luc-C6^*trpv6−/−*^ cells compared with PC-3M-luc-C6^*trpv6+/+*^ cells (160% ± 17 (*p* < 0.0001) versus 139% ± 16 (*p* < 0.0001), respectively) (Supplementary Fig. 6h and i). In contrast, the specific CXCR4 antagonist AMD3100^38^ successfully inhibited the CXCL12-induced effects (Fig. 5q). While coimmunoprecipitating TRPV6 and CXCR4 in PC-3M-luc-C6^*trpv6+/+*^-mCherry versus PC-3M-luc-C6^*trpv6+/+*^-pTRPV6_wt_ clones, the formation of CXCR4/TRPV6 complexes was positively correlated with TRPV6 levels (Supplementary Fig. 6j).

Furthermore, intracardiac injections of PC-3M-luc-C6^*trpv6−/−*^ cells or PC-3M-luc-C6^*trpv6+/+*^ cells expressing firefly luciferase (Supplementary Fig. 6k and l) were used to study bone metastasis generation when the *trpv6* gene was knocked out (Supplementary Fig. 6m). The overall survival of PC-3M-luc-C6^*trpv6−/−*^-grafted mice was significantly greater than that of PC-3M-luc-C6^*trpv6+/+*^-grafted mice (*p* = 0.017) (Supplementary Fig. 6n). A representative time-lapse image of bone emergence via X-ray and bioluminescence imaging following intracardiac injection of PC-3M-luc-C6^*trpv6−/−*^ or PC-3M-luc-C6^*trpv6+/+*^ cells is depicted in Supplementary Fig. 6o. Metastasis occurrence was decreased by 68% (*p* = 0.0087) in PC-3M-luc-C6^*trpv6−/−*^-grafted mice compared with PC-3M-luc-C6^*trp6+/+*^-grafted mice (Supplementary Fig. 6p). Metastasis-free survival was significantly greater (*p* = 0.0461) in PC-3M-luc-C6^*trpv6−/−*^-grafted mice than in PC-3M-luc-C6^*trp6+/+*^-grafted mice (Supplementary Fig. 6q). Representative 3D images of mouse rib metastasis from different angles of view in mice bearing PC-3M-luc-C6^*trpv6−/−*^ versus PC-3M-luc-C6^*trp6+/+*^-grafted cells are depicted in Supplementary Fig. 6r. Thus, a functional TRPV6/CXCR4 complex on the plasma membrane of cancer cells is formed and promotes bone metastasis, while total CXCR4 receptor expression is not affected.

### TRPV6 expression affects bone architecture and metastasis development in vivo

**Fig. 6:**
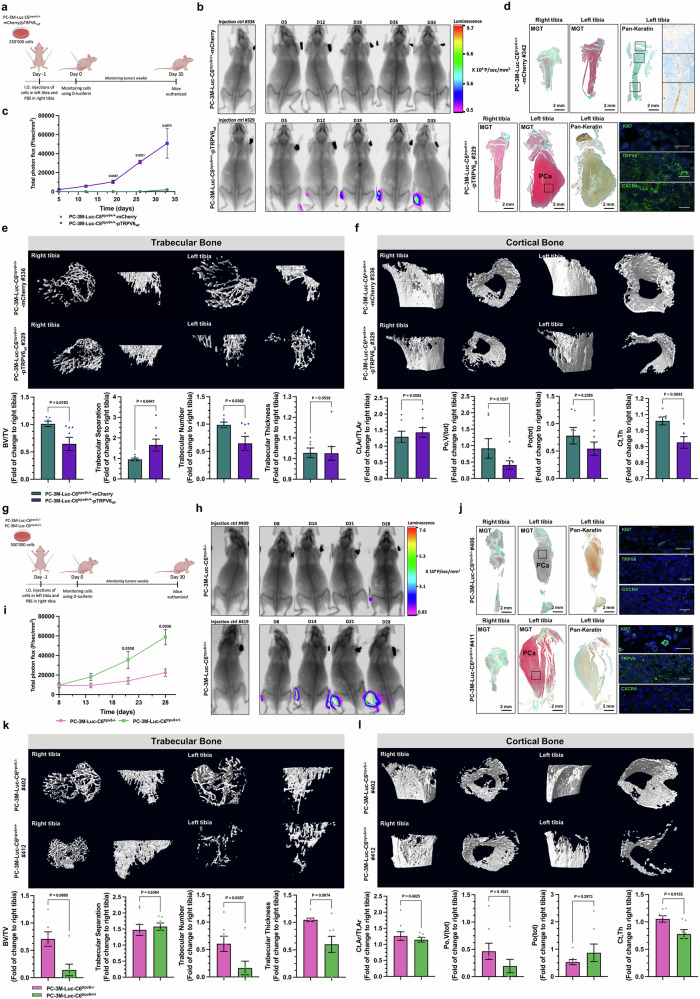
Effect of TRPV6 expression on bone metastasis development and bone architecture in vivo. **a** Timeline of the experimental bone-homing model using PC-3M-luc-C6^*trpv6+/+*^-mCherry and PC-3M-luc-C6^*trpv6+/+*^-pTRPV6_wt_ cell clones stably expressing firefly luciferase in Swiss-nude mice. I.O.: intraosseous. **b** Representative bioluminescence images and X-rays of mice bearing metastases from grafted i.o. PC-3M-luc-C6^*trpv6+/+*^-mCherry and PC-3M-luc-C6^*trpv6+/+*^-pTRPV6_wt_ cell clones. **c** Bone metastasis growth expressed as total photon flux in mice bearing intraosseous xenografts of PC-3M-luc-C6^*trpv6+/+*^ -mCherry (*n* = 10) and PC-3M-luc-C6^*trpv6+/+*^ -pTRPV6_wt_ (*n* = 10). **d** Representative images of Masson-Goldner’s trichrome, pankeratin, Ki67, TRPV6 and CXCR4 staining in tibias from mice bearing intraosseous xenografts of PC-3M-luc-C6^*trpv6+/+*^-mCherry and PC-3M-luc-C6^*trpv6+/+*^-pTRPV6_wt_. Black scale bar, 2 mm. White scale bars, 20 μm. **e** and **f** 3D images of both the trabecular (**e**) and cortical (**f**) bone microarchitectures of the tibia and the BV/TV ratio, trabecular separation, trabecular number and trabecular thickness, cortical/total cross-sectional area, cortical thickness, total volume of pores, and total porosity expressed over the right tibia (control injection of PBS) of mice bearing intraosseous xenografts of PC-3M-luc-C6^*trpv6+/+*^-mCherry and PC-3M-luc-*C6*^*trp6+/+*^-pTRPV6_wt_ cell clones. **g** Timeline of the experimental bone-homing model using PC-3M-luc-C6^*trpv6−/−*^ and PC-3M-luc-C6^*trpv6+/+*^ cell clones stably expressing firefly luciferase in Swiss-nude mice. I.O.: intraosseous. **h** Representative bioluminescence images and X-rays of mice bearing metastases derived from intraosseous xenografts of PC-3M-luc-C6^*trpv6−/−*^ and PC-3 M-luc-C6^*trpv6+/+*^ cell clones. **i** Bone metastasis growth expressed as total photon flux in mice bearing intraosseous xenografts of PC-3M-luc-C6^*trpv6−/−*^ (*n* = 10) and PC-3M-luc-C6^*trpv6+/+*^ (*n* = 10) mice. **j** Representative Masson–Goldner’s trichrome, pankeratin, Ki67, TRPV6 and CXCR4 staining of tibias from mice bearing intraosseous xenografts of PC-3M-luc-C6^*trpv6−/−*^ and PC-3M-luc-C6^*trpv6+/+*^ cell clones. Black scale bars, 2 mm. White scale bars, 20 μm. **k** and **l** 3D images of both the trabecular (**k**) and cortical (**l**) bone microarchitectures at the tibia and the BV/TV ratio, trabecular separation, trabecular number and trabecular thickness, cortical/total cross-sectional area ratio, cortical thickness, total volume of pores, and total porosity expressed over the right tibia (control injection of PBS) of mice bearing intraosseous xenografts of PC-3M-luc-C6^*trpv6−/−*^ or PC-3M-luc-C6^*trpv6+/+*^ cells. Mean ± SEM (**c**, **e**, **f**, **i**, **k**, **l**). Two-sided *t* test (**c**, **e**, **f**, **i**, **k**, **l**). See also Supplementary Fig. 7

An experimental bone-homing model using PC-3M-luc-C6^*trpv6+/+*^-mCherry and PC-3M-luc-C6^*trpv6+/+*^-pTRPV6_wt_ cell clones stably expressing firefly luciferase was used to study the role of the TRPV6 channel in the metastatic niche (Fig. 6a and Supplementary Fig. 7a). Either PC-3M-luc-C6^*trpv6+/+*^-mCherry or PC-3M-luc-C6^*trpv6+/+*^-pTRPV6_wt_ cell clones were injected into the left tibia, with the concomitant injection of PBS into the right tibia. Representative bioluminescence images and X-rays of mice bearing metastases from grafted i.o. PC-3M-luc-C6^*trpv6+/+*^-mCherry and PC-3M-luc-C6^*trpv6+/+*^-pTRPV6_wt_ cell clones are shown in Fig. 6b. The growth of bone metastasis, expressed as total photon flux, was highly increased (*p* < 0.05 starting from day 18 after grafting) when PC-3M-luc-C6^*trpv6+/+*^-pTRPV6_wt_ cell clones were injected (Fig. 6c). Tibia from mice bearing intraosseous xenografts were excised and analyzed via Masson–Goldner’s Trichrome, as were antibodies against pankeratin, Ki67, TRPV6 and CXCR4 (Fig. 6d and Supplementary Fig. 7c for H&E staining). The pankeratin staining clearly revealed the epithelial origin of the osteoblastic tumors as well as the presence of the TRPV6 and CXCR4 proteins.

3D images of both the trabecular and cortical bone microarchitectures of the tibia were obtained (Fig. 6e and f). For each bone, the ratio of BV/TV, trabecular separation, trabecular number and trabecular thickness, expressed over the right tibia (control injection of PBS), was calculated. The ratio BV/TV, the ratio of the segmented bone volume to the total volume, was lower (*p* = 0.02) in PC-3M-luc-C6^*trpv6+/+*^-pTRPV6_*wt*_–grafted cell clones for the trabecular bone, whereas the mean distance between trabeculae was greater (*p* = 0.44). The trabecular number, a measure of the average number of trabeculae per unit length, was significantly (*p* = 0.04) lower in PC-3M-luc-C6^*trpv6+/+*^-pTRPV6_wt_-grafted cell clones than in PC-3M-luc-C6^*trpv6+/+*^-mCherry-grafted cell clones, although no difference (*p* = 0.96) was found in the trabecular thickness or the mean thickness of the trabeculae (Fig. 6e). For the cortical bone, no difference (*p* = 0.55) was found for the ratio Ct, Ar/Tt,Ar, or the cortical area fraction, whereas the Po, V(tot), total pore volume, and Po(tot) values and total porosity tended to decrease with no statistical significance (*p* = 0.124 and *p* = 0.24, respectively). In contrast, the Ct and Th values and cortical thickness were significantly lower in PC-3M-luc-C6^*trpv6+/+*^-pTRPV6_wt_-grafted cell clones (*p* = 0.009) (Fig. 6f).

Since no significant tumor growth was observed for the PC-3M-luc-C6^*trpv6+/+*^-mCherry-grafted i.o. cell clones, the quantity of cells was doubled, and the degree of bone homing of the PC-3M-luc-C6^*trpv6+/+*^ cells was comparable to that of the PC-3M-luc-C6^*trpv6−/−*^ cells in the identical in vivo protocol (Fig. 6g and Supplementary Fig. 7b). Either PC-3M-luc-C6^*trpv6+/+*^ or PC-3M-luc-C6^*trpv6−/−*^ cell clones were injected into the left tibia, with the concomitant injection of PBS into the right tibia. Representative bioluminescence images and X-rays of mice bearing metastases from grafted i.o. PC-3M-luc-C6^*trpv6+/+*^ or PC-3M-luc-C6^*trpv6−/−*^ cells are shown in Fig. 6h. The growth of bone metastasis, expressed as total photon flux, was significantly different (*p* < 0.036 starting from day 20 after grafting) in favor of PC-3M-luc-C6^*trpv6+/+*^ cells versus PC-3M-luc-C6^*trpv6−/−*^ cells (Fig. 6i). Tibia from mice bearing intraosseous xenografts were excised and analyzed via Masson–Goldner’s Trichrome, as were antibodies against pankeratin, Ki67, TRPV6 and CXCR4 (Fig. 6j and Supplementary Fig. 7c for H&E staining). Pankeratin staining confirmed the epithelial origin of the osteoblastic tumors, and the absence of TRPV6 and CXCR4 proteins was confirmed.

3D images of both the trabecular and cortical bone microarchitectures of the tibia were obtained (Fig. 6k and l). For the previous experiments, the ratios of BV/TV, trabecular separation, and trabecular number and trabecular thickness, expressed over the right tibia (control injection of PBS), were calculated. The BV/TV ratio was significantly lower (*p* = 0.006) in PC-3M-luc-C6^*trpv6+/+*^ cells than in control cells for trabecular bone, with no difference in trabecular separation (*p* = 0.64), whereas the trabecular number was significantly lower (*p* = 0.036) in the case of PC-3M-luc-C6^*trpv6+/+*^ cells grafted into the tibia (Fig. 6k). The same decrease was observed for the trabecular thickness of PC-3M-luc-C6^*trpv6+/+*^ cells compared with that of PC-3M-luc-C6^*trpv6−/−*^ cells (*p* = 0.007). For the cortical bone, no difference (*p* = 0.48) was found for the Ct, Ar/Tt, or Ar ratios or for the Po, V(tot) (*p* = 0.19), or Po(tot) values (*p* = 0.3). In contrast, the Ct,Th values were significantly lower in the PC-3M-luc-C6^*trpv6+/+*^ cells (*p* = 0.009) (Fig. 6l). Representative 3D reconstruction images of mouse tibia metastases derived from both PC-3M-luc-C6^*trpv6−/−*^ and PC-3M-luc-C6^*trpv6+/+*^ grafted cells are shown in Supplementary Fig. 7c. Thus, it can be concluded that TRPV6 expression affects bone architecture and metastasis development in vivo by lowering the BV/TV ratio and decreasing the trabecular number and the Po, V(tot) and Ct,Th values.

### TRPV6 targeting in vivo suppresses bone metastasis

**Fig. 7:**
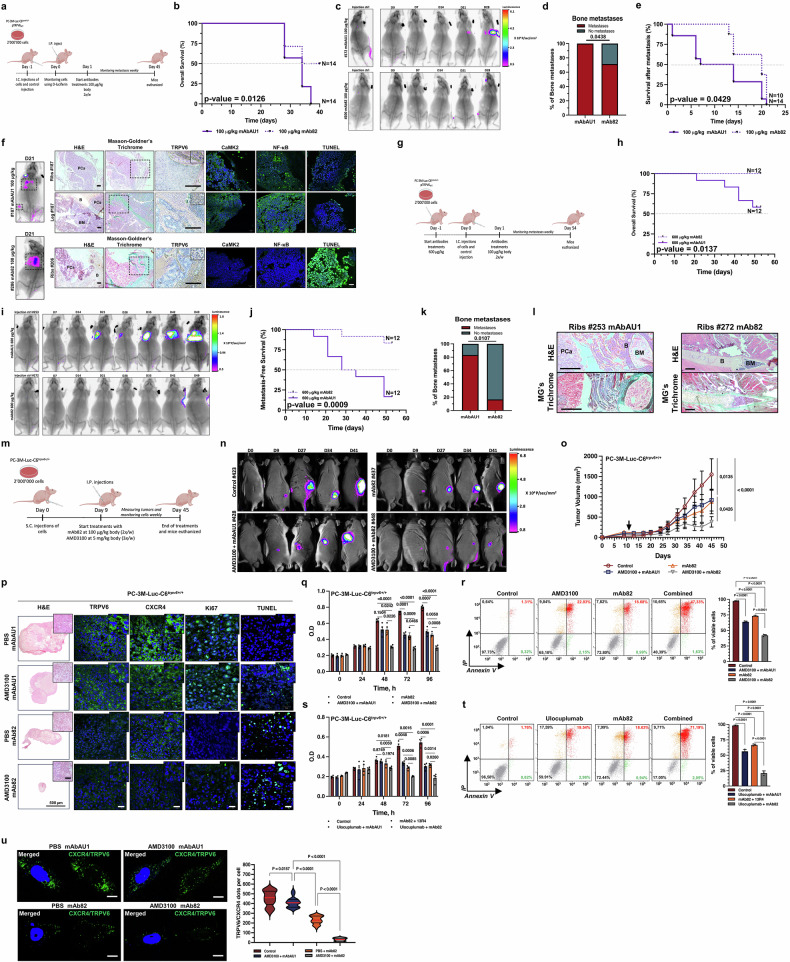
TRPV6 targeting via an anti-TRPV6 monoclonal antibody suppresses bone metastasis in vivo. **a** Timeline of the experimental bone metastasis model using Swiss mice grafted with PC-3M-luc-C6^*trpv6+/*^-pTRPV6_WT_ cell clones stably expressing firefly luciferase and treated with either 100 µg/kg mAbAU1 or mAb82 24 h before intracardiac cell injection, followed by treatment with either 100 µg/kg mAbAU1 or mAb82 twice per week starting 24 h after intracardiac cell injection. I.C.: intracardiac. **b** Overall survival of mice bearing PC-3M-luc-C6^*trpv6+/+*^ -pTRPV6_wt_ cell clones and treated with either 100 µg/kg mAbAU1 (*n* = 14) or mAb82 (*n* = 14) 24 h after intracardiac cell injection, as determined via the log rank (Mantel‒Cox) test. **c** Representative bioluminescence images and X-rays of mice grafted with PC-3M-luc-C6^*trpv6+/+*^-pTRPV6_wt_ cell clones and treated with either 100 µg/kg mAbAU1 (*n* = 14) or mAb82 (*n* = 14) 24 h after intracardiac cell injection. **d** Incidence of bone metastasis in mice grafted with PC-3M-luc-C6^*trpv6+/+*^-pTRPV6_wt_ cell clones and treated with either 100 µg/kg mAbAU1 (*n* = 14) or mAb82 (*n* = 14) 48 h after intracardiac cell injection. **e** Survival of PC-3M-luc-C6^*trpv6+/+*^ -pTRPV6_wt_ mice and mice treated with either 100 µg/kg mAbAU1 (*n* = 14) or mAb82 (*n* = 14) 24 h after intracardiac cell injection, as determined via the log rank (Mantel‒Cox) test. **f** Representative bioluminescence imaging and X-ray images of mice bearing metastases on the last day of treatment with either 100 µg/kg mAbAU1 or mAb82 (left) and representative H&E, Masson–Goldner’s trichrome and TRPV6 staining of bone metastases (right) from these mice. PCa: prostate cancer. B: bone. BM: bone marrow. Black scale bars, 100 µm. White scale bars, 20 µm. **g** Timeline of the experimental bone metastasis model using Swiss mice grafted with PC-3M-luc-C6^*trpv6+/+*^-pTRPV6_wt_ cell clones stably expressing firefly luciferase and treated with either 600 µg/kg mAbAU1 or mAb82 24 h before intracardiac cell injection, followed by treatment with either 100 µg/kg mAbAU1 or mAb82 twice per week starting 24 h after intracardiac cell injection. I.C.: intracardiac. **h** Overall survival of mice bearing PC-3 M-luc-C6^*trpv6+/+*^ -pTRPV6_wt_ cell clones and treated with either 600 µg/kg mAbAU1 (*n* = 12) or mAb82 (*n* = 12) 24 h before intracardiac cell injection and then treated with either 100 µg/kg mAbAU1 or mAb82 twice per week starting 24 h after intracardiac cell injection, as determined via the log rank (Mantel‒Cox) test. **i** Representative bioluminescence imaging and X-ray images of mice grafted with PC-3M-luc-C6^*trpv6+/+*^-pTRPV6_wt_ cell clones and treated with either 600 µg/kg mAbAU1 (*n* = 12) or mAb82 (*n* = 12) 24 h before intracardiac cell injection, followed by treatment with either 100 µg/kg mAbAU1 or mAb82 twice per week starting 24 h after intracardiac cell injection. **j** Metastasis-free survival of mice bearing PC-3M-luc-C6^*trpv6+/+*^-pTRPV6_wt_ and treated with either 600 µg/kg mAbAU1 (*n* = 12) or mAb82 (*n* = 12) 24 h before intracardiac cell injection and then treated with either 100 µg/kg mAbAU1 or mAb82 twice per week starting 24 h after intracardiac cell injection, as determined via the log rank (Mantel‒Cox) test. **k** Incidence of bone metastasis in mice bearing PC-3M-luc-C6^*trpv6+/+*^-pTRPV6_wt_ and treated with either 600 µg/kg mAbAU1 (*n* = 12) or mAb82 (*n* = 12) 24 h before intracardiac cell injection, as determined via the log rank (Mantel‒Cox) test. **l** Representative H&E and Masson–Goldner’s Trichrome staining of bone metastases derived from the ribs of mice treated with either mAbAU1- or mAb82-treated mice as described in (**a**). PCa: prostate cancer. B: bone. BM: bone marrow. Scale bars, 100 µm. **m** Timeline of the experimental tumor growth model using PC-3M-luc-C6^*trpv6+/+*^ cells stably expressing luciferase in Swiss-nude mice treated with either 100 µg/kg AU1 or mAb82 together with 5 mg/kg AMD3100 following tumor formation. S.C.: Subcutaneous. I.P.: Intraperitoneal. **n** Representative bioluminescence images of mice bearing xenografts of PC-3M-luc-C6^*trpv6+/+*^ cells treated with either 100 µg/kg mAbAU1 or mAb82 together with 5 mg/kg AMD3100 following tumor formation. **o** Tumor growth in mice bearing xenografts of PC-3M-luc-C6^*trpv6+/+*^ cells treated with either 100 µg/kg mAbAU1 or mAb82 together with 5 mg/kg AMD3100 following tumor formation. **p** Representative H&E, TRPV6, CXCR4, Ki-67 and TUNEL-fluorescein staining of primary tumors from mice bearing xenografts of PC-3M-luc-C6^*trpv6+/+*^ cells treated with either 100 µg/kg mAbAU1 or mAb82 together with 5 mg/kg AMD3100 following tumor formation. Scale bar, 20 µm. **q** Survival assay (MTS) of PC-3M-luc-C6^*trpv6+/+*^ cells treated with either 6 µg/mL mAbAU1/mAb82 together with 30 mM AMD3100 for 4 days. **r** Annexin V-FITC and IP analysis and quantification via flow cytometry of PC-3M-luc-C6^*trpv6+/+*^ cells treated with either 6 µg/mL mAbAU1/mAb82 or 30 mM AMD3100 for 3 days. **s** Survival assay (MTS) of PC-3M-luc-C6^*trpv6+/+*^ cells treated with either 6 µg/mL ulocuplumab or mAb82 or mAbAU1, as a control, for 4 days. **t** Annexin V-FITC and IP analysis and quantification via flow cytometry of PC-3M-luc-C6^*trpv6+/+*^ cells treated with either 6 µg/mL ulocuplumab or mAb82 or mAbAU1, as a control, for 3 days. **u** Representative images and quantification of TRPV6/CXCR4 complexes revealed via the PLA in PC-3M-luc-C6^*trpv6+/+*^ cells treated with either 6 µg/mL mAb82 together with 30 mM AMD3100 for 36 h. Scale bar, 10 µm. Mean ± SEM (**o**, **q– t**). Two-sided *t* test (**d**, **k**). Two-way ANOVA (**o**, **q–u**). Log rank (Mantel‒Cox) test (**b**, **e**, **h**, **k**). See also Supplementary Fig. 7

Different therapeutic options have been developed to study the role of the TRPV6 channel in both the ability of CRPC cells to target bone and their ability to home to bone tissue. First, CRPC cells, such as PC-3M-luc-C6^*trpv6+/+*^-pTRPV6_wt_ cell clones stably expressing firefly luciferase, were grafted via intracardiac injection, followed by 48 h before treatment with either 100 µg/kg mAbAU1 or mAb82 twice per week (Fig. 7a and Supplementary Fig. 7d). In this therapeutic model, the cells were allowed to reach the bone tissue and anchor. The overall survival of the mice was greater (*p* = 0.013) in the group of mice treated with 100 µg/kg mAb82 twice per week (Fig. 7b). Representative bioluminescence images and X-rays of mice bearing metastases from grafted i.c. PC-3M-luc-C6^*trpv6+/+*^-pTRPV6_wt_ cell clones are shown in Fig. 7c. The occurrence of metastasis was decreased by 28.6% (*p* = 0.044) in the group of mice treated with 100 µg/kg mAb82 (Fig. 7d). The survival of the mice after metastasis formation is shown in Fig. 7e and was still greater (*p* = 0.043) in the group treated with 100 µg/kg mAb82. Metastasis-free survival is depicted in Supplementary Fig. 7e. On the last day of the mAb treatments, both bioluminescence imaging and X-ray imaging of the mice bearing metastases, as well as representative H&E, Masson–Goldner’s trichrome, TRPV6, CaMK2, NF-κB and TUNEL staining of the bone metastases were performed (Fig. 7f), revealing lower levels of TRPV6, CaMK2, and NF-κB expression in the group treated with 100 µg/kg mAb82, as well as apoptosis-mediated cell death.

Since the detection of bone metastasis in patients is always a late event during disease, a model in which treatment with an anti-TRPV6 antibody precedes metastasis occurrence and thus interferes with metastasis initiation was studied (Supplementary Fig. 7f and g). As such, 100 µg/kg mAb82 was injected i.p. into the mice 24 h prior to the i.c. injection of PC-3M-luc-C6^*trpv6+/+*^-pTRPV6_wt_ cell clones, followed by treatment with either 100 µg/kg mAbAU1 or mAb82 twice per week. The overall survival of the mice in the group treated with 100 µg/kg mAb82 was significantly greater (*p* = 0.012) (Supplementary Fig. 7h). A representative time-lapse image of bone metastasis emergence via X-ray and bioluminescence imaging following the i.c. injection of PC-3M-luc-C6^*trpv6+/+*^-pTRPV6_wt_ cell clones and treatment with both mAbs is depicted in Supplementary Fig. 7i. Metastasis occurrence was significantly (*p* = 0.039) reduced by 37.1% in the group of mice treated with 100 µg/kg mAb82, as shown in Supplementary Fig. 7j. The metastasis-free survival of the above groups is shown in Supplementary Fig. 7k.

Since the efficiency in suppressing bone metastasis formation was greater when the first dose of 100 µg/kg mAb82 was injected 24 h prior to the i.c. injection of PC-3M-luc-C6^*trpv6+/+*^-pTRPV6_wt_ cell clones (Supplementary Fig. 7l), the hypothesis of the primary role of TRPV6 in metastasis anchoring and/or initiation was validated via high bolus i.p. injection of 600 µg/kg mAbAU1 and mAb82 24 h prior to the i.c. grafting of PC-3M-luc-C6^*trpv6+/+*^-pTRPV6_wt_ cell clones (Fig. 7g and Supplementary Fig. 7l). The overall survival of the mice in the group treated with 600 µg/kg mAb82 24 h prior to i.c. grafting was significantly (*p* = 0.014) greater (Fig. 7h). Representative bioluminescence images and X-rays of mice bearing metastases from grafted i.c. PC-3M-luc-C6^*trpv6+/+*^-pTRPV6_wt_ cell clones are shown in Fig. 7i. Notably, the metastasis-free survival of the mice treated with 600 µg/kg mAb82 24 h prior to i.c. grafting was significantly (*p* = 0.0009) greater (Fig. 7j). Most intriguingly, the occurrence of metastasis was decreased by 66.66% (*p* = 0.012) in the group of mice pretreated with 600 µg/kg mAb82 prior to the i.c. injection of PC-3M-luc-C6^*trpv6+/+*^-pTRPV6_wt_ cell clones (Fig. 7k). The presence of bone metastasis was confirmed via H&E and Masson–Goldner’s Trichrome staining of the ribs of mice treated with either mAbAU1- or mAb82-treated mice (Fig. 7l).

These findings demonstrated that the TRPV6/CXCR4 complex is particularly important in vivo; thus, proof-of-concept combined therapy was implemented in vivo. PC-3M-luc-C6^*trpv6+/+*^ cells were grafted subcutaneously in vivo, and as soon as the tumors became palpable, the treatments with either 100 µg/kg mAbAU1/mAb82 or 5 mg/kg AMD3100 were performed twice per week (thrice/week for AMD3100^39,40^ (Fig. 7m and Supplementary Fig. 7m)). Representative images of firefly luciferase staining and the emergence of tumors are shown in Fig. 7n. A significant decrease in tumor growth was observed in the groups treated with 100 µg/kg mAb82 alone or in the group treated with 100 µg/kg mAbAU1 + 5 mg/kg AMD3100 (*p* = 0.014) (Fig. 7o). Intriguingly, the combined treatment of 100 µg/kg mAb82 + 5 mg/kg AMD3100 demonstrated a remarkably efficient (*p* < 0.0001) combination but decreased tumor growth by 80.1% and was 142% more efficient than both the 100 µg/kg mAb82 treatment alone group and the 100 µg/kg mAbAU1 + 5 mg/kg AMD3100 group (*p* = 0.043). A control experiment involving subcutaneous grafting and treatment with PC-3M-luc-C6^*trpv6−/−*^ cell-derived tumors was conducted (Supplementary Fig. 7n–q). Irrespective of small tumor growth, a significant (*p* = 0.022) decrease in tumor growth in the 100 µg/kg mAbAU1 + 5 mg/kg AMD3100 group was observed (Supplementary Fig. 7q).

The efficiency of the combined treatment of mAb82 with AMD3100 was compared with that of the bone metastasis model in which the same PC-3M-luc-C6^*trpv6+/+*^ cells were grafted i.c. (Supplementary Fig. 7r). The overall survival of the mice was significantly greater (*p* = 0.008) in the combined treatment group (100 µg/kg mAb82 + 5 mg/kg AMD3100) than in the mAb82-only group (Supplementary Fig. 7s). The incidence of metastasis was significantly (*p* = 0.04) lower only in the combined group than in the mAb82 group (Supplementary Fig. 7t and u). In addition, the combined treatment group demonstrated both higher metastasis-free survival (*p* = 0.02) and higher survival after metastasis (p = 0.004) rates than did the mAb82 group (Supplementary Fig. 7v–x).

Furthermore, primary tumors from mice bearing subcutaneous xenografts of PC-3M-luc-C6^*trpv6+/+*^ cells treated with either 100 µg/kg mAbAU1/mAb82 or 5 mg/kg AMD3100 were excised at the end of the experiment and subjected to H&E, TRPV6, CXCR4, Ki-67 and TUNEL-fluorescein staining (Fig. 7p). The data revealed an increase in the apoptosis rate and a decrease in Ki-67 staining when both TRPV6 and CXCR4 were blocked with either 100 µg/kg mAb82, 5 mg/kg AMD3100 or a combination of both (Fig. 7p). In addition, a survival assay of PC-3M-luc-C6^*trpv6+/+*^ cells treated with either 6 µg/mL mAbAU1/mAb82 together with 30 mM AMD3100 was performed for 4 days, and the results confirmed the potentiation effect of the mAb82 + AMD3100 combination (Fig. 7q).

These effects were significant when PC-3M-luc-C6^*trpv6−/−*^ cells were treated with 5 mg/kg AMD3100 in combination with the mAbs studied (Supplementary Fig. 7y). Apoptosis induction was analyzed and quantified by flow cytometry via Annexin V/IP staining (Fig. 7r), which revealed that the above treatments were extremely efficient when combined (*p* < 0.0001).

Ulocuplumab (6 µg/ml), a specific anti-CXCR4 receptor antibody,^41,42^ was used to replace AMD3100. Similarly, it was used to selectively inhibit the CXCR4 receptor, which was identical to the effects of AMD3100 on both cell survival (Fig. 7s) and apoptosis induction (Fig. 7t). In the case of PC-3M-luc-C6^*trpv6−/−*^ cells, these effects were observed only when 6 mg/kg ulocuplumab was used in combination with mAbs against AU1 and TRPV6 (Supplementary Fig. 7z).

Finally, the presence of TRPV6/CXCR4 complexes was studied via PLA in PC-3M-luc-C6^*trpv6+/+*^ cells treated with either 6 µg/mL mAbAU1/mAb82 or 30 mM AMD3100 for 36 h, revealing the potentiating effects (*p* < 0.0001) of combined treatment with TRPV6 and CXCR4 (Fig. 7u). Taken together, the present findings clearly demonstrate the strong potential of combined treatment with TRPV6/CXCR4 membrane proteins for the treatment of CRPC and its most aggressive form, bone metastasis.

## Discussion

PCa is a major global health issue, and an in-depth exploration of its molecular complexities is needed to develop effective, predictive and therapeutic strategies.^12^ Our previous studies identified transient receptor potential vanilloid subfamily member 6 (TRPV6) as a key regulator of malignant progression in prostate tumors, influencing both the proliferative capacity and resistance to apoptosis.^24,27,35^ On the basis of these findings, the multifaceted role of TRPV6 in CRPC cell migration, invasion and metastasis was suggested. Unlike other oncochannels, such as TRPM4, TRPM7, and NALCN, which are associated with mesenchymal and invasive markers,^43–45^ TRPV6 is expressed specifically in PCa cells.^46^

The hypothesis that PCa might induce TRPV6 expression de novo to directly benefit from tightly regulated calcium intake via TRPV6 was proposed. This calcium entry was previously reported to be required for the calcium-dependent aggressive phenotype, such as migration and invasion.^47^ Moreover, the expression of the TRPV6 channel provides CRPC cells with a selective advantage for their ability to metastasize to the calcium-abundant niche of bone.

A correlation between TRPV6 expression and the occurrence of metastases in patient samples was established. In addition to its expression in CRPC tumor tissues, TRPV6 becomes de novo expressed in peritumoral regions, which are typically considered nonmalignant. This novel finding suggests that TRPV6 could serve as an early marker of tumor aggressiveness, potentially predicting a risk for malignant transformation. The presence of the TRPV6 protein in peritumoral areas raises important questions as to its role in tumor microenvironment remodeling, facilitating local invasion and promoting early metastatic dissemination. Given its expression in various aggressive cancers,^48,49^ TRPV6-targeting antibodies, as previously published,^28^ are prospective therapeutic strategies.

At the molecular level, the TRPV6 channel was demonstrated to be a crucial regulator of intracellular calcium homeostasis, initiating a signaling cascade involving calcium/calmodulin-dependent protein kinase II (CaMK2) activation and subsequent nuclear translocation of the transcription factor NF-κB. This TRPV6-mediated calcium influx cascade contributes to the acquisition of an aggressive phenotype, triggering a sequence of molecular events involving calmodulin, CaMK2, the NF-κB inhibitor IκB, and NF-κB itself. Notably, the autophosphorylation of CaMK2 at threonine 287 provides mechanistic insight into the increased aggressiveness of prostate cancer cells, underscoring the importance of TRPV6 activity in orchestrating these processes. Although CaMK2 phosphorylation and NF-κB translocation were previously linked to migration and invasion,^50,51^ their direct association with TRPV6-mediated calcium entry has not been demonstrated until now. Additionally, we show that TRPV6 influences the expression of key extracellular matrix (ECM) components, including calpain 2 (Capn2), as well as matrix metalloproteinases, all of which are known to contribute to tumor invasion, epithelial‒mesenchymal transition (EMT), and metastasis.^52,53^ This complex regulation of ECM remodeling via TRPV6 plays an important role in CRPC progression.

Although previous studies have suggested the potential role of TRPV6 silencing on cathepsin B and MMP9 expression in PCa cell lines,^47^ the current research extends this understanding by investigating the role of the TRPV6 channel in three *trpv6*^*−/−*^ models (PC-3M^*trpv6−/−*^, PC-3M-luc-C6^*trpv6−/−*^, and HAP-1^*trpv6−/−*^ cell lines) both in vitro and in vivo. The effects of TRPV6 rescuing were used to decipher the role of TRPV6 in aggressive cell phenotypes, especially castration-resistant phenotypes (CRPC: PC-3M^*trpv6−/−*^ and PC-3M-luc-C6^*trpv6−/−*^-derived cell models), as a result of androgen-deprived therapy (ADT), which represents a majority of aggressive CRPC bone metastasis cases.^54^

The diversity of xenograft models employed in this study reflects different stages of metastasis. The subcutaneous xenografts reflect the initial steps of the metastatic cascade, including blood vessel formation and tumor cell intravasation. Despite the fact that the tumor microenvironment in this model is different at the level of the orthotopic tumor environment, it compensates for the very small size of the mouse prostate (<2 mm) and its actually divergent role in mouse physiology compared with that of humans,^55^ being the best in vivo model for studying all the metastatic steps in the row. The intracardiac injection model effectively mimics the core step of metastatic dissemination and tissue targeting in the blood flow, whereas the intratibial injection model perfectly reproduces the capacity of cancer cells to proliferate within the bone microenvironment. The established CRPC in vivo models, such as PC-3M^*trpv6−/−*^- and particularly PC-3M-luc-C6^*trpv6−/−*^-derived cell models, demonstrate that TRPV6 expression increases bone tropism, a process that appears to be mediated by the upregulation of CXCR4 membrane expression. CXCR4 is a well-known chemokine receptor that has been shown to drive prostate cancer metastasis to bone,^56–58^ whereas our study identified TRPV6 as a key regulator of this pathway. As such, we hypothesized that CXCR4 might be functionally linked to the TRPV6 channel. Indeed, CXCR4 has been reported to be involved in the PIP2/PLC signaling pathway,^36,37^ which modulates endoplasmic reticulum calcium stores. Since TRPV6 was demonstrated to be involved in the response cascade to ER calcium depletion,^35^ this suggests a potential functional interaction between these two elements.^59^ As we showed in the present work, TRPV6 is one of the key players involved in the development of bone metastases. Given the important roles of both CXCR4 and TRPV6, we demonstrated a direct synergistic role between them. Furthermore, recent studies have shown that CXCR4 can also activate signaling pathways involving CAMK2,^60^ and our own phosphoproteomic analyses revealed a significant increase in CAMK2 activity. These findings reinforced our rationale for investigating the interplay between CXCR4 and TRPV6 in this pathological context. Crucially, we revealed a novel and previously unknown molecular interaction of TRPV6 with CXCR4, an association that is strictly dependent on the ion channel activity rather than the expression of TRPV6. This groundbreaking discovery suggests that calcium influx through TRPV6 actively regulates CXCR4 function, further promoting tumor cell migration and bone targeting and colonization. This formation of the TRPV6/CXCR4 complex provides a new mechanistic link between calcium homeostasis and chemokine signaling in metastatic CRPC. As such, the studied mechanisms allow us to comprehensively elucidate the role of TRPV6 in CRPC metastasis initiation and progression, as well as to propose a therapeutic solution *via* the use of a monoclonal antibody that specifically inhibits TRPV6-mediated calcium influx.^28^ TRPV6 was demonstrated to promote key steps in metastasis formation—including intravasation, survival in circulation, extravasation, bone targeting and colonization—thereby facilitating CRPC cell colonization of the bone niche.

The role of other ion channels in bone metastasis has also been studied. Breast cancer-associated TRPM7 and PCa-associated TRPV4 channels have been shown to regulate bone tropism *via* Akt activation,^61,62^ emphasizing the molecular complexity of metastasis. Although the TRPV6 channel has been identified as directly involved in oncogenic processes such as migration and invasion in pancreatic,^63^ colorectal,^64^ and breast cancer,^65^ no data have yet been reported regarding its involvement in the progression of CRPC to a bone metastatic phenotype.

The kinetics of TRPV6-targeting therapy and its effectiveness in blocking metastatic progression were assessed, which revealed a 60% reduction in the metastatic burden at all secondary sites at 100 µg/kg. Notably, as prostate cancer metastasizes to the bone in 91% of cases,^9^ intracardiac injection models demonstrate that administering 600 µg/kg of the antibody 24 h before CRPC cells enter the bloodstream can reduce bone metastasis formation by up to 70%. However, the efficiency of the treatment administered 48 h after intracardiac grafting was lower, i.e., only a 28.6% reduction in the risk of bone metastasis. These findings highlight the importance of optimizing therapeutic efficacy on the basis of treatment strategy. On the other hand, these data suggest that TRPV6 plays a more significant role in the early stages of metastasis initiation and/or anchoring and/or bone tropism than in the later stages of metastatic outgrowth. Notably, unlike human CRPC cases, our in vivo models were not subjected to prior ADT, which is known^66,67^ to condition and prepare the bone matrix for subsequent CRPC cell adhesion in advance. Thus, the effects of TRPV6 channel expression demonstrated are exempt from the preliminary conditioning of the bone niche by ADT and represent the effects mediated by the TRPV6 channel only.

Tumor-induced pressure within the bone microenvironment influences metastasis dynamics.^68^ PCa is distinguished from osteolytic metastases by its ability to stimulate abnormal bone formation, leading to osteoblastic lesions.^3^ Wnt signaling and endothelin-1-mediated fibroblast growth factor receptor activation are key players in this process.^69^ Investigating the involvement of TRPV6, a highly calcium-selective channel, in these calcium-dependent processes could provide further insights into the osteomimetic behavior of prostate cancer cells.^70,71^ Using intratibial graft models, we observed that TRPV6-expressing tumors induce significant bone degradation, in contrast to previous findings suggesting that TRPV6 promotes bone matrix formation.^35^ This discrepancy raises important questions about the context-dependent effects of TRPV6 on bone remodeling. Irrespective of the abovementioned ADT-mediated bone matrix preconditioning, the possibility that TRPV6 dynamically regulates bone metabolism, favoring osteolysis at earlier stages while promoting osteoblastic activity in advanced metastatic lesions, should be considered. Further studies are needed to determine whether TRPV6 influences osteoclast‒osteoblast interactions and calcium homeostasis within the bone microenvironment.

In addition to monotherapy, combined therapy with TRPV6-targeting antibodies together with antiandrogens such as enzalutamide, inhibitors of androgen production such as abiraterone, chemotherapy, or radiotherapy may provide additional benefits for high-risk or metastatic CRPC patients. Calcium homeostasis plays a central role in bone metastasis, with extracellular calcium-sensing receptors influencing tumor cell behavior.^72,73^ High expression of these receptors, such as the aforementioned CXCR4 receptor, is correlated with an increased incidence of bone metastases.^74^

The established proof-of-concept of combination therapy has clearly demonstrated that targeting both TRPV6 with mAb82 and CXCR4, either with the specific inhibitor AMD3100^38^ in vivo or with anti-CXCR4 antibodies such as ulocuplumab^42^ in vitro significantly reduces CRPC cell/tumor growth in a synergistic manner via the induction of apoptosis. Indeed, AMD3100 has already been used in vivo in humans and has the commercial name Plerixafor, which is currently used in several phase I and phase III clinical trials.^75,76^ The use of anti-CXCR4 antibodies such as ulocuplumab has already reached phase I clinical trials.^41^ These findings suggest that targeting both TRPV6 and CXCR4 could represent a powerful therapeutic approach to prevent metastatic progression. Despite recent advances in immunotherapies, treatment options for osteoblastic metastases remain limited. For example, denosumab (XGEVA®), which targets RANKL, is the only FDA-approved immunotherapy for osteolytic bone metastases.^77,78^ Expanding immunotherapeutic strategies, particularly in combination with TRPV6-targeting antibodies, could enhance efficacy in osteoblastic prostate cancer metastases.

In conclusion, the current work provides striking evidence that TRPV6 plays a central role in prostate cancer metastasis, particularly to bone. TRPV6 expression is associated with metastasis risk in patients and promotes an invasive phenotype via the CaMK2/NF-κB pathway. Furthermore, a novel TRPV6-CXCR4 complex was discovered, establishing a direct link between calcium channel activity and chemokine receptor signaling in metastatic progression. These findings also highlight the importance of treatment design and perfect timing, suggesting that early TRPV6 inhibition significantly reduces the risk of bone metastasis emergence and that combining both TRPV6 and CXCR4 inhibitors significantly enhances therapeutic efficacy. In addition, the bone homing model suggested that TRPV6 may have dual roles in bone metastasis, promoting both osteolysis and osteogenesis depending on the metastatic stage. These findings suggest that TRPV6 is both a diagnostic marker and a therapeutic target in CRPC. While TRPV6-targeting strategies, such as mAbs, demonstrate promising preclinical efficacy, further studies are needed to assess their long-term effects, potential combination strategies, and impact on patient outcomes.

## Methods

### In vitro studies

#### Cell lines and reagents

PC-3M (ATCC), PC-3M-luc-C6 epithelial prostate cancer cells (Caliper Life Sciences (Waltham, MA, USA)), LNCaP (ATCC) and LNCaP C4-2B (ATCC) were cultured in Rosewell Park Memorial Institute 1640 medium (RPMI1640); HAP-1 chronic myelogenous leukemia cells (Horizon Discovery) were cultured in Iscove’s modified Dulbecco’s medium (IMDM), and HEK-293 cells (ATCC) and VCaP (ATCC) were cultured in Dulbecco’s modified Eagle’s *medium* (DMEM) and used for electrophysiology experiments. PNT1A (ATCC) cells were cultured in Roswell Park Memorial Institute 1640 (RPMI 1640) medium and used as a normal human prostatic cell line. All basic media were supplemented with 10% fetal bovine serum (FBS), 2 mM L-glutamine, and 100 µg/mL kanamycin in an atmosphere of 95% air and 5% CO_2_ and were maintained at 37 °C. All the media and supplements, if not stated otherwise, were obtained from Gibco (CA, USA). The medium was changed three times a week, and the cultures were split by treating the cells with 0.25% trypsin for 5 min at 37 °C before they reached confluence. The cells were treated every 4 months for potential mycoplasma contamination with MycoZap™ Prophylactic (Lonza, Basel, Switzerland) and 3 weeks before in vivo experiments. KN-93 and AMD3100 were acquired from Millipore Merck (Darmstadt, Germany), human CXCL12 was acquired from Invitrogen (MA, USA), and ulocuplumab was acquired from MedChemExpress (NJ, USA). All the cell models used are listed in Supplementary Table 1.

### Vectors, plasmid construction and siRNA

The whole TRPV6 cDNA containing the 5’-UTR on the pCAGGS vector was provided by Dr. Ulrich Wissenbach from the Universität des Saarlandes, Germany. This sequence was used to obtain a final vEF1ap-5’UTR-TRPV6_CMVp-mCherry vector, vEF1ap-5’UTR-TRPV6-D582A_CMVp-mCherry (E-Zyvec, Loos, France), which was transfected into the cells, and the transfection rate was evaluated via a control vEF1ap-5’UTR_CMVp-mCherry vector.

The cells were transfected with 40 nM small interfering RNA (siRNA) against the mRNA *trpv6* sequence,^79^ with the nonmatching control siRNA used as a negative control (Eurogentec, Seraing, Belgium) or with SMARTpool against the *RelA* sequence (Dharmacon, CO, USA), using the Lipofectamine 3000 transfection reagent according to the manufacturer’s instructions (Thermo Fisher, MA, USA). The efficiency of cell transfection with the siRNAs for each particular target was validated either by real-time quantitative PCR or by immunoblotting.

### Transfection

The cells were transfected via the Lipofectamine 3000 transfection reagent (Thermo Fisher, MA, USA) following the manufacturer’s instructions and were selected with the selective antibiotic G418 at 200 μg/mL and controlled via confocal microscopy to observe the mCherry signal.

### RNA extraction, reverse transcription and real-time PCR

Total RNA was prepared via NucleoSpin RNA Plus (Macherey Nagel, Düren, Germany). Two micrograms of RNA were reverse transcribed via random hexamers, MuLV and dNTPs (Life Technologies), in a final volume of 20 μl according to the manufacturer’s instructions. Quantitative real-time PCRs were performed via a CFX96 real-time PCR system (Bio-Rad, CA, USA). The primers used were designed via qPrimerDepot software (http://primerdepot.nci.nih.gov/). The PCR products were detected and quantified with SsoFastTM EvaGreen® Supermix (Bio-Rad, CA, USA). The experiments were performed in triplicate for each data point. The results were analyzed via CFX Manager version 3.1 software (Bio-Rad, CA, USA). Gene expression was normalized to that of GAPDH, and the fold change in expression relative to that of the control is shown. The primers used are listed in Supplementary Table 2.

### Immunoblotting, biotinylation and antibody arrays

For western blotting, cell lysis was performed via RIPA buffer (20 mM Tris–HCl, 37 mM NaCl, 2 mM EDTA, 1% Triton-X, 10% glycerol, 0.1% SDS, and 0.5% sodium deoxycholate supplemented with phosphatase and protease inhibitors). The total protein content was measured via the BCA assay (Pierce, Boulogne-Billancourt, France). For biotinylation studies, the cell dishes were immediately placed on ice, and the medium was replaced with an ice-cold phosphate-buffered saline (PBSB) solution containing 1 mM MgCl_2_ and 0.5 mM CaCl_2_, pH = 8. Then, the cells were washed once and incubated with PBSB solution containing 2 mM biotin (EZ-Link Sulfo-NHS-LC-LC-Biotin; Pierce Boulogne-Billancourt, France) for 1 h at 4 °C. The cells were then washed once with PBSB solution containing 0.1% BSA and lysed with ice-cold RIPA lysis buffer. Biotinylated proteins were precipitated via neutravidin-agarose beads (Pierce, Boulogne-Billancourt, France), eluted with SDS‒PAGE loading buffer, and subjected to SDS‒PAGE as described below. For immunoprecipitation, protein A/G-Sepharose beads were added to antibodies against the TRPV6 or CXCR4 proteins and incubated for 2 h at 4 °C on a rotating shaker. The cell lysates were subsequently incubated with the above complexes overnight at 4 °C on a rotating shaker. The beads were then centrifuged, washed three times with ice-cold lysis buffer, resuspended in SDS/PAGE loading buffer, and subjected to SDS/PAGE as described below. Proteins were resolved by SDS–PAGE and transferred to PVDF membranes (Hybond-C extra, Healthcare Life Sciences, NJ, USA). Menbranes were saturated with casein during 1 h, washed and incubated with the following primary antibodies: mouse monoclonal anti-TRPV6 (2.4 μg/mL)^28^; mouse monoclonal anti-Orai1 (1/1000) ProSci Cat# PM-5205; rabbit polyclonal anti-TRPC1 (1/1000) Novus Biological Cat# NB100-98844; rabbit polyclonal anti-Stim1 (1/1000) Alomone Labs Cat# ACC-063; rabbit polyclonal anti-N-Cadherin (1/500) Elabscience Cat# E-AB-70061; rabbit polyclonal anti-calpain 2 (1/1000) ABclonal Cat# A1861; rabbit monoclonal anti-MMP2 (1/500) ABclonal Cat# A19080; rabbit polyclonal anti-MMP3 (1/1000) Elabscience Cat# E-AB-60248; mouse monoclonal anti-MMP9 (1/1000) Elabscience Cat# E-AB-60247; mouse monoclonal anti-MT-MMP1 clone C-7 (1/500) SantaCruz Cat# sc-377097; rabbit polyclonal anti-phospho-CaMK2 (Thr287) (1/500) Invitrogen Cat# PA5-37833; rabbit monoclonal anti-CaMK2 clone D11A10 (1/1000) Cell Signaling Cat# 4436; rabbit monoclonal anti-NF-κB/RelA (1/1000) ABclonal Cat# A19653; rabbit polyclonal anti-CXCR4 (1/1000) ABclonal Cat# A13672; mouse monoclonal anti-β-actin (1/1000) ABclonal Cat# A5441. The secondary peroxidase-conjugated antibodies used were from Pierce (Boulogne-Billancourt, France). Peroxidase activity was revealed via enhanced chemiluminescence (ECL) or an enhanced chemiluminescence (ECL) advanced kit (Bio-Rad, CA, United States). Band densities were quantified via ImageJ/Fiji software (version 2.14). The density of each band was divided by the density of the reference protein band or related control band, and the obtained value was normalized with respect to that obtained under control conditions. Fully uncropped and unprocessed blots are available in the Source Data file.

### trpv6^−/−^ cell model generation

The PC-3M^*trpv6−/−*^ cell model was created by Applied Biological Materials Inc. (Marham, Canada) via CRISPR/CAS-9 technology, and gRNA (GAGCTGGTCTTTGAGCCCAT) was transfected with lentivirus, yielding an INDEL. This INDEL resulted in a reading frame shift and a premature stop codon. The knockout was validated via PCR via the sequencing primers 5’-TCCCTTCATAGGATCTGGGAGTC-3’ and 5’-CAACAGATACGGCTGAAGACAG-3’.

The PC-3M-Luc-C6^*trpv6−/−*^ cell model was created by Applied Biological Materials Inc. (Marham, Canada) via CRISPR/CAS-9 technology and gRNA (TCTGCAGATGGTTCCAGAGA) via lentivirus, yielding an INDEL. This INDEL resulted in a reading frame shift and a premature stop codon. The knockout was validated via PCR via the sequence primers 5’-CCTCAACATTGGGAATGGGC-3’ and 5’-CTGTCCAGCCAAAGGTGC-3’.

The HAP-1^*trpv6−/−*^ cell model was created by Horizon Discovery Ltd. (Cambridge, UK) via CRISPR/CAS-9 technology and gRNA (CTCGCACCAGGTTCATGTTC) via lentivirus, which targeted exon 4 and yielded a 1 bp insertion in this exon. This insertion resulted in a reading frame shift and a premature stop codon. The C insertion was validated by sequencing with the sequence primer CTACCCCTGAGGGAAAGAGACTG.

To maintain the *trpv6*^−/−^ status of the cells, the antibiotic selected, puromycin, at 0.1 μg/mL was added to the PC-3M^*trpv6−/−*^ and PC-3M-luc-C6^*trpv6−/−*^ cell lines, and G418 was used at a concentration of 200 μg/mL for maintenance in culture of the HAP-1^*trpv6−/−*^ cells.

### Ca^2+^ imaging

Confocal imaging was performed with an LSM 510 META confocal workstation using a Plan-Neofluar ×40 1.4 numerical aperture objective (Carl Zeiss, Germany). The cells were plated onto glass coverslips and loaded with 4 μM Fura-2 AM at room temperature for 45 min in growth medium. Recordings were performed in HBSS containing the following (in mM): 140 NaCl, 5 KCl, 1 MgCl_2_, 0.3 Na_2_HPO_3_, 0.4 KH_2_PO_4_, 4 NaHCO_3_, 5 glucose, and 10 HEPES adjusted to pH 7.4 with NaOH. CaCl_2_ was adjusted to 0 mM or 2 mM, depending on the experiment. The coverslips were then placed in a perfusion chamber on the stage of the microscope. Fura-2 fluorescence at an emission wavelength of 510 nm was recorded by exciting the probe alternatively at 340 and 380 nm. Acquisition and analysis were performed via Metafluor 4.5 software (Universal Imaging Corp.).

### Electrophysiology and solutions

Macroscopic currents were recorded from HEK-293 cells transfected with either the vEF1ap-5’UTR-TRPV6_CMVp-mGFP or vEF1ap-5′UTR-TRPV6-D582A_CMVp-mGFP or vEF1ap-5′UTR_CMVp-mGFP vector in the whole-cell configuration of the patch-clamp technique at room temperature via an Axopatch 200B amplifier and a Digi-data 1322 A digitizer (Molecular Devices, USA). The composition of the extracellular solution for patch-clamp recording was as follows (in mM): 140 NaCl, 5 KCl, 2 CaCl_2_, 1 MgCl_2_, 10 glucose, and 10 HEPES, pH 7.4, adjusted with NaOH. The patch pipettes were filled with the following solutions (in mM): 145 CsCl, 10 HEPES, 5 EGTA (1.2-bis(2-amonophenoxy)ethane N,N,N’,N’tetraacetic acid), and 2 MgCl_2_ (pH adjusted to 7.2 with CsOH and an osmolarity of 295 mOsm/l adjusted with D-mannitol). To reveal TRPV6 activity and increase Ca^2+^-dependent TRPV6 inactivation, the initial extracellular solution was replaced with a divalent-free solution (DVF) with the same ionic composition as above, where both CaCl_2_ and MgCl_2_ were replaced with an additional 5 mM KCl, yielding the following concentrations in mM: 140 NaCl, 10 KCl, 10 glucose, and 10 HEPES, pH 7.4 adjusted with NaOH, as described previously.^28^

### Confocal imaging

The cells grown on glass coverslips were washed with PBS and immediately fixed in 4% paraformaldehyde in PBS + 0.05% glutaraldehyde. PBS-glycine (30 mM) was used to quench the reaction, which was subsequently permeabilized with 0.2% Triton X-100 where necessary. The cells were then washed again in PBS, and nonspecific sites were blocked with 5% donkey serum solution, followed by a conventional immunostaining procedure. The cells were incubated with specific antibodies, such as mouse monoclonal anti-TRPV6 (6 μg/μL)^28^; mouse monoclonal anti-vimentin clone D9 (1/800) Dako Cat# M0725; rabbit polyclonal anti-N-cadherin (1/500) Elabscience Cat# E-AB-70061; rabbit polyclonal anti-E-cadherin (1/500) Elabscience Cat# E-AB-31261; rabbit polyclonal anti-phospho-CaMK2 (Thr287) (1/500) Invitrogen Cat# PA5-37833; and rabbit monoclonal anti-NF-κB p65/RelA (1/250) ABclonal Cat# A19653, overnight at 4 °C. Alexa Fluor® 488 donkey anti-mouse IgG (Sigma, Saint-Quentin-Fallavier, France, 1/4000) was used as a secondary antibody for TRPV6 and vimentin staining, and Alexa Fluor® 488 donkey anti-rabbit IgG (Sigma, Saint-Quentin-Fallavier, France, 1/4000) was used as a secondary antibody for N-cadherin, E-cadherin, pCaMK2 and NF-κB staining. Fluorescence analysis was carried out via a Carl Zeiss Laser Scanning Systems LSM 700 connected to a Zeiss Axiovert 200M with a ×40/×63 1.4 numerical aperture oil immersion lens at room temperature. Both channels were excited, collected separately, and then merged via Carl Zeiss LSM Image Examiner software (3.1.0.99).

### FACS analysis

Flow cytometry assays were performed on cell populations cultured in 75 cm^2^ flasks. Approximately 4 × 10^6^ cells were fixed with 1% paraformaldehyde in PBS on ice for 15 min. After fixation, the cells were washed with 3% PBS-BSA and pelleted with 0.25% trypsin for 5 min at 37 °C. Then, the cells were resuspended in 3% PBS-BSA and stained with 15 µg/ml mouse monoclonal anti-TRPV6 antibody^28^ or 15 µg/ml rabbit polyclonal anti-CXCR4 antibody (clone D4Z7W, Cell Signaling Cat# 97680) for 2 h, followed by 3 washes. The cells were then stained with an anti-mouse FITC-conjugated IgG antibody at 1/100 for 1.5 h. The stained cells were stored at 4 °C in the dark and analyzed within 2 h. To analyze only membrane staining, a permeabilization control with the addition of DRAQ7™ (Invitrogen, MA, USA) at a final concentration of 3 μM was performed just before analysis. The stained samples were measured on a BD LSR Fortessa flow cytometer (Becton–Dickinson, CA, USA). Data were acquired for at least 10000 events with a variation coefficient of less than 5%, and green fluorescence was measured via a fluorescence detector 3 (FL3) on the *X*-axis. The data were stored and analyzed via Kaluza software.

### Annexin V/propidium iodide analysis

Flow cytometry was used to detect the number of apoptotic cells and the cell cycle distribution. The cells were harvested following incubation with different treatments for 72 h. To analyze apoptosis via Annexin V/PI (propidium iodide) staining, the cells were stained with the FITC Annexin V Apoptosis Detection Kit I (BD Pharmingen Inc., CA, USA) according to the manufacturer’s instructions. The stained samples were measured on a BD LSR Fortessa flow cytometer (Becton–Dickinson, CA, USA). Data were acquired for at least 250,000 events with a variation coefficient of <5%. The data were stored and analyzed via Kaluza software.

### Luciferase activity assay

PC-3M-luc-C6 cells (5.0 × 10^3^; 10.0 × 10^3^; 20.0 × 10^3^) were seeded into a 96-well plate with a black, clear bottom. Twenty-four hours later, the luminescent activity was assessed using 150 μg of D-luciferin (Promega, WI, USA). The data are presented as the relative luminescence unit (RLU) with the firefly luciferase signal.

### NF-κB Luciferase activity assay

PC-3M cells were seeded into a black clear-bottom of a 96-well plate 24 h before transfection. The cells were transfected with the reporter plasmid pCDH_NF-κB_Dual_Luc via Lipofectamine 3000 following the manufacturer’s instructions. Twelve hours posttransfection, the cells were stimulated with either 20 ng/mL TNF-α or SMARTpool against *RelA* mRNA. Twenty-four hours posttransfection, luminescence activity was assessed with the Nano-Glo® Dual-Luciferase® Reporter (NanoDLR™) Assay System (Promega, WI, USA). The data are presented as relative luminescence units (RLUs) normalized to the firefly luciferase signal by NanoLuc® luciferase activity.

### Phospho-IκB alpha (S32) ELISA

The cells were cultured in complete medium and incubated in 5% CO_2_ in a humidified atmosphere at 37 °C for 1–2 days. After this period, 4 × 10^6^ cells/ml were scraped into Cell Lysate Buffer. Phosphorylation of IκB alpha (S32) was measured via both phospho-IκB alpha (S32) and total IκB alpha ELISA kits (Abcam, Cambridge, UK) according to the manufacturer’s instructions, with the plates assayed on a 96-well plate reader at 450 nm.

### Migration and invasion assays

*Wound healing assay*: Cells were seeded and incubated overnight at 37 °C until they reached the subconfluent stage. A pipette tip was used to uniformly scratch the subconfluent cell layers, followed by washing and starvation to prevent further proliferation. Images of the initial wound were obtained via an inverted microscope (Nikon Eclipse TS100). To inhibit CaMK2, KN-93 was applied at 1 or 20 μM. Images were obtained at 8 and 18 h time points at the same initial sites. The distance between the 2 migration fronts was measured via ImageJ/Fiji version 2.14 software.

*Transwell® assay*: Cells (2.5 × 10^4^) were plated into the upper chamber of 8 μm pore Transwell® inserts (Falcon™) in serum-free media, whereas the lower compartment was filled with standard growth media as a chemoattractant. For the invasion assay, inserts were precoated with 1.76 mg/mL Matrigel™ Basement Membrane Matrix (Becton Dickinson), and 4 × 10^4^ cells were plated. To inhibit CaMK2, KN-93 was applied to the upper chamber at 10 or 20 μM, and to inhibit CXCR4, AMD3100 was applied to the upper chamber at 30 μM during the assay. To study the effects of CXCL12 as a chemoattractant, human CXCL12 was added to the lower chamber at 100 ng/mL. After 48 h at 37 °C and 5% CO_2_, the cells were fixed in ice-cold 100% methanol and stained with 1% crystal violet in 25% methanol. The inserts were then washed, and the upper area of the filter was rubbed dry to eliminate nonmigrated cells. The data were acquired via an inverted Nikon Eclipse TS100 microscope. The results of the cell counts from five randomly selected fields were averaged. At least two inserts for each condition were analyzed.

### Time-lapse microscopy

The cells were seeded at low density, stained with Hoechst at 2 µg/ml (for 30 min prior to recording) and incubated at 37 °C under 5% CO_2_ in an incubator for time-lapse video recording. The acquisition was conducted over a 48-h period and 20-h period for the PC-3M and HAP1 cell lines, respectively, with the images captured at 30-min intervals via a Nikon Biostation IM incubated with a video microscope (Nikon Europe B.V.) equipped with a ×20 objective lens. The imaging setup included both phase contrast and fluorescence (Hoechst vital dye, Invitrogen, #33342). To optimize image quality while minimizing photoinduced stress, a binning factor of 2 × 2 and a gain setting of 2 were consistently applied across all the imaging channels. Tracking was performed via ImageJ/Fiji version 2.14 software to calculate both distance and speed.

### Proliferation Assay

Cell proliferation was measured via the CellTiter 96® Aqueous One Solution (Promega, Madison, WI), which is based on the conversion of the colorimetric reagent MTS [3,4-(5-dimethylthiazol-2-yl)-5-(3-carboxymethoxyphenyl)-2-(4-sulfophenyl)-2H-tetrazolium salt] into soluble formazan by dehydrogenase enzymes found only in metabolically active and proliferating cells. Following each treatment, 20 μL of dye solution was added to each well of a 96-well plate and incubated for 45 min at 37 °C. The absorbance was subsequently recorded at a wavelength of 490 nm via a plate reader (Molecular Devices, Sunnyvale, CA). The cellular proliferation inhibition rate was calculated as (*A*_control_−*A*_sample_)/(*A*_control_−*A*_blank_)×100%.

### Zymography

Zymography was performed via 10% SDS–PAGE with 1% gelatin. The cells were grown in FBS-free media for 24 h, after which the media were collected. The media was centrifuged and loaded without the addition of reducing agents, after which the mixture was boiled. The zymograms were developed with 0.5% Coomassie G-250 in 30% ethanol and 10% acetic acid.

### RNA sequencing and bioinformatics analysis

For RNA sequencing, the cells were plated in 25-cm^2^ dishes at 75% confluence, and total RNA was extracted and purified from the cells via the NucleoSpin® RNA Plus Kit (Macherey-Nagel, Strasbourg, France) according to the manufacturer’s recommendations. Each RNA sample (three replicates per group) was validated for RNA integrity via an 18S/28S ratio. Then, 1 μg of total RNA from each sample (*n* = 10) was used for library preparation. Library preparation was performed following the manufacturer’s recommendations (Illumina Stranded mRNA Prep). Finally, pooled library samples were sequenced on an ILLUMINA NovaSeq 6000 with an SP-200 cartridge (2 × 800 million 100-base reads), corresponding to 2 × 26 million reads per sample after demultiplexing. This work benefited from equipment and services from the iGenSeq core facility at ICM (Paris, France).

*Analysis*: This work benefited from equipment and services from the iGenSeq core facility at ICM (Paris, France). Bioinformatics analysis of the RNA-seq read data was performed at the IGenSeq Platform Brain Institute. The quality of the raw data was evaluated with FastQC. Poor-quality sequences and adapters were trimmed or removed with DRAGEN, with default parameters, to retain only good-quality paired reads. The Illumina DRAGEN bio-IT platform (v3.10.4) was used for mapping the hg38 reference genome and for quantification with the gencode v37 annotation gtf file. Library orientation, library composition, and coverage along transcripts were checked with Picard tools. The following analyses were conducted with R software. The data were normalized with the DESeq2 bioconductor package and DESeq2 workflow. Multiple hypothesis-adjusted *p* values were calculated via the Benjamini–Hochberg procedure to control the FDR. Finally, enrichment analysis was conducted via the clusterProfiler R package with gene set enrichment analysis via the KEGG pathway database.

### Human cytoskeleton phospho-array

A phosphorylation-specific antibody microarray (Fullmoon Biosystems, Inc.) was used to determine the up- and downregulated proteins in PC-3M^*trpv6−/−*^-mCherry and PC-3M^*trpv6−/−*^-pTRPV6_wt_ cells. The array contained 141 site-specific antibodies against phosphorylated and unphosphorylated proteins involved in cytoskeletal pathways, each of which was replicated six times. Actin and GAPDH were used as controls. The assay was performed following the manufacturer’s protocol.

### Proximity ligation assay (PLA)

The cells were seeded on slides and fixed with 4% paraformaldehyde for 10 min. The cells were treated with saturation buffer (NDS 5% (v/v), BSA 1% (v/v), 1 h, 20 °C) before primary antibody incubation (4 °C, overnight). After the washing step (PBS, 4 × 5 min, 20 °C), PLA was performed via a Duolink in Situ-Green Mouse/Rabbit Kit (Sigma‒Aldrich) according to the manufacturer’s protocol. Labeling was visualized via Plan Fluo ×63 1.4 numerical aperture oil immersion lenses via a laser scanning confocal microscope (LSM 700, Zeiss). An in-house automatic script on ImageJ was used to estimate the total number of TRPV6/CXCR4 green dots per cell. First, the channels were split, and an appropriate background subtraction was found for each channel to enable accurate quantification. To do so, the Gaussian blur, the sharpening function and the threshold were optimized together to enhance the noise/signal ratio. The PLA signals and the nuclei were quantified on the basis of the fluorescence intensity, size and circularity of the particles.

### Immunohistochemistry and immunohistofluorescence

#### Human prostate tissue and bone metastases

Paraffinized anonymous human prostate tissue sections from 37 prostatectomies and paraffinized decalcified human bone metastasis tissue sections from 7 biopsies were obtained from the Department of Cell Pathology, Hôpital St. Vincent de Lille (ethical protocol RNIPH-2022-26). Paraffin-embedded prostate tissue sections were subjected to conventional deparaffinization followed by antigen retrieval via citrate buffer at 95 °C in a water bath. After saturation in a solution containing 1% BSA and 0.05% Triton X-100 in PBS, the prostate sections were incubated with specific antibodies, such as mouse monoclonal anti-TRPV6 antibody (6 μg/μL), overnight at 4 °C. Donkey polyclonal anti-mouse peroxidase-conjugated secondary antibodies (Chemicon International, CA, USA; 1/200) were used. After visualization with diaminobenzidine (Sigma‒Aldrich, Saint-Quentin-Fallavier, France), the images were analyzed via a Zeiss Axioscope microscope (Carl Zeiss, Zaventem, Belgium) and Leica Image Manager software (Leica Geosystems AG Heinrich, Heerbrugg, Switzerland). Immunohistochemistry was performed automatically via a Benchmark XT automated slide stainer (Ventana Medical Systems, Inc., Tucson, AZ, USA) following established protocols, and detection was performed via an IVIEW-DAB detection system (N760-500, Ventana Medical Systems, Inc., Oro Valley, AZ, USA). All prostate tissues were subjected to H&E staining for Gleason score characterization, and all bone metastases were subjected to various types of staining to confirm the presence of metastases (cytokeratin AE1/AE3, NKX3.1, PSA, and pankeratin). Pathological anatomy evaluation was carried out by Dr. Mihalache and Prof. Gosset from Hôpital St. Vincent de Lille.

#### Mouse primary tumors and metastases

Tumors were harvested from the mice, fixed in 4% paraformaldehyde for 48 h and embedded in paraffin. Paraffin-embedded tissue sections were subjected to conventional deparaffinization followed by antigen retrieval via citrate buffer at 95 °C in a water bath, if necessary. After saturation in a solution containing 1% BSA and 0.05% Triton X-100 in PBS, the sections were incubated with specific antibodies, such as mouse monoclonal anti-TRPV6 antibody (6 μg/μL)^28^; goat polyclonal anti-Cathepsin B clone S-12 (1/600) Santa Cruz Cat# sc-6493; rat monoclonal anti-CD29 clone 9EG7 (1/800) BD Bioscience Cat# 553715; mouse monoclonal anti-FAK clone H-1 (1/600) Santa Cruz Cat# 1688; rabbit monoclonal anti-VEGF clone EP1176Y (1/800) Abcam Cat# AB52917; and rabbit polyclonal anti-phospho-CaMK2 (Thr287) (1/500) Invitrogen Cat# PA5-37833, overnight at 4 °C. Donkey polyclonal anti-mouse, anti-rabbit or anti-rat peroxidase-conjugated secondary antibodies (Chemicon International, CA, USA; 1/200) were used. After visualization with diaminobenzidine (Sigma‒Aldrich, Saint-Quentin-Fallavier, France), the images were analyzed via an inverted microscope (Carl Zeiss, Zaventem, Belgium) and Leica Image Manager software (Leica Geosystems AG Heinrich, Heerbrugg, Switzerland). For the immunohistofluorescence procedure, after deparaffinization and saturation, the sections were incubated with specific antibodies, such as mouse monoclonal anti-TRPV6 antibody (6 μg/μL)^28^ and rabbit polyclonal anti-CaMK2 (Thr287) (1/500) Invitrogen Cat# PA5‒37833, rabbit monoclonal anti-NF-κB p65/RelA (1/250) ABclonal Cat# A19653, rat monoclonal anti-Ki67 clone SolA15 (1/100) Invitrogen Cat# 14‒5698‒82 or rabbit polyclonal anti-CXCR4 clone D4Z7W (1/200) Cell Signaling Cat# 97680, overnight at 4 °C. Alexa Fluor® 488 donkey anti-mouse IgG (Sigma, Saint-Quentin-Fallavier, France, 1/4000) was used as a secondary antibody for TRPV6 staining, and Alexa Fluor® 488 donkey anti-rabbit IgG (Sigma, Saint-Quentin-Fallavier, France, 1/4000) was used as a secondary antibody for pCaMK2 staining. Fluorescence analysis was carried out via a Carl Zeiss Laser Scanning Systems LSM 700 connected to a Zeiss Axiovert 200M with ×40/×63 1.4 numerical aperture oil immersion lenses at room temperature. Both channels were excited, collected separately, and then merged via Carl Zeiss LSM Image Examiner software (3.1.0.99).

#### Mouse bone organs

Bones were harvested from the mice and fixed in 4% paraformaldehyde for 48 h. Fixed bones were then decalcified with 14% EDTA solution at 4 °C for an additional 10 days. Decalcified bones were embedded in paraffin. Paraffin-embedded tissue sections were subjected to conventional deparaffinization followed by antigen retrieval via citrate buffer at 95 °C in a water bath. After saturation in a solution containing 1% BSA and 0.05% Triton X-100 in PBS, the bone sections were incubated with specific antibodies, such as mouse monoclonal anti-TRPV6 (6 μg/μL),^28^ rabbit polyclonal anti-CXCR4 clone D4Z7W (1/200) Cell Signaling Cat# 97680), and rat monoclonal anti-Ki67 clone SolA15 (1/100) Invitrogen Cat# 14-5698-82, overnight at 4 °C. Donkey polyclonal anti-mouse or anti-rabbit peroxidase-conjugated secondary antibodies (Chemicon International, CA, USA; 1/200) were used. After visualization with diaminobenzidine (Sigma‒Aldrich, Saint-Quentin-Fallavier, France), the images were analyzed via an inverted microscope (Carl Zeiss, Zaventem, Belgium) and Leica Image Manager software (Leica Geosystems AG Heinrich, Heerbrugg, Switzerland). For the immunohistofluorescence procedure, following deparaffinization and saturation, the sections were incubated with specific antibodies, such as mouse monoclonal anti-TRPV6 (6 μg/μL)^28^ and rabbit polyclonal anti-CXCR4 clone D4Z7W (1/800), Cell Signaling Cat# 97680), overnight at 4 °C. Alexa Fluor® 488 donkey anti-mouse IgG (Sigma, Saint-Quentin-Fallavier, France, 1/4000) was used as a secondary antibody for TRPV6 staining, and Alexa Fluor® 546 donkey anti-rabbit IgG (Sigma, Saint-Quentin-Fallavier, France, 1/4000) was used as a secondary antibody for TRPV6 staining. Fluorescence analysis was carried out via a Carl Zeiss Laser Scanning Systems LSM 700 connected to a Zeiss Axiovert 200M with ×40/×63 1.4 numerical aperture oil immersion lenses at room temperature. Both channels were excited, collected separately, and then merged via Carl Zeiss LSM Image Examiner software (3.1.0.99).

Masson-Trichrome Goldner staining was carried out according to the manufacturer’s recommendations (Sigma‒Aldrich, MA, USA) to confirm the localization of the metastases in the bone region. Pathological anatomy evaluation was carried out by Dr. Mihalache from Hôpital St. Vincent de Lille via H&E and pankeratin staining to confirm the presence/absence of metastases.

### TUNEL assay

The level of apoptosis was estimated from the number of apoptotic nuclei revealed by a TUNEL-fluorescein assay (Roche Biochemicals, as described by the manufacturer). The percentage of apoptotic cells was determined by counting at least three random fields for each condition in triplicate.

### In vivo studies

#### Study approval

Studies involving animals (Ethical Committee Protocol 201703021400830 and Ethical Committee Protocol 2022112411295466), including housing and care, methods of euthanasia and experimental protocols, were conducted in accordance with the animal ethical committee CEEA75 in the animal house PHExMAR (H59-00913) of the University of Lille under the supervision of Dr. Lehen’kyi, Dr. Haustrate and Mr. Cordier. Mice for subcutaneous, intracardiac and intraosseous injections were purchased from Charles River and handled according to the French Ministerial Decree No. 87-848 of 19 October 1987.

### In vivo fluorescence and X-ray imaging

#### In vivo fluorescence

Mice were injected intraperitoneally with 150 mg/kg D-luciferin (Promega, WI, United States) and imaged in vivo 10 min after injection with an In Vivo Xtreme Optimal Imaging System (Brüker, USA). Luminescence was quantified as flux (P/s/mm^2^), for which the imaging settings and time were held constant. Analysis was performed (Living Image Software, PerkinElmer, 4.7.3), maintaining the region of interest over the tissues at a constant and identical size.

#### X-ray

Radiography of the animals was performed weekly starting 7 days after grafting via an X-ray small animal system (45 kVp) with a 0.5 mm aluminum filter from the In Vivo Xtreme Optimal Imaging System (Brüker, USA). Analysis was performed (Living Image Software, PerkinElmer, 4.7.3), maintaining the region of interest over the tissues at a constant and identical size. The dissected bones were then processed for histologic analyses and confirmation.

### Subcutaneous grafting

Five- to six-week-old male Swiss nude mice (Charles River Laboratories) were injected subcutaneously with 2 × 10^6^ PC-3M^*trpv6−/−*^-mCherry and PC-3M^*trpv6−/−*^-pTRPV6_wt_ cells or PC-3M-luc-C6^*trpv6−/−*^ and PC-3M-luc-C6^*trpv6+/+*^ cells suspended in 50% (v:v) Matrigel® (Corning). Tumor growth was recorded once per week via a sliding caliper. Primary tumors were excised once the size of the tumors reached 2500 or 2000 mm^3^ or on Day 36 according to the protocol. Both mCherry fluorescence and the firefly luciferase signal (after injection of 150 mg/kg D-luciferin) emitted by tumor cells were recorded via an in vivo Xtreme Optimal Imaging System (Brüker, USA) and used to follow the development of primary tumor growth or metastases, respectively. The mice were sacrificed as soon as either a critical size of 2500 or 2000 mm^3^ was reached (depending on the ethical protocol), or a deviation of more than 10% of body weight was recorded.

### Intracardiac injections

Five- to six-week-old male Swiss Nude Mice (Charles River Laboratories) were injected into the left ventricle via a 29-G needle with 2 × 10^6^ different stable clones of PC-3M-luc-C6^*trpv6+/+*^ and 4 × 10^6^ clones of PC-3M-luc-C6^*trpv6+/+*^ or PC-3M-luc-C6^*trpv6−/−*^ cells suspended in 100 μl of PBS. The mice were maintained under anesthesia with isoflurane (Virbac) during the injection procedure. Bioluminescence imaging was performed 15 min after intracardiac injection to monitor cell dissemination. Only animals with no (diffused) signal throughout the body were considered for further analysis. Animals were monitored weekly after injection via an In Vivo Xtreme Optimal Imaging System (Brüker, USA).

### Intraosseous injections

Five- to six-week-old male Swiss nude mice (Charles River Laboratories) were injected into the bone marrow of the left tibia via a 26-G needle to prepare the area, and a Hamilton syringe was used to inject 2.5 × 10^5^ stable cell clones of PC-3M-luc-C6^*trpv6+/+*^ or 5 × 10^5^ cell clones of either PC-3M-luc-C6^*trpv6+/+*^ or PC-3M-luc-C6^*trpv6−/−*^ cells resuspended in 20 μl of PBS. The right tibia was used for the control injection of 20 μl of PBS. The mice were maintained under isoflurane anesthesia during the injection procedure. Fifteen minutes before the injection, the mice received 2 mg/kg lidocaine at the site of injection, followed by 0.05 mg/kg buprenorphine i.p. every 8 h for 24 h to limit pain according to the ethical committee. Bioluminescence imaging was performed after intraosseous injection to monitor the injection quality. The animals were monitored weekly after injection.

### In vivo treatments

For subcutaneous PC-3M cell grafting, once the tumors reached 100 mm^3^ in size, the mice were randomized for treatment (at least 7 animals/group) and received an intraperitoneal injection of either mAbAU1 or mAb82 at 100 µg/kg of body weight diluted in PBS twice per week. For subcutaneous PC-3M-luc-C6 cell grafting, once the tumors reached 100 mm^3^ in size, the mice were randomized for treatment (at least 7 animals/group) and received an intraperitoneal injection of either mAbAU1 or mAb82 at 100 µg/kg body weight and/or AMD3100 at 5 mg/kg body weight or PBS twice per week. For intracardiac injections, two protocols were used: one with an intraperitoneal bolus injection of mAbAU1 or mAb82 at 100 or 600 µg/kg, 24 h before cell grafting, and the other protocol (at least 10 animals/group), 100 µg/kg, 48 h after grafting. Mice were randomized to treatment groups (at least 10 animals/group) and received intraperitoneal injections of mAbAU1 or mAb82 at 100 µg/kg body weight diluted in PBS twice per week.

### X-ray micro-CT analysis

Following sacrifice, the collected tibia samples were scanned via an in vivo high-resolution X-ray micro-CT instrument (Quantum GX2, Rigaku, Life Sciences, Revvity) hosted by the Life Imaging Facility (Université Paris Cité and Sorbonne Paris Nord, Inserm UMR1333, Santé Orale, Plateforme Imageries du Vivant, F-92120 Montrouge, France). Standard acquisition settings were applied (voltage 90 kV, intensity 88 µA and aluminum 0.5 mm filter), and 3D scans were performed with a field of view of 5 mm, focused on the tibial proximal metaphysis, with a scan time of 240 s and a voxel size of 10 μm^3^ on the region of interest (ROI). Image acquisition and microarchitectural analysis were performed according to published guidelines.^80^

The trabecular bone was analyzed on the tibial proximal metaphysis for each collected sample. For the analysis, image stacks were first oriented in the same direction by using DataViewer (SkyScan, release 1.6.0.0; Kontich, Belgium) to determine the region of interest. The ROIs were adjusted according to the tibia size, and two hundred slices in the ROI. Quantification of the proximal metaphysis bone microarchitecture (BMA) below the growth plate was performed via CT-Analyzer software (SkyScan, release 1.20.8.0).

After image segmentation, the software was used to quantify the three-dimensional BMA parameters of the image stacks for trabecular and cortical bone: the ratio of bone volume/total volume (BV/TV; %), trabecular number (Tb.N; mm^−1^), trabecular separation (Tb.Sp; mm), trabecular thickness (Tb.Th; mm), ratio of cortical/total cross-sectional area (Ct.Ar/Tt.Ar; %), cortical thickness (Ct.Th; mm) %), total volume of pores (Po,V(tot); mm^3^) and total porosity (Po(tot); %). Three-dimensional volume rendering was performed for the trabecular and cortical bone with CTvol software (SkyScan, release 2.2.3.0) and Dragonfly (Comet, release 2022.2 build 1399).

### Human dataset analysis

For analysis of the GSE dataset, human prostate tumors for which TRPV6 status was known were used for the assessment of TRPV6 expression compared with aggressivity. Expression analysis was performed via published datasets downloaded from the PCaDB (http://bioinfo.jialab-ucr.org/PCaDB/), including peritumoral tissue, primary tumors, primary tumors with metastases, only bone metastases and metastases without bone (GSE32571; GSE32269; GSE77930). Z scores were calculated via normalized data from each dataset by subtracting the population mean from individual expression values for each gene and then dividing the difference by the population standard deviation (SD).

### Ethics statement

Studies involving human samples were performed in accordance with the Declaration of Helsinki. All patient samples studied were approved by the Ethics Committee for Research of Groupement des Hôpitaux de l’Institut Catholique de Lille Hôpital Saint Philibert (GHICL-IRB 00013355/RNIPH-2022-26). Sample recruitment was not performed specifically for our study.

### Statistics and reproducibility

For in vitro studies, no randomization was performed, and experiments were repeated at least three times and validated with independent cell lines. Representative experiments were repeated as indicated, with similar results. Age-matched mice were randomized into control and experimental groups at the start of the experiment on the basis of their body weight. No data were excluded from the analyses. Statistical analysis was performed via GraphPad Prism v10.3.1. All comparisons between two groups were made via a two-tailed, unpaired *t*-test. When more than two groups were compared, one-way ANOVA was performed with Holm–Sidak’s multiple-comparison test. All the data are presented as the means ± SEMs with similar variances between groups. For the survival analysis, the log-rank test was performed, and the *p*-value is indicated. All the statistical analyses were performed via GraphPad Prism software. *p*-values < 0.05 were considered statistically significant.

## Supplementary information

Supplementary Materials

All videos tracks and RNAseq data

## Data Availability

The underlying data for the manuscript can be accessed via the corresponding author upon request. The original RNA-sequencing data presented in the study are publicly available. This data can be found here: https://www.ncbi.nlm.nih.gov with the accession number: GSE231352.
