# Supplementary material for: Targeting TRPV6/CXCR4 complexes prevents castration-resistant prostate cancer metastasis to the bone
Source: Signal Transduct Target Ther. 2025 Sep 5;10:287. doi: 10.1038/s41392-025-02376-8 (PMC12411641; doi:10.1038/s41392-025-02376-8)
Supplement: Supplementary file 1 — Supplementary Materials [file 41392_2025_2376_MOESM1_ESM.docx]

Supplementary Materials for

**Targeting TRPV6/CXCR4 complexes prevents castration-resistant prostate cancer metastasis to the bone**

Clément Cordier, Aurélien Haustrate, Adriana Mihalache, Erika Duval, Emilie Desruelles, Corentin Spriet, Baptiste Casel, Lotfi Slimani, Benjamin Soret, Laurent Allart, George Shapovalov, Pierre Gosset, Natalia Prevarskaya, V’yacheslav Lehen’kyi.

Correspondence to: [vyacheslav.lehenkyi@univ-lille.fr](mailto:vyacheslav.lehenkyi@univ-lille.fr)

**This PDF file includes:**

Supplementary Figures 1 to 7

Supplementary Tables 1 to 2

**Other Supplementary Materials for this manuscript include the following:**

Data S1 to S3

Supplementary Figure 1.


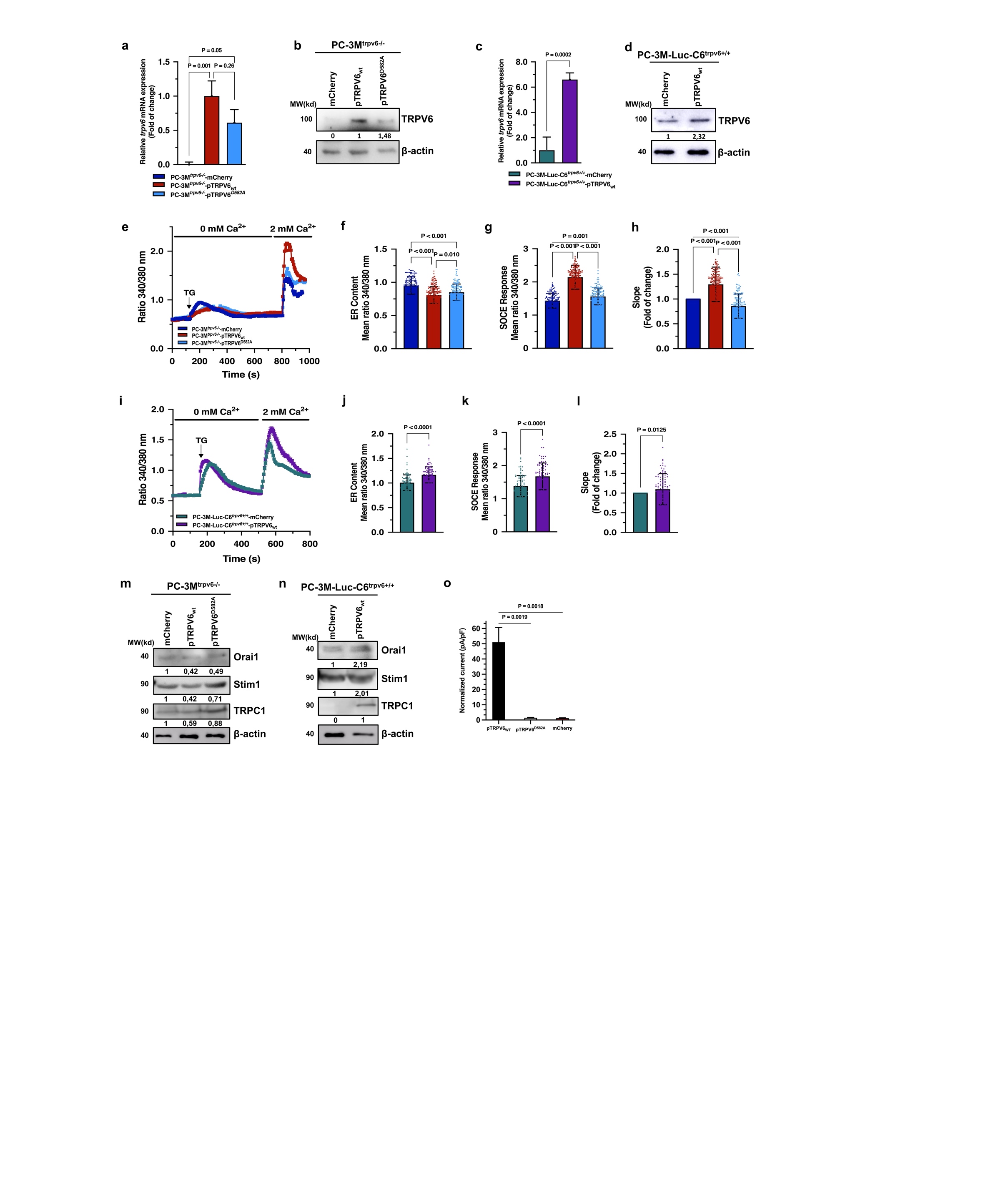
 **Supplementary Figure 1. Validation of prostate cancer cell lines used in the study. (a)** Relative mRNA expression of TRPV6 in PC-3M*^trpv6−/−^*-mCherry, PC-3M*^trpv6−/−^*-pTRPV6_wt_, and PC-3M*^trpv6−/−^*-pTRPV6^D582A^ stable cell clones. **(b)** TRPV6 protein expression in stable clones from **a**. **(c)** Relative mRNA expression of TRPV6 in PC-3M-luc-C6*^trpv6+/+^*-mCherry and PC-3M-luc-C6*^trpv6+/+^*-pTRPV6_wt_ stable cell clones. **(d)** TRPV6 protein expression in stable clones from **c**. **(e)** Cytosolic Ca^2+^ imaging (using Fura-2) performed using SOCE protocol in PC-3M*^trpv6−/−^*-mCherry, PC-3M*^trpv6−/−^*-pTRPV6_wt_, and PC-3M*^trpv6−/−^*-pTRPV6^D582A^ stable cell clones. **(f)** ER relative calcium content (shown as a fluorescence ratio) following thapsigargin-induced Ca^2+^ release from ER demonstrated in **e**. **(g)** SOCE (shown as a fluorescence ratio) following thapsigargin-induced Ca^2+^ release demonstrated in **e**. **(h)** Slope of SOCE following thapsigargin-induced Ca^2+^ release from ER demonstrated in **e**. **(i)** Cytosolic Ca^2+^ imaging (using Fura-2) performed using SOCE protocol in PC-3M-luc-C6*^trpv6+/+^*-mCherry and PC-3M-luc-C6*^trpv6+/+^*-pTRPV6_wt_ stable cell clones. **(j)** ER relative calcium content (shown as a fluorescence ratio) following thapsigargin-induced Ca^2+^ release from ER demonstrated in **i**. **(k)** SOCE (shown as a fluorescence ratio) following thapsigargin-induced Ca^2+^ release demonstrated in **i**. **(l)** Slope of SOCE following thapsigargin-induced Ca^2+^ release from ER demonstrated in **i**. **(m)** Protein expression of Orai1, Stim1 and TRPC1 in PC-3M*^trpv6−/−^*-mCherry, PC-3M*^trpv6−/−^*-pTRPV6_wt_, and PC-3M*^trpv6−/−^*-pTRPV6^D582A^ stable cell clones. **(n)** Protein expression of Orai1, Stim1 and TRPC1 in PC-3M-luc-C6*^trpv6+/+^*-mCherry and PC-3M-luc-C6*^trpv6+/+^*-pTRPV6_wt_ stable cell clones. **(o)** Bar plots summarizing of the whole-cell currents during the application of the DVF solution in HEK cells transfected with the plasmids used to generate stable cell lines such as PC-3M*^trpv6−/−^*-mCherry, PC-3M*^trpv6−/−^*-pTRPV6_wt_, and PC-3M*^trpv6−/−^*-pTRPV6^D582A^. Mean ± SEM (**a**, **c**, **f**, **g**, **h**, **j**, **k**, **l, o**). Two-tailed t test (**c**, **j**, **k**, **l, o**). Two-way ANOVA (**a**, **f**, **g**, **h**).

Supplementary Figure 2.

**
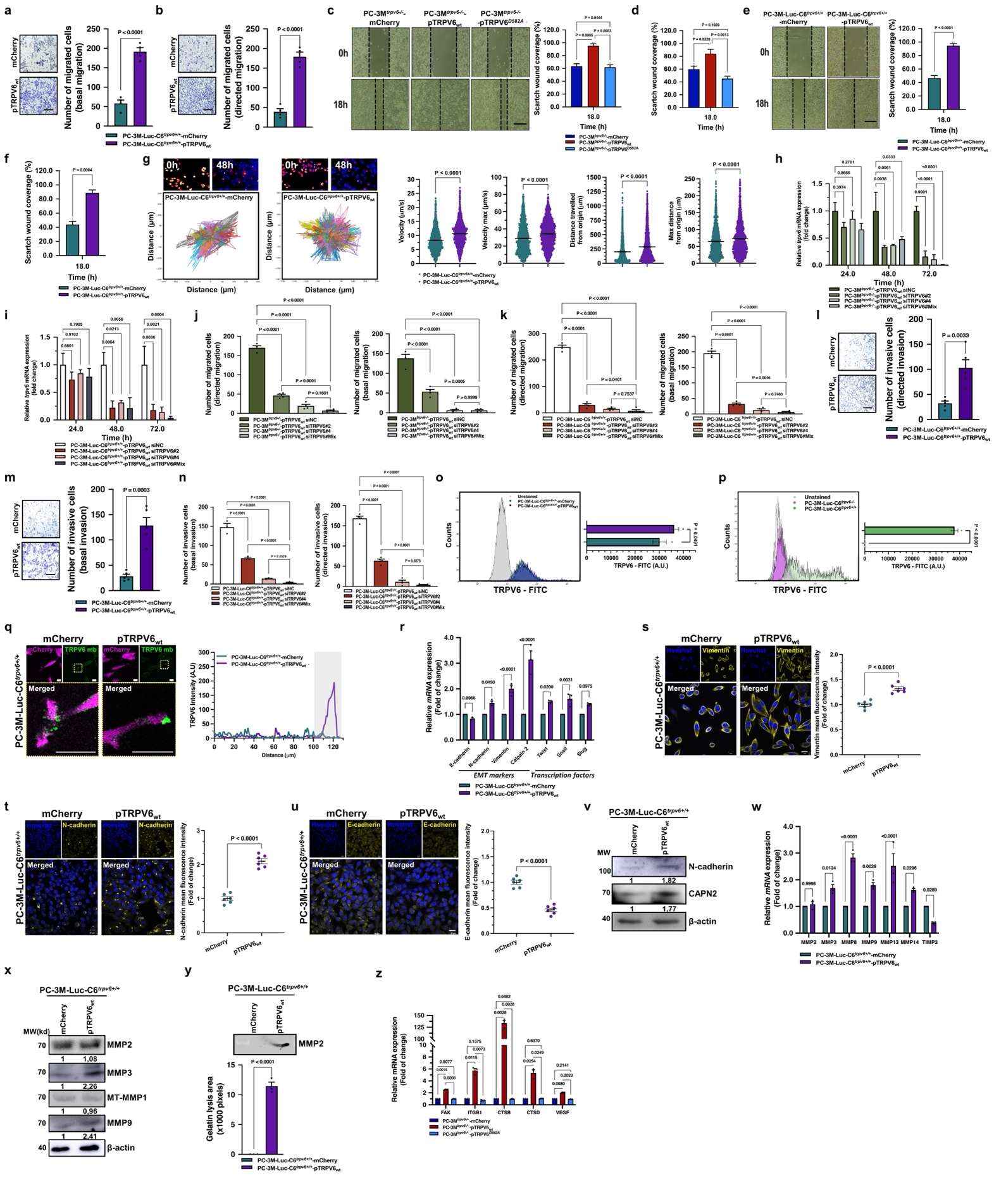
 Supplementary Figure 2. TRPV6 involvement in migration and invasion of PC-3M-luc-C6 cell line *in vitro*.** **(a)**Basal cell migration of PC-3M-luc-C6*^trpv6+/+^*-mCherry and PC-3M-luc-C6*^trpv6+/+^*-pTRPV6_wt_ stable cell clones shown as representative images and quantification of migrated cells (n = 4). Scale bars, 200 µm. **(b)** Directed cell migration of PC-3M-luc-C6*^trpv6+/+^*-mCherry and PC-3M-luc-C6*^trpv6+/+^*-pTRPV6_wt_ stable cell clones shown as representative images and quantification of migrated cells (n = 4). Scale bars, 200 µm. **(c)** Wound-healing migration assay of PC-3M*^trpv6−/−^*-mCherry, PC-3M*^trpv6−/−^*-pTRPV6_wt_, and PC-3M*^trpv6−/−^*-pTRPV6^D582A^ stable cell clones during 18 hours in 2% FBS (n = 4). Scale bars, 200 µm. **(d)** The same experiment that in **(c)** but in 10% FBS (n = 4). **(e)** Wound-healing migration assay of PC-3M-luc-C6*^trpv6+/+^*-mCherry and PC-3M-luc-C6*^trpv6+/+^*-pTRPV6_wt_ stable cell clones during 18 hours in 2% FBS (n = 4). Scale bars, 200 µm. **(f)** The same experiment that in **e.** but in 10% FBS (n = 4). **(g)** Tracking of PC-3M-luc-C6*^trpv6+/+^*-mCherry and PC-3M-luc-C6*^trpv6+/+^*-pTRPV6_wt_ stable cell clones and quantification of both cell velocity and distance (n = 3). **(h)** TRPV6 expression using qPCR in PC-3M*^trpv6-/-^*-pTRPV6*_wt_* transfected with 40 µM siRNA negative control (NC) or siRNA against TRPV6 or a mix of siRNAs against TRPV6 for 48 hours (n = 3). **(i)** TRPV6 expression using qPCR in PC-3M-luc-C6*^trpv6+/+^*-pTRPV6_wt_ stable cell clones transfected with 40 µM siRNA negative control (NC) or siRNA against TRPV6 or a mix of siRNAs against TRPV6 for 48 hours (n = 3). **(j)** Directed and basal migration of PC-3M*^trpv6−/−^*-pTRPV6_wt_ stable cell clone transfected with 40 µM siRNA negative control (NC) or siRNA against TRPV6 or a mix of siRNAs against TRPV6 for 48 hours. Quantification of migrated cells (n = 4). **(k)**Directed and basal migration of PC-3M-luc-C6*^trpv6+/+^*-pTRPV6_wt_ stable cell clone transfected with 40 µM siRNA negative control (NC) or siRNA against TRPV6 or a mix of siRNAs against TRPV6 for 48 hours. Quantification of migrated cells (n = 4). **(l)** Directed invasion of PC-3M-luc-C6*^trpv6+/+^*-mCherry and PC-3M-luc-C6*^trpv6+/+^*-pTRPV6_wt_ stable cell clones. Representative images and quantification of invasive cells (n = 4). Scale bars, 200 µm. **(m)**Basal invasion of PC-3M-luc-C6*^trpv6+/+^*-mCherry and PC-3M-luc-C6*^trpv6+/+^*-pTRPV6_wt_ stable cell clones. Representative images and quantification of invasive cells (n = 4). Scale bars, 200 µm. **(n)** Basal and directed invasion of PC-3M-luc-C6*^trpv6+/+^*-pTRPV6_wt_ stable cell clones transfected with 40 µM siRNA negative control (NC) or siRNA against TRPV6 or a mix of siRNAs against TRPV6 for 48 hours. Quantification of invasive cells (n = 4). **(o)** Flow cytometry of cell surface TRPV6 expression in PC-3M-luc-C6*^trpv6+/+^*-mCherry and PC-3M-luc-C6*^trpv6+/+^*-pTRPV6_wt_ stable cell clones (n=3). **(p)** Flow cytometry of cell surface TRPV6 expression in PC-3M-luc-C6*^trpv6-/-^* and PC-3M-luc-C6*^trpv6+/+^*stable cell clones (n=3). **(q)** TRPV6 channel targeting to the plasma membrane in the regions of cell protrusions in PC-3M-luc-C6*^trpv6+/+^*-mCherry and PC-3M-luc-C6*^trpv6+/+^*-pTRPV6_wt_ stable cell clones. Scale bars, 100 µm. **(r)** EMT markers and transcriptional factors expression using qPCR in PC-3M*^trpv6−/−^*-mCherry, PC-3M*^trpv6−/−^*-pTRPV6_wt_, and PC-3M*^trpv6−/−^*-pTRPV6^D582A^ stable cell clones (n=3). **(s)** Vimentin staining in PC-3M-luc-C6*^trpv6+/+^*-mCherry and PC-3M-luc-C6*^trpv6+/+^*-pTRPV6_wt_ stable cell clones with the quantification of mean intensity reported to the Hoechst staining (n = 6). Scale bar, 20 µm. **(t)** N-cadherin staining in PC-3M-luc-C6*^trpv6+/+^*-mCherry and PC-3M-luc-C6*^trpv6+/+^*-pTRPV6_wt_ stable cell clones with the quantification of mean intensity reported to the Hoechst staining (n = 6). Scale bar, 20 µm. **(u)** E-cadherin staining in PC-3M-luc-C6*^trpv6+/+^*-mCherry and PC-3M-luc-C6*^trpv6+/+^*-pTRPV6_wt_ stable cell clones with the quantification of mean intensity reported to the Hoechst staining (n = 6). Scale bar, 20 µm. **(v)** Protein expression of N-cadherin and calpain 2 in PC-3M-luc-C6*^trpv6+/+^*-mCherry and PC-3M-luc-C6*^trpv6+/+^*-pTRPV6_wt_ stable cell clones (n = 3). **(w)** MMPs and TIMP2 expression using qPCR in PC-3M-luc-C6*^trpv6+/+^*-mCherry and PC-3M-luc-C6*^trpv6+/+^*-pTRPV6_wt_ stable cell clones (n=3). **(x)**Protein expression of MMP2, MMP3, MT-MMP1, and MMP9 proteins in PC-3M-luc-C6*^trpv6+/+^*-mCherry and PC-3M-luc-C6*^trpv6+/+^*-pTRPV6_wt_ stable cell clones (n = 3). **(y)**1% gelatin-zymography analysis and quantification of MMP2 from conditioned media of PC-3M-luc-C6*^trpv6+/+^*-mCherry and PC-3M-luc-C6*^trpv6+/+^*-pTRPV6_wt_ stable cell clones (n = 3). **(z)** FAK, ITGB1, CTSB, CTSD, and VEGF expression detected using qPCR in PC-3M*^trpv6-/-^*-mCherry, PC-3M*^trpv6-/-^*-pTRPV6_wt_ and 3M*^trpv6-/-^*-pTRPV6^D582A^ stable cell clones (n=3). Mean ± SEM (**a-f, h-p, r-u, w, y, z**). Two-sided t test (**a, b, e, f, g, l, m, o, p, r, s, t, u, w, y**). Two-way ANOVA (**c, d, h, i, j, l, n, z**).

Supplementary Figure 3.


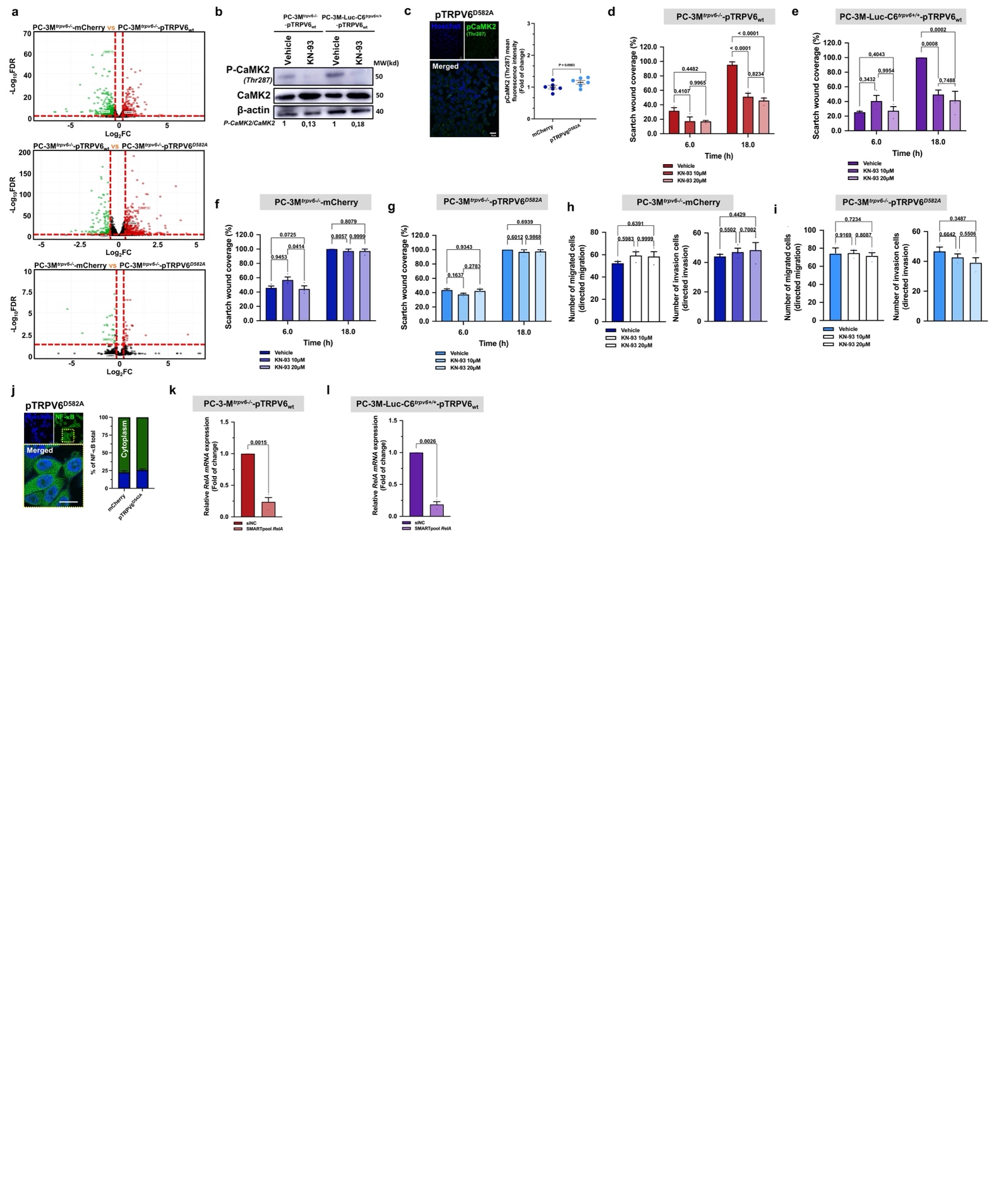


**Supplementary Figure 3. p-CaMK2 and NF-κB/RelA are involved in TRPV6-mediated signaling.** **(a)** Volcano plots showing the differentially expressed genes (DEGs; Log_2_FC > 0,5 or < −0,5, false discovery rate adjusted p-value <0.05) in PC-3M*^trpv6−/−^*-mCherry, PC-3M*^trpv6−/−^*-pTRPV6_wt_, and PC-3M*^trpv6−/−^*-pTRPV6^D582A^ stable cell clones. **(b)** Protein expression of p-CaMK2 (Thr287) in PC-3M*^trpv6−/−^*-pTRPV6_wt_ and PC-3M-luc-C6*^trpv6+/+^*-pTRPV6_wt_ cells treated with 10 μM KN-93 or vehicle. **(c)** p-CaMK2 (Thr287) staining in PC-3M*^trpv6−/−^*-pTRPV6^D582A^ stable cell clones and quantification of mean intensity reported to the Hoechst (n = 6). Scale bar, 20 μm. **(d)** Migration of PC-3M*^trpv6−/−^*-pTRPV6_wt_ cells treated with CaMK2 inhibitor, KN-93 (10 or 20 μM) or Vehicle during 18 hours in 2% FBS using wound-healing assay. **(e)** Migration of PC-3M-luc-C6*^trpv6+/+^*-pTRPV6_wt_ cells treated with CaMK2 inhibitor, KN-93 (10 or 20 μM) or Vehicle during 18 hours in 2% FBS using wound-healing assay. **(f)** Migration of PC-3M*^trpv6−/−^*-mCherry cells treated with CaMK2 inhibitor, KN-93 (10 or 20 μM) or Vehicle during 18 hours in 2% FBS using wound-healing assay. **(g)** Migration of PC-3M*^trpv6−/−^*-pTRPV6^D582A^ treated with CaMK2 inhibitor, KN-93 (10 or 20 μM) or Vehicle during 18 hours in 2% FBS using wound-healing assay. **(h)** Directed migration (left) and invasion (right) of PC-3M*^trpv6−/−^*-mCherry cells treated with CaMK2 inhibitor, KN-93 (10 or 20 μM) or Vehicle. Quantification of migrated and invasive cells (n = 4). **(i)** Directed migration (left) and invasion (right) of PC-3M*^trpv6−/−^*-pTRPV6^D582A^ cells treated with CaMK2 inhibitor, KN-93 (10 or 20 μM) or Vehicle. Quantification of migrated and invasive cells (n = 4). **(j)** NF-κB/RelA staining in PC-3M*^trpv6−/−^*-pTRPV6^D582A^ cells clones with various TRPV6 expression treated either with KN-93 (10 µM) or vehicle and quantification of nuclear-positive cells (n = 6). Scale bar, 20 µm. **(k)** *RelA* expression in PC-3M*^trpv6−/−^*-pTRPV6_wt_ treated with 40 nM of SMARTpool against *RelA* as compared to control siRNA (siNC). **(l)** *RelA* expression in PC-3M-luc-C6*^trpv6+/+^*-pTRPV6_wt_ treated with 40 nM of SMARTpool against *RelA* as compared to control siRNA (siNC). Mean ± SEM (**d, e, f, g, h, i, j, k, l**). Two-sided t test (**j, k, l**). Two-way ANOVA (**d, e, f, g, h, i**).

Supplementary Figure 4.


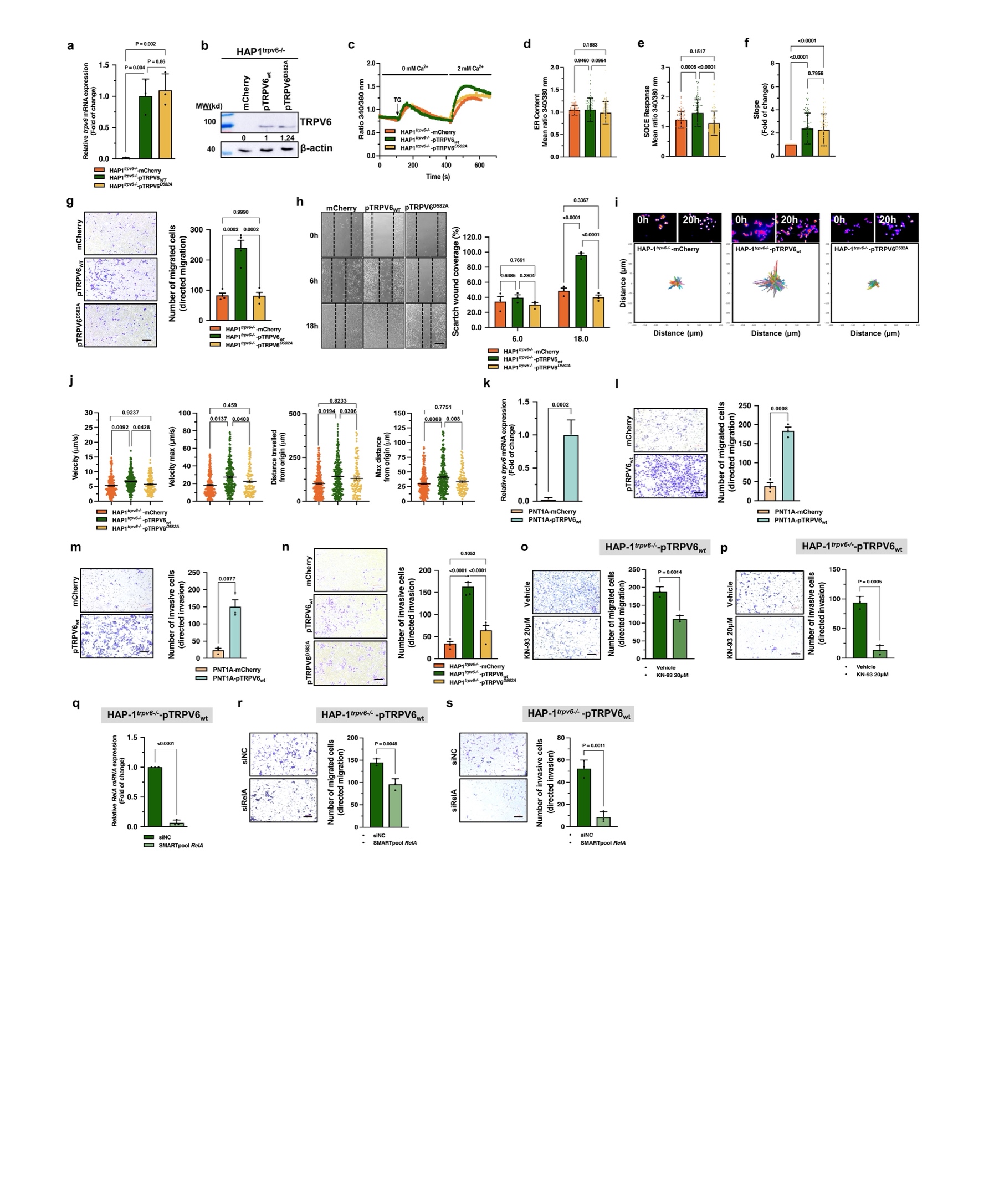


**Supplementary Figure 4. TRPV6 role in migration and invasive potential via CaMK2 and NF-κB pathway in HAP-1 cells. (a)** TRPV6 expression in HAP-1*^trpv6−/−^*-mCherry, HAP-1*^trpv6−/−^*-pTRPV6_WT_, and HAP-1*^trpv6−/−^*-pTRPV6^D582A^ stable cell clones. **(b)** TRPV6 protein expression in HAP-1*^trpv6-/-^* derived stable cell clones. **(c)** Capacitive calcium entry using cytosolic Ca^2+^-imaging (Fura-2 probe) in HAP-1*^trpv6-/-^*derived stable cell clones. **(d)** Fluorescence ratio with mean values of peak amplitudes of the thapsigargin-induced Ca^2+^ release from the ER shown in **c**. **(e)** Fluorescence ratio with mean values of peak amplitudes of SOCE shown in **c**. **(f)** Fluorescence ratio with relative mean values of SOCE slope shown in **c**. **(g)** Directed migration of HAP-1*^trpv6−/−^*-mCherry, HAP-1*^trpv6−/−^*-pTRPV6_WT_, and HAP-1*^trpv6−/−^*-pTRPV6^D582A^ stable cell clones with the representative images and quantification of migrated cells (n = 3). Scale bar, 200 µm. **(h)** Migration of HAP-1*^trpv6−/−^*-mCherry, HAP-1*^trpv6−/−^*-pTRPV6_WT_, and HAP-1*^trpv6−/−^*-pTRPV6^D582A^ stable cell clones during 18 hours in 2% FBS using wound-healing assay. Representative images and quantification (n = 3). Scale bar, 200 µm. **(i)** Tracking of HAP-1*^trpv6−/−^*-mCherry, left (n = 24), HAP-1*^trpv6−/−^*-pTRPV6_WT_, center (n = 93), and HAP-1*^trpv6−/−^*-pTRPV6^D582A^, right (n = 35) stable cell clones for 20 hours. **(j)** Quantification of velocity and distance of HAP-1*^trpv6-/-^* derived stable cell clones from **g**. **(k)** TRPV6 expression in PNT1A cells transiently transfected with either pmCherry or pTRPV6_WT_ plasmids. **(l)** Directed migration of PNT1A cells transiently transfected with either pmCherry or pTRPV6_WT_ plasmids. Image and quantification of migrated cells (n = 3). Scale bar, 200 µm. **(m)** Directed invasion of PNT1A cells transiently transfected with either pmCherry or pTRPV6_WT_ plasmids. Image and quantification of invasion cells (n = 3). Scale bar, 200 µm. **(n)** Directed invasion of HAP-1*^trpv6−/−^*-mCherry, HAP-1*^trpv6−/−^*-pTRPV6_WT_, and HAP-1*^trpv6−/−^*-pTRPV6^D582A^ stable cell clones with the representative images and quantification of invasive cells (n = 3). Scale bar, 200 µm. **(o)** Directed migration of HAP-1*^trpv6-/-^*-pTRPV6_WT_ cells treated with CaMK2 inhibitor, KN-93 (20 μM) or Vehicle. Representative images and quantification of migrated cells (n = 4). Scale bar, 200 µm. **(p)** Directed invasion of HAP-1*^trpv6-/-^*-pTRPV6_WT_ cells treated with CaMK2 inhibitor, KN-93 (20 μM) or Vehicle. Representative images and quantification of invasive cells (n = 4). Scale bar, 200 µm. **(q)** *RelA* expression in HAP-1*^trpv6-/-^*-pTRPV6_WT_ clones transfected with 40 nM SMARTpool against *RelA* as compared to control siRNA (siNC). **(r)** Directed migration of HAP-1*^trpv6-/-^*-pTRPV6_WT_ cells transfected with 40 nM SMARTpool against *RelA* as compared to control siRNA (siNC). Representative images and quantification of migrated cells (n = 4). Scale bar, 200 µm. **(s)** Directed invasion of HAP-1*^trpv6-/^*^-^-pTRPV6_WT_ cells transfected with SMARTpool against *RelA* as compared to control siRNA (siNC). Representative images and quantification of invasive cells (n = 4). Scale bar, 200 µm. Mean ± SEM (**a**, **d**, **e**, **f**, **g, h, j, k, l, m, n, o, p, q, r, s**). Two-sided t test (**k, l, m, o, p, q, r, s**). Two-way ANOVA (**a**, **d**, **e**, **f**, **g,** **h**, **j**, **n**).

Supplementary Figure 5.


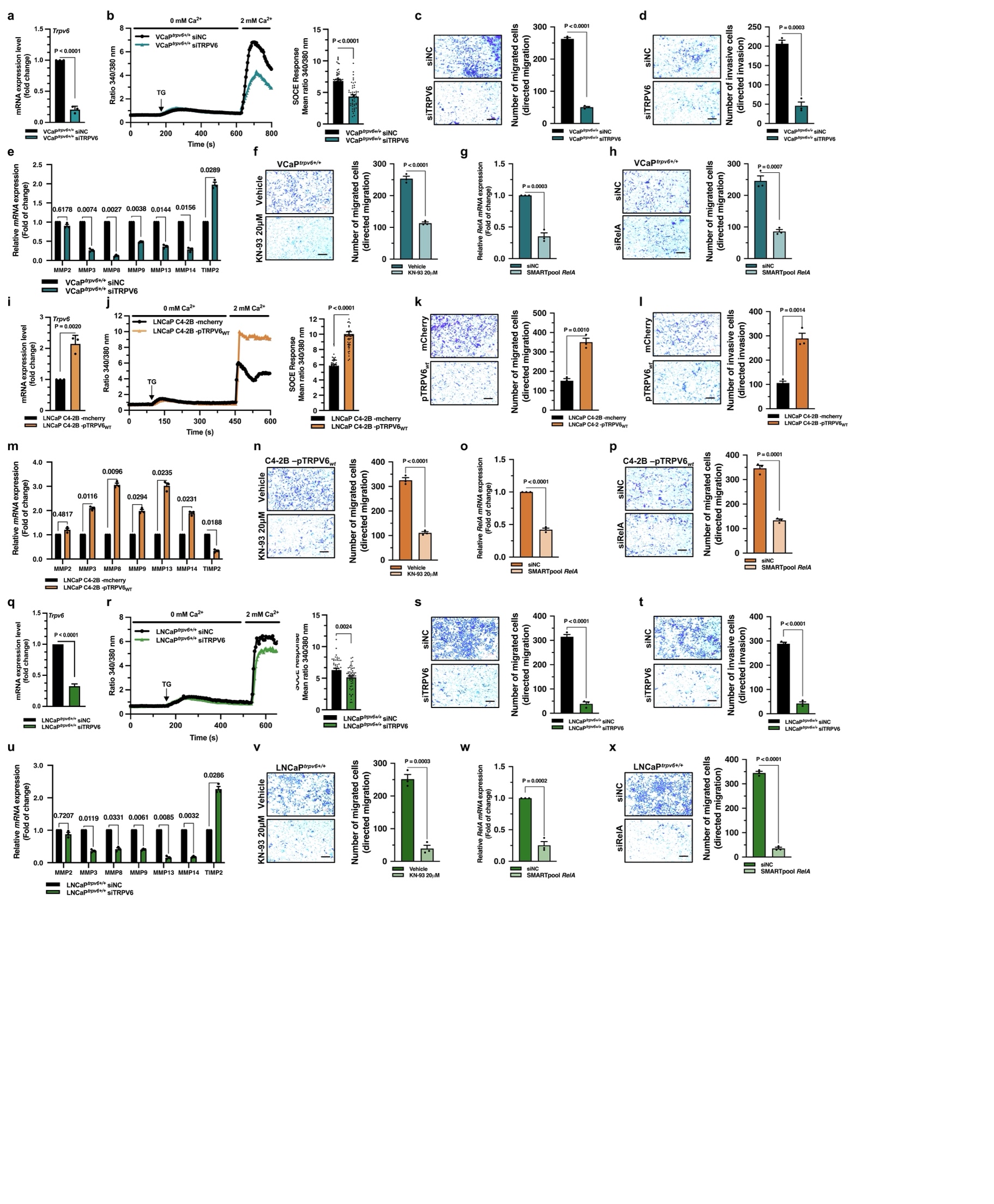


**Supplementary Figure 5. TRPV6 is involved in migration and invasive potential via CaMK2 and NF-κB pathway in CRPC and CSPC cells. (a)** TRPV6 expression in VCaP*^trpv6+/+^* transfected with either siNC or siTRPV6 mix. **(b)** Capacitive calcium entry using cytosolic Ca^2+^-imaging (Fura-2 probe) in VCaP*^trpv6+/+^* cells and fluorescence ratio with mean values of peak amplitudes of SOCE. **(c)** Representative images and quantification of migrated cells involved in directed migration of VCaP*^trpv6+/+^* transfected with either siNC or siTRPV6 mix (n = 3). Scale bar, 200 µm. **(d)** Representative images and quantification of invasive cells involved in directed invasion of VCaP*^trpv6+/+^* transfected with either siNC or siTRPV6 mix (n = 3). Scale bar, 200 µm. **(e)** MMPs and TIMP2 expression using qPCR in VCaP*^trpv6+/+^* transfected cells (n=3). **(f)** Representative images and quantification of migrated cells involved in directed migration of VCaP*^trpv6+/+^* cells treated with CaMK2 inhibitor, KN-93 (20 μM) or Vehicle (n = 3). Scale bar, 200 µm. **(g)** *RelA* expression in VCaP*^trpv6+/+^* cells transfected with 40 nM SMARTpool against *RelA* as compared to control siRNA (siNC). **(h)** Representative images and quantification of migrated cells involved in directed migration of VCaP*^trpv6+/+^* cells transfected with 40 nM SMARTpool against *RelA* as compared to control siRNA (siNC) (n = 3). Scale bar, 200 µm. **(i)** TRPV6 expression in both LNCaP-C4-2B*^trpv6+/+^*-mCherry and LNCaP-C4-2B*^trpv6+/+^*- pTRPV6_wt_ stable cell clones. **(j)** Capacitive calcium entry using cytosolic Ca^2+^-imaging (Fura-2 probe) in LNCaP-C4-2B*^trpv6+/+^* derived cells and fluorescence ratio with mean values of peak amplitudes of SOCE. **(k)** Representative images and quantification of migrated cells involved in directed migration of both LNCaP-C4-2B*^trpv6+/+^*- mCherry and LNCaP-C4-2B*^trpv6+/+^*-pTRPV6_wt_ stable cell clones (n = 3). Scale bar, 200 µm. **(l)** Representative images and quantification of invasive cells involved in directed invasion of both LNCaP-C4-2B*^trpv6+/+^*-mCherry and LNCaP-C4-2B*^trpv6+/+^*- pTRPV6_wt_ stable cell clones (n = 3). Scale bar, 200 µm. **(m)** MMPs and TIMP2 expression using qPCR in both LNCaP-C4-2B*^trpv6+/+^*-mCherry and LNCaP-C4-2B*^trpv6+/+^*- pTRPV6_wt_ stable cell clones (n=3). **(n)** Representative images and quantification of migrated cells involved in directed migration of LNCaP-C4-2B*^trpv6+/+^*-pTRPV6_wt_ stable cell clones, treated with either CaMK2 inhibitor, KN-93 (20 μM) or Vehicle (n = 3). Scale bar, 200 µm. **(o)** *RelA* expression in LNCaP-C4-2B*^trpv6+/+^*- pTRPV6_wt_ stable cell clones transfected with 40 nM SMARTpool against *RelA* as compared to control siRNA (siNC). **(p)** Representative images and quantification of migrated cells involved in directed migration of LNCaP-C4-2B*^trpv6+/+^*-pTRPV6_wt_ stable cell clones transfected with 40 nM SMARTpool against *RelA* as compared to control siRNA (siNC) (n = 3). Scale bar, 200 µm. **(q)** TRPV6 expression in LNCaP*^trpv6+/+^* transfected with either siNC or siTRPV6 mix. **(r)** Capacitive calcium entry tracked using cytosolic Ca^2+^-imaging (Fura-2 probe) in LNCaP*^trpv6+/+^* cells fluorescence ratio with mean values of peak amplitudes of SOCE. **(s)** Representative images and quantification of migrated cells involved in directed migration of LNCaP*^trpv6+/+^* cells transfected with either siNC or siTRPV6 mix (n = 3). Scale bar, 200 µm. **(t)** Representative images and quantification of invasive cells involved in directed invasion of LNCaP*^trpv6+/+^* cells transfected with either siNC or siTRPV6 mix (n = 3). Scale bar, 200 µm. **(u)** MMPs and TIMP2 expression using qPCR in LNCaP*^trpv6+/+^* transfected cells (n=3). **(v)** Representative images and quantification of migrated cells involved in directed migration of LNCaP*^trpv6+/+^* cells treated with CaMK2 inhibitor, KN-93 (20 μM) or Vehicle. (n = 3). Scale bar, 200 µm. **(w)** *RelA* expression in LNCaP*^trpv6+/+^* transfected with 40 nM SMARTpool against *RelA* as compared to control siRNA (siNC). **(x)** Representative images and quantification of migrated cells involved in directed migration of LNCaP*^trpv6+/+^* cells transfected with 40 nM SMARTpool against *RelA* as compared to control siRNA (siNC) (n = 3). Scale bar, 200 µm. Mean ± SEM (**a-x)**. Two-sided t test (**a-x**).

Supplementary Figure 6.


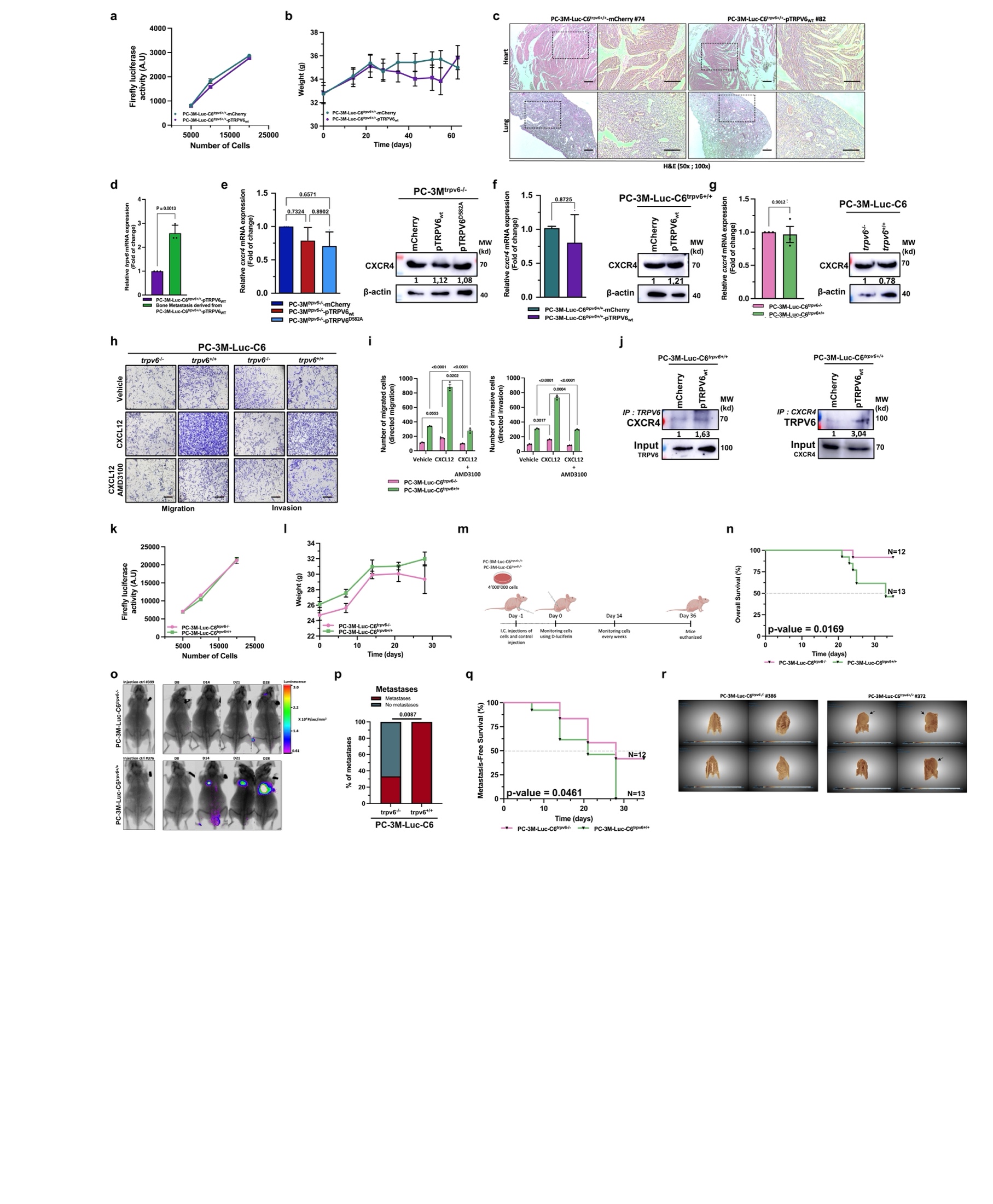


**Supplementary Figure 6. TRPV6 and CXCR4 interaction. (a)** Firefly luciferase activity in PC-3M-luc-C6*^trpv6+/+^*-mCherry and PC-3M-luc-C6*^trpv6+/+^*-pTRPV6*_WT_* stable clones. **(b)** Weight growth of mice bearing either PC-3M-luc-C6*^trpv6+/+^*-mCherry (n = 14) or PC-3M-luc-C6*^trpv6+/+^*-pTRPV6_wt_ (n = 14) stable clones grafted *via* intra-cardiac injections. **(c)** Representative H&E staining of both heart and lung tissues from mice bearing PC-3M-luc-C6*^trpv6+/+^*-mCherry and PC-3M-luc-C6*^trpv6+/+^*-pTRPV6_wt_ cell clones grafted *via* intra-cardiac injections. Scale bars, 100 μm. **(d)** TRPV6 expression in PC-3M-luc-C6*^trpv6+/+^*-pTRPV6_wt_ cells at the moment of injection versus the derived bone metastasis from the same mouse. **(e)** CXCR4 expression both at mRNA (left) and protein (right) level in PC-3M*^trpv6−/−^*-mCherry, PC-3M*^trpv6−/−^*-pTRPV6_WT_, and PC-3M*^trpv6−/−^*-pTRPV6^D582A^ stable cell clones. **(f)** CXCR4 expression both at mRNA (left) and protein (right) level in PC-3M-luc-C6*^trpv6+/+^*-mCherry versus PC-3M-luc-C6*^trpv6+/+^*-pTRPV6*_WT_* stable cell clones. **(g)** CXCR4 expression both at mRNA (left) and protein (right) level in PC-3M-luc-C6*^trpv6-/-^* versus PC-3M-luc-C6*^trpv6+/+^* cells. **(h)** Directed migration and invasion of PC-3M-luc-C6*^trpv6-/-^* versus PC-3M-luc-C6*^trpv6+/+^* cells treated with CXCR4 inhibitor, AMD3100 (30 μM) or Vehicle, or 100 ng/ml CXCL12 as chemoattractant. Scale bar, 200 µm. **(i)** Quantification of the number of migrated and invaded through the matrix cells (n = 3). **(j)** Co-immunoprecipitation of CXCR4 receptor using anti-TRPV6 antibody and co-immunoprecipitation of TRPV6 using anti-CXCR4 antibody in PC-3M-luc-C6*^trpv6-/-^* versus PC-3M-luc-C6*^trpv6+/+^* cells. **(k)** Firefly luciferase activity in PC-3M-luc-C6*^trpv6-/-^* versus PC-3M-luc-C6*^trpv6+/+^* cells. **(l)** Weight growth of mice bearing PC-3M-luc-C6*^trpv6-/-^* (n = 12) versus PC-3M-luc-C6*^trpv6+/+^* (n = 13) cells. (**m**) Timeline of the experimental design of bone metastasis model using both PC-3M-luc-C6*^trpv6-/-^* and PC-3M-luc-C6*^trpv6+/+^* cells expressing firefly luciferase in swiss-nude mice. I.C.: intra-cardiac. **(n)** Overall survival of mice bearing PC-3M-luc-C6*^trpv6-/-^* (n = 12) versus PC-3M-luc-C6*^trpv6+/+^* (n = 13) grafted cells using log rank (Mantel-Cox) test. **(o)** Representative bioluminescence and X-ray imaging of metastasis emerging in mice bearing PC-3M-luc-C6*^trpv6-/-^* (n = 12) versus PC-3M-luc-C6*^trpv6+/+^* (n = 13) grafted cells. **(p)** Percentage of metastasis incidence in mice bearing PC-3M-luc-C6*^trpv6-/-^* (n = 12) versus PC-3M-luc-C6*^trpv6+/+^* (n = 13) grafted cells introduced *via* intra-cardiac injections. **(q)** Metastasis-free survival of PC-3M-luc-C6*^trpv6-/-^* (n = 12) versus PC-3M-luc-C6*^trpv6+/+^*(n = 13) mice using log rank (Mantel-Cox) test. **(r)** Representative 3D-reconstruction image of mice metastasis in the ribs under different angle of views in mice bearing PC-3M-luc-C6*^trpv6-/-^* versus PC-3M-luc-C6*^trpv6+/+^* grafted cells introduced *via* intra-cardiac injections. Mean ± SEM (**a**, **b**, **d**, **e**, **f**, **g**, **i**, **k, l, p**). Two-tailed t test (**a**, **b**, **d**, **f**, **g**, **p**). Two-way ANOVA (**e**, **i**). Log rank (Mantel-Cox) test (**n**, **q**).

Supplementary Figure 7.


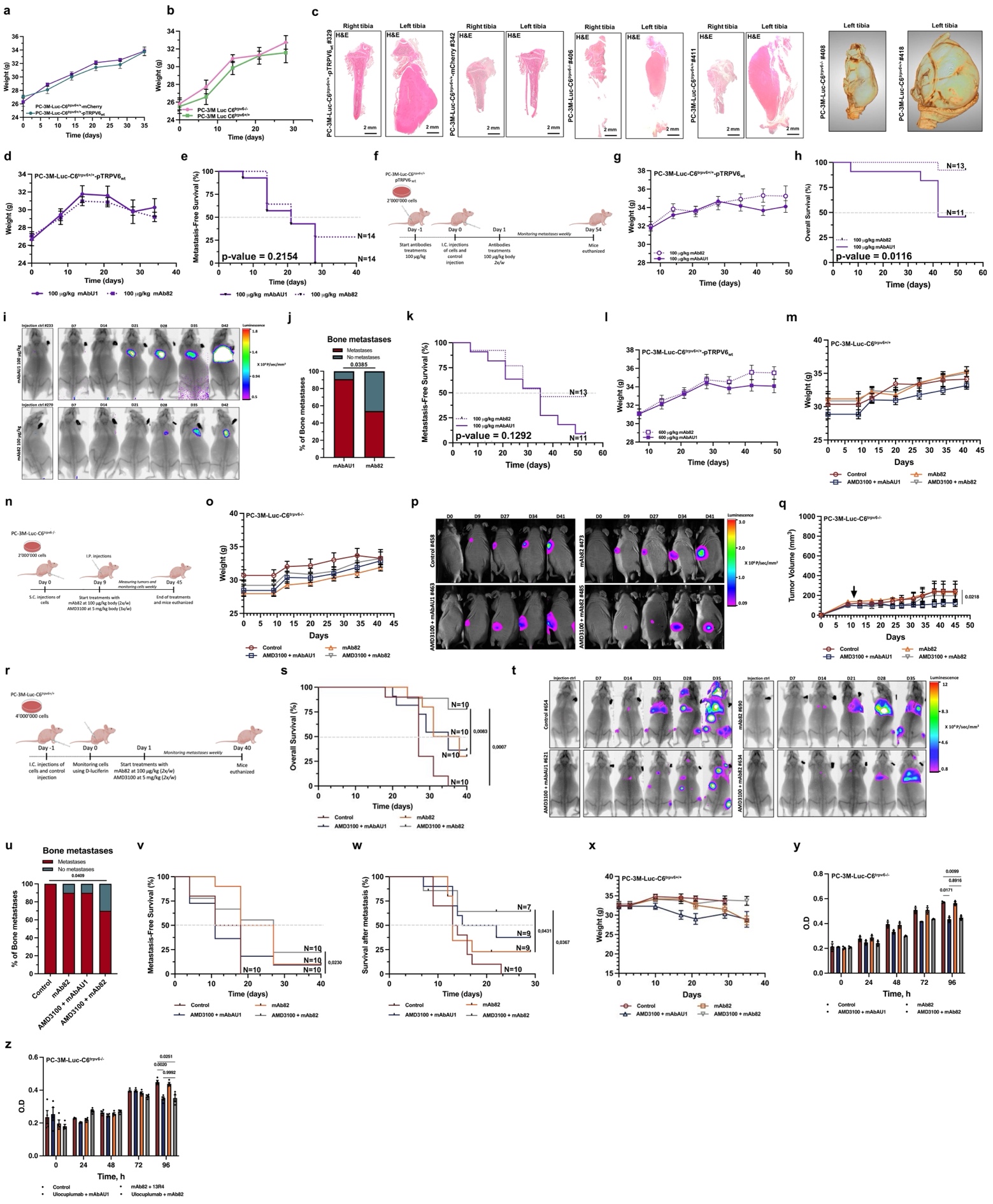


**Supplementary Figure 7. Bone metastasis formation and treatment with anti-TRPV6 monoclonal antibody in vivo. (a)** Weight growth of mice bearing the xenograft of either PC-3M-luc-C6*^trpv6+/+^*-mCherry or PC-3M-luc-C6*^trpv6+/+^*-pTRPV6_wt_ cell clones grafted *via* intra-osseous injection. **(b)** Weight growth of mice bearing the xenograft of either PC-3M-luc-C6*^trpv6-/-^* or PC-3M-luc-C6*^trpv6+/+^*cells *via* intra-osseous injection. **(c)** Representative H&E staining of tibia from mice bearing the xenograft of either PC-3M-luc-C6*^trpv6-/-^* or PC-3M-luc-C6*^trpv6+/+^*or PC-3M-luc-C6*^trpv6+/+^*-mCherry or PC-3M-luc-C6*^trpv6+/+^*-pTRPV6_wt_ cell clones grafted *via* intra-osseous injection and representative 3D-reconstruction images of mice tibia metastasis derived from PC-3M-luc-C6*^trpv6-/-^* or PC-3M-luc-C6*^trpv6+/+^*grafted cells. Scale bars, 2 mm. **(d)**Weight growth of mice bearing PC-3M-luc-C6*^trpv6+/+^*-pTRPV6_wt_ cell clones and treated with either 100 µg/kg mAbAU1 (n = 14) or mAb82 (n = 14) 24 hours following intra-cardiac cell injections using log rank (Mantel-Cox) test. **(e)** Metastasis-free survival of mice bearing PC-3M-luc-C6*^trpv6+/+^*-pTRPV6_wt_ cell clones and treated with either 100 µg/kg mAbAU1 (n = 14) or mAb82 (n = 14) 24 hours following intra-cardiac cell injections using log rank (Mantel-Cox) test.**(f)** Timeline of the experimental bone metastasis model using swiss mice being grafted with PC-3M-luc-C6*^trpv6+/+^*-pTRPV6_wt_ cell clones stably expressing firefly luciferase and treated with either 100 µg/kg mAbAU1 or mAb82 24 hours before intra-cardiac cell injections and followed by the treatment with either 100 µg/kg mAbAU1 or mAb82 twice per week starting 24 hours after intra-cardiac cell injections. I.C.: intra-cardiac. I.P.: intra-peritoneal. **(g)**Weight growth of mice bearing PC-3M-luc-C6*^trpv6+/+^*-pTRPV6_wt_ cell clones treated with either 100 µg/kg mAbAU1 (n = 13) or mAb82 (n =11) 24 hours before intra-cardiac injections and followed by the treatment with either 100 µg/kg mAbAU1 or mAb82 twice per week starting 24 hours after intra-cardiac cell injections. **(h)** Metastasis-free survival of mice bearing PC-3M-luc-C6*^trpv6+/+^*-pTRPV6_wt_ cell clones treated with either 100 µg/kg mAbAU1 (n = 13) or mAb82 (n =11) 24 hours before intra-cardiac injections and followed by the treatment with either 100 µg/kg mAbAU1 or mAb82 twice per week starting 24 hours after intra-cardiac cell injections using log rank (Mantel-Cox) test. **(i)**Representative bioluminescence and X-ray imaging of mice bearing PC-3M-luc-C6*^trpv6+/+^*-pTRPV6_wt_ cell clones treated with either 100 µg/kg mAbAU1 (n = 13) or mAb82 (n =11) 24 hours before intra-cardiac injections and followed by the treatment with either 100 µg/kg mAbAU1 or mAb82 twice per week starting 24 hours after intra-cardiac cell injections. **(j)** Incidence of bone metastasis in mice bearing PC-3M-luc-C6*^trpv6+/+^*-pTRPV6_wt_ cell clones treated with either 100 µg/kg mAbAU1 (n = 13) or mAb82 (n =11) 24 hours before intra-cardiac injections and followed by the treatment with either 100 µg/kg mAbAU1 or mAb82 twice per week starting 24 hours after intra-cardiac cell injections using log rank (Mantel-Cox) test. **(k)**Overall survival of mice bearing PC-3M-luc-C6*^trpv6+/+^*-pTRPV6_wt_ cell clones treated with either 100 µg/kg mAbAU1 (n = 11) or mAb82 (n =13) 24 hours before intra-cardiac injections and followed by the treatment with either 100 µg/kg mAbAU1 or mAb82 twice per week starting 24 hours after intra-cardiac cell injections using log rank (Mantel-Cox) test. **(l)**Weight growth of mice bearing PC-3M-luc-C6*^trpv6+/+^*-pTRPV6_wt_ cell clones treated with either 600 µg/kg mAbAU1 (n = 12) or mAb82 (n =12) 24 hours before intra-cardiac injections and followed by the treatment with either 100 µg/kg mAbAU1 or mAb82 twice per week starting 24 hours after intra-cardiac cell injections.**(m)** Weight growth of mice bearing the xenograft of PC-3M-luc-C6*^trpv6+/+^*cells stably expressing luciferase in swiss-nude mice treated with either 100 µg/kg AU1 or mAb82 together with 5 mg/kg AMD3100 following tumor formation. **(n)** Timeline of the experimental tumor growth model using the xenograft of PC-3M-luc-C6*^trpv6-/-^*cells stably expressing luciferase in swiss-nude mice treated with either 100 µg/kg AU1 or mAb82 together with 5 mg/kg AMD3100 following tumor formation. S.C.: sub-cutaneous. I.P.: intra-peritoneal. **(o)** Weight growth of mice bearing the xenograft of PC-3M-luc-C6*^trpv6-/-^*cells stably expressing luciferase in swiss-nude mice treated with either 100 µg/kg AU1 or mAb82 together with 5 mg/kg AMD3100 following tumor formation. **(p)** Representative bioluminescence imaging of mice bearing the xenograft of PC-3M-luc-C6*^trpv6-/-^*cells stably expressing luciferase in swiss-nude mice treated with either 100 µg/kg mAbAU1 or mAb82 together with 5 mg/kg AMD3100 following tumor formation.**(q)** Tumor growth in mice bearing the xenograft of PC-3M-luc-C6*^trpv6-/-^*cells stably expressing luciferase in swiss-nude mice treated with either 100 µg/kg mAbAU1 or mAb82 together with 5 mg/kg AMD3100 following tumor formation. **(r)** Timeline of the experimental bone metastasis model using swiss mice being grafted i.c. with PC-3M-luc-C6*^trpv6+/+^* cells stably expressing firefly luciferase and treated with either 100 µg/kg AU1 or mAb82 together with 5 mg/kg AMD3100 twice per week starting 48 hours after intra-cardiac cell injections. I.C.: intra-cardiac. **(s)** Overall survival of mice bearing PC-3M-luc-C6*^trpv6+/+^* cells and treated with either 100 µg/kg AU1 or mAb82 together with 5 mg/kg AMD3100 twice per week starting 48 hours after intra-cardiac cell injections (n = 10 per group), using log rank (Mantel-Cox) test. **(t)** Representative bioluminescence imaging and X-ray of mice being grafted with PC-3M-luc-C6*^trpv6+/+^* cells and treated with either 100 µg/kg AU1 or mAb82 together with 5 mg/kg AMD3100 twice per week starting 48 hours after intra-cardiac cell injections. **(u)** Incidence of bone metastasis in mice grafted with PC-3M-luc-C6*^trpv6+/+^* cells and treated with either 100 µg/kg AU1 or mAb82 together with 5 mg/kg AMD3100 twice per week starting 48 hours after intra-cardiac cell injections. **(v)** Metastasis-free survival of mice bearing PC-3M-luc-C6*^trpv6+/+^* cells and treated with either 100 µg/kg AU1 or mAb82 together with 5 mg/kg AMD3100 twice per week starting 48 hours after intra-cardiac cell injections, using log rank (Mantel-Cox) test. **(w)** Survival following metastasis appearance of mice bearing PC-3M-luc-C6*^trpv6+/+^* cells and treated with either 100 µg/kg AU1 or mAb82 together with 5 mg/kg AMD3100 twice per week starting 48 hours after intra-cardiac cell injections, using log rank (Mantel-Cox) test. **(x)** Weight growth of mice bearing the xenografts of PC-3M-luc-C6*^trpv6+/+^* cells stably expressing luciferase in swiss-nude mice treated with either 100 µg/kg AU1 or mAb82 together with 5 mg/kg AMD3100 twice per week starting 48 hours after intra-cardiac cell injections. **(y)** Cell survival assay (MTS) of PC-3M-luc-C6*^trpv6-/-^* cells treated with either 6 µg/mL mAbAU1/mAb82 together with 30 mM AMD3100 during 4 days. **(z)** Cell survival assay (MTS) of PC-3M-luc-C6*^trpv6-/-^* cells treated either with 6 µg/mL Ulocuplumab or mAb82 or mAbAU1, as a control, during 4 days. Mean ± SEM (**a, b, d, g, l, m, o, p, x, y, z**). Two-sided t test (**a, b, d, g, j, l**). Two-way ANOVA (**m, o, q, u, x, y, z**). Log rank (Mantel-Cox) test (**e, h, k, s, v, w**).

**Supplementary Table 1. Summary of all cell lines**

| **Cell line** | **Clones** | **AR status** | **TRPV6 status** | **mCherry status** | **Luciferase status** |
| --- | --- | --- | --- | --- | --- |

| **HAP-1** | HAP-1*^trpv6-/-^* -mCherry | *AR-* | *Knockout* | *Positive* | *Absent* |
| --- | --- | --- | --- | --- | --- |
|  | HAP-1*^trpv6-/-^* -pTRPV6*_wt_* |  | *Rescue* | *Positive* | *Absent* |
|  | HAP-1*^trpv6-/-^* -pTRPV6*^D582A^* |  | *Rescue no-functional* | *Positive* | *Absent* |
| **HEK** | HEK*^trpv6+/+^* -mCherry | *AR-* | *Wild Type* | *Positive* | *Absent* |
|  | HEK*^trpv6+/+^* -pTRPV6*_wt_* |  | *Overexpression* | *Positive* | *Absent* |
|  | HEK*^trpv6+/+^* -pTRPV6*^D582A^* |  | *Overexpression no-functional* | *Positive* | *Absent* |
| **PC-3M** | PC-3M*^trpv6-/-^* -mCherry | *AR-*  *CRPC* | *Knockout* | *Positive* | *Absent* |
|  | PC-3M*^trpv6-/-^* -pTRPV6*_wt_* |  | *Rescue* | *Positive* | *Absent* |
|  | PC-3M*^trpv6-/-^* -pTRPV6*^D582A^* |  | *Rescue no-functional* | *Positive* | *Absent* |
|  | PC-3M*^trpv6+/+^* |  | *Wild type* | *Absent* | *Absent* |
|  | PC-3M*^trpv6-/-^* |  | *Knockout* | *Absent* | *Absent* |
| **PC-3M-Luc-C6** | PC-3M-Luc-C6*^trpv6+/+^* | *AR-*  *CRPC* | *Wild type* | *Absent* | *Positive* |
|  | PC-3M-Luc-C6*^trpv6-/-^* |  | *Knockout* | *Absent* | *Positive* |
|  | PC-3M-Luc-C6*^trpv6+/+^* -mCherry |  | *Wild Type* | *Positive* | *Positive* |
|  | PC-3M-Luc-C6*^trpv6+/+^* -pTRPV6*_wt_* |  | *Overexpression* | *Positive* | *Positive* |
| **VCaP** | VCaP*^trpv6+/+^* | *AR++ / AR-V7*  *CRPC* | *Wild type* | *Absent* | *Absent* |
| **LNCaP-C4-2B** | LNCaP-C4-2B*^trpv6+/+^* -mCherry | *AR+ / AR^T877A^*  *CRPC* | *Wild type* | *Positive* | *Absent* |
|  | LNCaP-C4-2B*^trpv6+/+^* -pTRPV6*_wt_* |  | *Overexpression* | *Positive* | *Absent* |
| **LNCaP** | LNCaP*^trpv6+/+^* | *AR+*  *CSPC* | *Wild type* | *Absent* | *Absent* |
| **PNT1A** | PNT1A*^trpv6+/+^* -mCherry | *AR+* | *Wild type* | *Positive* | *Absent* |
|  | PNT1A*^trpv6+/+^* -pTRPV6*_wt_* |  | *Overexpression* | *Positive* | *Absent* |

**Supplementary Table 2. List of primers**

| SPECIES | TARGET | SEQUENCE |
| --- | --- | --- |

| Human | *TRPV6* | *Forward 5’-…-3’* | CCCAAGGAGAAAGGGCTAAT |
| --- | --- | --- | --- |
|  |  | *Backward 5’-…-3’* | TTGGCAGCTAGAAGGAGAGG |
| Human | *MMP2* | *Forward 5’-…-3’* | TTTCCAGCAATGAGAAACTC |
|  |  | *Backward 5’-…-3’* | GTATCTCCAGAATTTGTCTCC |
| Human | *MMP3* | *Forward 5’-…-3’* | GGAAGCTGGACTCCGACACTC |
|  |  | *Backward 5’-…-3’* | TGGTGTATAATTCACAATCCTGTATGTAA |
| Human | *MMP8* | *Forward 5’-…-3’* | TGGACCCAATGGAATCCTTGC |
|  |  | *Backward 5’-…-3’* | ATAGCCACTCAGAGCCCAGTA |
| Human | *MMP9* | *Forward 5’-…-3’* | CTTAGATCATTCCTCAGTGC |
|  |  | *Backward 5’-…-3’* | CGAGGACCATAGAGGTG |
| Human | *MMP13* | *Forward 5’-…-3’* | TTGTTGCTGCGCATGAGTTCG |
|  |  | *Backward 5’-…-3’* | GGGTGCTCATATGCAGCATCA |
| Human | *MMP14*  (MT-MMP1) | *Forward 5’-…-3’* | CGCTACGCCATCCAGGGTCTCAAA |
|  |  | *Backward 5’-…-3’* | CGGTCATCATCGGGCAGCACAAAA |
| Human | *TIMP2* | *Forward 5’-…-3’* | GAGCCTGAACCACAGGTACCA |
|  |  | *Backward 5’-…-3’* | TCTGTGACCCAGTCCATCCA |
| Human | *CDH1*  (E-Cadherin) | *Forward 5’-…-3’* | GAACGCATTGCCACATACAC |
|  |  | *Backward 5’-…-3’* | GAATTCGGGCTTGTTGTCAT |
| Human | *CDH2*  (N-Cadherin) | *Forward 5’-…-3’* | CCTGAGGGATCAAAGCCTGGAAC |
|  |  | *Backward 5’-…-3’* | TTGGAGCCTGAGACACGATTCTG |
| Human | *VIM*  (Vimentin) | *Forward 5’-…-3’* | TGTCCAAATCGATGTGGATGTTTC |
|  |  | *Backward 5’-…-3’* | TTGTACCATTCTTCTGCCTCCTG |
| Human | *CAPN2*  (Calpain 2) | *Forward 5’-…-3’* | TCAGAAGGCTGTTTGCTCAG |
|  |  | *Backward 5’-…-3’* | GCGCTTGGCTAGAACTCTTC |
| Human | *SNAI1*  (Snail) | *Forward 5’-…-3’* | CTTCCAGCAGCCCTACGAC |
|  |  | *Backward 5’-…-3’* | CGGTGGGGTTGAGGATCT |
| Human | *SNAI2*  (Slug) | *Forward 5’-…-3’* | TGTTTGCAAGATCTGCGGC |
|  |  | *Backward 5’-…-3’* | TGCAGTCAGGGCAAGAAAAA |
| Human | *TWIST1* | *Forward 5’-…-3’* | AGCAAGATTCAGACCCTCAAGCT |
|  |  | *Backward 5’-…-3’* | CCTGGTAGAGGAAGTCGATGTACCT |
| Human | *CXCR4* | *Forward 5’-…-3’* | ACTACACCGAGGAAATGGGCT |
|  |  | *Backward 5’-…-3’* | CCCACAATGCCAGTTAAGAAGA |
| Human | *CXCL12* | *Forward 5’-…-3’* | ATTCTCAACACTCCAAACTGTGC |
|  |  | *Backward 5’-…-3’* | ACTTTAGCTTCGGGTCAATGC |
| Human | *BMP2* | *Forward 5’-…-3’* | ATGGATTCGTGGTGGAAGTG |
|  |  | *Backward 5’-…-3’* | GTGGAGTTCAGATGATCAGC |
| Human | *BMP4* | *Forward 5’-…-3’* | AGCATGTCAGGATTAGCCGA |
|  |  | *Backward 5’-…-3’* | TGGAGATGGCACTCAGTTCA |
| Human | *BMP6* | *Forward 5’-…-3’* | CAGCCTGCAGGAAGCATGAG |
|  |  | *Backward 5’-…-3’* | CAAAGTAAAGAACCGAGATG |
| Human | *RELA* | *Forward 5’-…-3’* | CAACCCCTTCCAAGTTCCTATAGA |
|  |  | *Backward 5’-…-3’* | CCTGCCTGATGGGTCCC |
| Human | *FAK* | *Forward 5’-…-3’* | TGGGCGGAAAGAAATCCTGC |
|  |  | *Backward 5’-…-3’* | GGCTTGACACCCTCGTTGTA |
| Human | *ITGB1* | *Forward 5’-…-3’* | AAATGTAACCAACCGTAGC |

|  |  | *Backward 5’-…-3’* | GACAGGTCCATAAGGTAGTAGA |
| --- | --- | --- | --- |

| Human | *CTSB* | *Forward 5’-…-3’* | GATCATGTGGCAGCTCTGGGCCTCCCTCTG |
| --- | --- | --- | --- |

|  |  | *Backward 5’-…-3’* | GTCTTAGATCTTTTCCCAGTACTGATCGG |
| --- | --- | --- | --- |

| Human | *CTSD* | *Forward 5’-…-3’* | TTGCTGTTTTGTTCTGTGGTTTTC |
| --- | --- | --- | --- |

|  |  | *Backward 5’-…-3’* | CAGACAGGCAGGCAGCATT |
| --- | --- | --- | --- |

| Human | *VEGF* | *Forward 5’-…-3’* | GAGCCTTGCCTTGCTGCTCTA |
| --- | --- | --- | --- |
|  |  | *Backward 5’-…-3’* | CACCAGGGTCTCGATTGGATG |
| Human | *GAPDH* | *Forward 5’-…-3’* | ACCCACTCCTCCACCTTTG |
|  |  | *Backward 5’-…-3’* | CTGTTGTGCTCTTGCTGGG |

Data S1. (separate file)

Movies from videomicroscopy and python code.

Data S2. (separate file)

Table of Differential expressed genes.

Data S3. (separate file)

Movies of tibia from intra-osseous injection.
